# Endogenous retrovirus control of calbindin is essential for human early embryogenesis

**DOI:** 10.1126/sciadv.aee4523

**Published:** 2026-10-07

**Authors:** Judith Pape, Afshan McCarthy, Eduardo Serna-Morales, Borzo Gharibi, Tobias Plowman, Elias Copin, Rachael Thompson, Laura Doglio, Ruta Meleckyte-Martinez, Lyn Healy, Sara Tamagno, Alberto Elosegui-Artola, Silvia D. M. Santos, Kathy K. Niakan, George Kassiotis

**Affiliations:** ^1^ Retroviral Immunology Laboratory, The Francis Crick Institute, 1 Midland Road, London NW1 1AT, UK.; ^2^ Human Embryo and Stem Cell Laboratory, The Francis Crick Institute, 1 Midland Road, London NW1 1AT, UK.; ^3^ Cell and Tissue Mechanobiology Laboratory, The Francis Crick Institute, 1 Midland Road, London NW1 1AT, UK.; ^4^ Department of Physics, King’s College London, London, UK.; ^5^ Quantitative Stem Cell Biology Laboratory, The Francis Crick Institute, 1 Midland Road, London NW1 1AT, UK.; ^6^ Stem Cells and Organoids Platform, The Francis Crick Institute, 1 Midland Road, London NW1 1AT, UK.; ^7^ Loke Centre for Trophoblast Research, Department of Physiology, Development and Neuroscience, University of Cambridge, Cambridge CB2 3EG, UK.; ^8^ Wellcome-MRC Cambridge Stem Cell Institute, University of Cambridge, Cambridge CB2 0AW, UK.; ^9^ Epigenetics Programme, Babraham Institute, Cambridge CB22 3AT, UK.; ^10^ Department of Infectious Disease, Faculty of Medicine, Imperial College London, London, UK.

## Abstract

The cis-regulatory potential of human endogenous retroviruses (HERVs) that have accumulated in our genome affects diverse host processes, including embryonic development and malignancy, where HERV epigenetic control is reduced. Non–small cell lung cancer optimal growth requires cancer cell–intrinsic expression of the calcium-binding protein calbindin under the control of a *HERVH* integrant on chromosome 8q21.3. Chimeric transcription of *HERVH* and *CALB1*, encoding calbindin, is also observed in human embryonic stem cells (ESCs), but its significance remained uncertain. Here, we show that *HERVH* integration on 8q21.3 during *Homininae* evolution coincides with acquisition of expression of *CALB1* in preimplantation embryos. We further show that *CALB1* expression is critical for human ESC growth and the prevention of cellular senescence and for the formation of ESC-derived preimplantation blastoids and postgastrulation amnioids. Thus, the co-option of *HERVH 8q21.3* cis-regulatory activity in lung cancer may echo its essential requirement for human embryo development.

## INTRODUCTION

Over the course of evolution, our genomes have amassed a considerable number of human endogenous retroviruses (HERVs) and related long-terminal repeat (LTR) retroelements, which greatly outnumber protein-coding and noncoding genes (*1*). These proviral DNA copies are derived from retroviral infection of the germ line during successive waves of retroviral epidemics. In addition to antiviral host defenses (*2*), epigenetic repression and accumulation of mutations or deletions mitigate the risk of reinfection and further amplification in the germ line from already integrated proviruses. All HERVs are thought to have been rendered replication-defective. Nevertheless, many proviruses have retained the ability to express retroviral proteins, as well as to act as cis-regulatory elements, affecting adjacent genes. HERVs that retain such features may still negatively affect host physiology or contribute to pathology (*3*–*5*). However, the genetic innovation they can provide may generate novel function for the host or become integrated into host developmental, homeostatic, and response processes.

The exaptation of HERV DNA regulatory sequences, RNA, and protein products may be particularly pronounced during early embryogenesis. Because of a combination of HERV adaptation and release from repression during epigenetic reprogramming, HERV expression is increased at these developmental stages, thus likely facilitating co-option (*6*, *7*). Differential responsiveness of phylogenetically distinct LTRs to the transcription factor combinations that characterize the progressive stages of human embryogenesis creates waves of stage-specific activation of the respective HERV families (*8*–*11*). Well-documented examples of HERV co-option in host development include the exaptation of envelope glycoproteins from distinct proviruses of the HERV-W and HERV-FRD families, giving rise to *Syncytin-1* (also known as *ERVW-1*) and *Syncytin-2* (also known as *ERVFRD-1*), respectively (*12*). The fusogenic potential of these envelope glycoproteins, which would normally mediate virus entry, is essential for the fusion of individual trophoblast cells during the formation of the syncytiotrophoblast (*12*). Collectively, the HERV-H family (referred to as HERVH hereafter), which comprises nearly 1000 proviruses and 1500 solitary LTRs (*9*), has been associated with the maintenance of human embryonic stem cell (ESC) pluripotency (*13*). Several mechanisms have been proposed to underlie this association, including *HERVH* proviral DNA copies acting as enhancers, alternative promoters of key genes as markers of topologically associating domain boundaries, or *HERVH* RNA transcripts acting as functional long noncoding RNAs, interacting with pluripotency transcription factors (*8*, *13**–**16*).

The evolutionary adaptability HERVs provide may also be exploited by transformed cells during tumor evolution, a process referred to as onco-exaptation (*17*, *18*). Aberrant activation of LTR elements can create tumor-promoting RNA isoforms, such as an oncogenic form of ALK (anaplastic lymphoma kinase) in melanoma (*19*), or drive ectopic expression of tumor-promoting proteins, such as CSF1R and IRF5 in Hodgkin’s lymphoma (*20*, *21*). We have previously described the cis-regulatory control of *CALB1* in non–small cell lung cancer by an upstream *HERVH* integration (*22*). *CALB1* encodes calbindin (also known as D28k), a calcium-binding protein, primarily expressed in healthy brain and kidney, where it protects from calcium toxicity and prevents cellular senescence (*23*, *24*). Consistent with its physiological function, the *HERVH-*driven calbindin isoform ectopically expressed in lung cancer cells promotes cancer cell–intrinsic growth by preventing cellular senescence (*22*), and similar observations have also been made in ovarian cancer cells (*25*).

*HERVH-CALB1* chimeric transcripts have also been detected in human ESCs and preimplantation embryos (*11*, *13*, *22*), but the consequences of this expression have not been investigated. Here, we examined the potential significance of *CALB1* transcriptional control by the adjacent *HERVH* provirus during human early embryogenesis. We show that expression of *CALB1* in the preimplantation embryo is a recently acquired phenotype in *Homininae* evolution, coinciding with the *HERVH* integration. Loss-of-function experiments further demonstrated an indispensable role for *CALB1* in ESC growth and prevention of senescence, as well as in their ability to generate embryonic and extraembryonic peri-implantation structures. Collectively, our findings highlight a critical function of *HERVH*-driven *CALB1* expression during human early embryo development, which may be exploited by cancer cells later in life.

## RESULTS

### *HERVH* drives *CALB1* expression during human embryo development

Given that *CALB1* is expressed in human ESCs and blastocysts (*13*, *22*), we first examined the balance of its isoforms. *HERVH-CALB1* chimeric transcripts have been detected in ESCs, and our prior analysis of single-cell RNA sequencing (RNA-seq) data from dissociated late blastocysts (*26*) identified *CALB1* transcription driven exclusively by the associated *HERVH* provirus (Fig. 1A) (*22*). However, similar analysis of RNA-seq data from initial outgrowth and early passages in primed ESC cultures (*26*) showed that *CALB1* transcription in these cells was instead driven by its own promoter despite concurrent transcription of the *HERVH* provirus (Fig. 1A). Comparable results were obtained by comparison of two- to -eight-cell stages or the inner cell mass (ICM) of preimplantation embryos (*27*), which expressed exclusively the *HERVH*-driven isoform, with long-term cultured ESCs, which expressed the canonical *CALB1* isoform (fig. S1A).

**Fig. 1.**
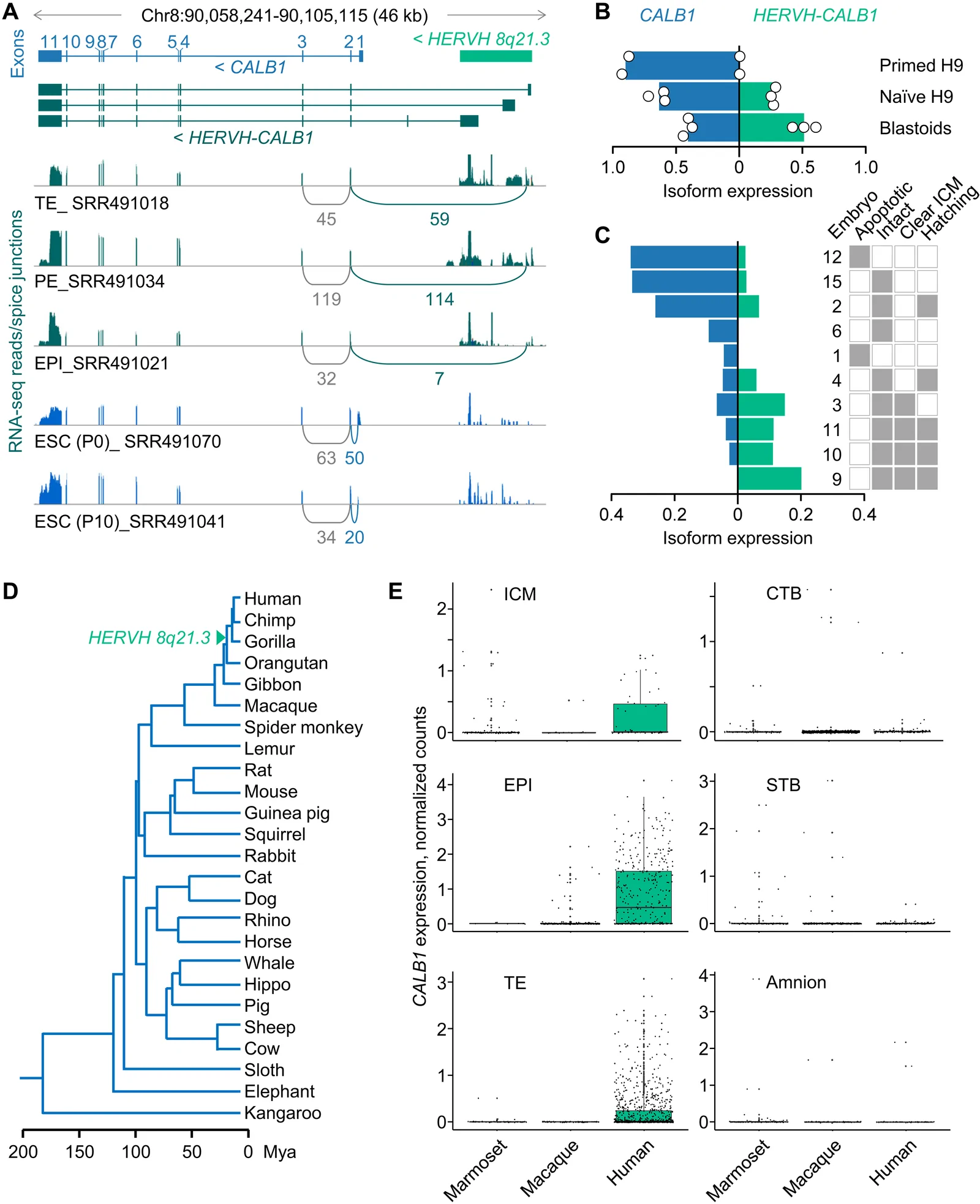
*HERVH* integration and control of early embryo *CALB1* expression. (**A**) *CALB1* locus and integrated *HERVH* provirus, with the GENCODE-annotated canonical *CALB1* and alternative *HERVH*-driven transcripts, and RNA-seq traces and numbers of splice junctions, determined by analysis single-cell RNA-seq data (GSE36552), in TE, PE, and EPI cells from human preimplantation embryos and passages 0 and 10 of ESC outgrowths (P0 and P10, respectively). (**B**) Canonical and *HERVH*-driven *CALB1* isoform expression, determined by isoform-specific RT-qPCR, in primed or naïve H9 cells or in blastoids derived from H9 cells. Symbols represent biological replicates. (**C**) Canonical and *HERVH*-driven *CALB1* isoform expression, determined by isoform-specific RT-qPCR, in blastocysts. Each bar represents an individual blastocyst. The blastocyst health and differentiation status are also indicated. (**D**) Estimation of the evolutionary age of the *HERVH 8q21.3* integration based on genomic sequence alignments. Mya, million years ago. (**E**) *CALB1* expression in the ICM, EPI, TE, cytotrophoblast (CTB), syncytiotrophoblast (STB), and amnion of the indicated species by analysis of publicly available data (*32*).

For orthogonal confirmation of promoter use, we assessed *CALB1* transcription in H9 human ESCs by isoform-specific reverse transcriptase–based quantitative polymerase chain reaction (RT-qPCR) assays (Fig. 1B). H9 ESCs, maintained at the primed state, expressed almost exclusively the canonical *CALB1* isoform (Fig. 1B). To examine whether this phenotype resulted from a more differentiated state of long-term H9 cultures than the ICM from which they derive, we tested H9 cells that were reverted to a naïve state (*28*), as well as a stem cell–based embryo model of the blastocyst (blastoids) generated from the latter (*29*, *30*). In contrast to their primed counterparts, naïve H9 expressed detectable levels of *HERVH*-driven *CALB1*, and the balance shifted further in favor of the *HERVH*-driven isoform in blastoids (Fig. 1B). Blastocysts developed from primary embryos exhibited a range of isoform balance, with expression of *HERVH-CALB1* isoform correlating with successful development (Fig. 1C and movies S1 to S10).

Thus, *HERVH* appears to be the exclusive promoter of *CALB1* in the early embryo in vivo and remains transcriptionally active in cultured ESCs, where *CALB1* is driven primarily by its own promoter, indicating an additional enhancer function for *HERVH*. Supporting the additional enhancer activity of the *HERVH 8q21.3* integration, CRISPR-Cas9 endonuclease–mediated deletion of this provirus in HARA lung cancer cells was previously shown to abolish the expression of *CALB1* driven by the *HERVH 8q21.3* provirus, as well as by the canonical promoter (*22*). In keeping with findings in HARA cells (*22*), CRISPR-Cas9 endonuclease–mediated deletion of the *HERVH 8q21.3* integration in naïve H9 ESCs also abolished the expression of *CALB1* driven by the *HERVH 8q21.3* provirus, as well as by the canonical promoter (fig. S1B), indicating an essential contribution of this provirus to *CALB1* regulation also in H9 ESCs.

Interrogation of functional enhancers in primed and naïve human ESCs (*31*) identified peaks of histone modifications and NANOG binding upstream of the *CALB1* gene, which mapped to the *HERVH* provirus (fig. S2A). Histone modifications, but not NANOG binding, additionally mapped to a further upstream *MER52C* LTR element integration (fig. S2A). However, this *MER52C* element did not exhibit evidence of transcription that may be associated with functional enhancer activity in preimplantation embryos or ESCs, and in contrast to the *HERVH* integration, it was also transcriptionally silent in cancer cell lines that express *HERVH-CALB1* (fig. S2, B and C). The lack of *MER52C* activity was characteristic of the entire *MER52A* and *MER52C* subfamilies, which were transcriptionally silent both in H9 ESCs and in primary squamous lung cancer samples, contrasting the strong transcriptional activity of the *HERVH 8q21.3* integration and of the *HERVH* subfamilies more broadly (fig. S2D).

To further probe a contribution of the *HERVH 8q21.3* integration to *CALB1* regulation, we examined a potential evolutionary link between the two. Comparative genomic analyses indicated that the *HERVH 8q21.3* integration was acquired after the split of the *Homininae* (human, chimpanzee and gorilla) and *Ponginae* (orangutan) subfamilies ∼13 million years ago (Fig. 1D). Consistent with a cis-regulatory role for the *HERVH 8q21.3* integration, comparison of human and lower primate embryos (*32*) revealed that *CALB1* was expressed specifically in the ICM, epiblast (EPI), and trophectoderm (TE) of human embryos but not those of marmosets or macaques (Fig. 1E). *CALB1* mRNA was detectable in murine oocytes, likely owing to maternal transcription, but was completely absent from early embryos of mice or guinea pigs (fig. S3). Therefore, expression of *CALB1* during embryo development is a characteristic of humans, associated with the relatively recent *HERVH 8q21.3* integration, and is not seen in earlier species lacking the *HERVH 8q21.3* integration.

Interrogation of a recent human embryo lineage annotation (*32*) showed the strongest *CALB1* expression in the EPI, ICM, and mesoderm, with weaker expression in the TE, hypoblast, zygote, two- to eight-cell, and prelineage stages (fig. S4). By immunofluorescence staining, H9 cells were found to express calbindin homogeneously, with cytoplasmic and perinuclear localization (Fig. 2A). In blastoids derived from H9 ESCs, calbindin staining was found in cells also expressing GATA3, marking the TE and, to a lesser extent, in SOX2-expressing cells, marking the EPI (Fig. 2B). Similarly, calbindin staining in primary blastocysts was colocalized with cells also expressing GATA3 or SOX2 (Fig. 2C), in agreement with the RNA expression data (fig. S4). Together, these findings demonstrated that calbindin is expressed in human ESCs, blastoids, and primary blastocysts under the control of the *HERVH 8q21.3* integration, raising the possibility that it may play a role in human embryo development.

**Fig. 2.**
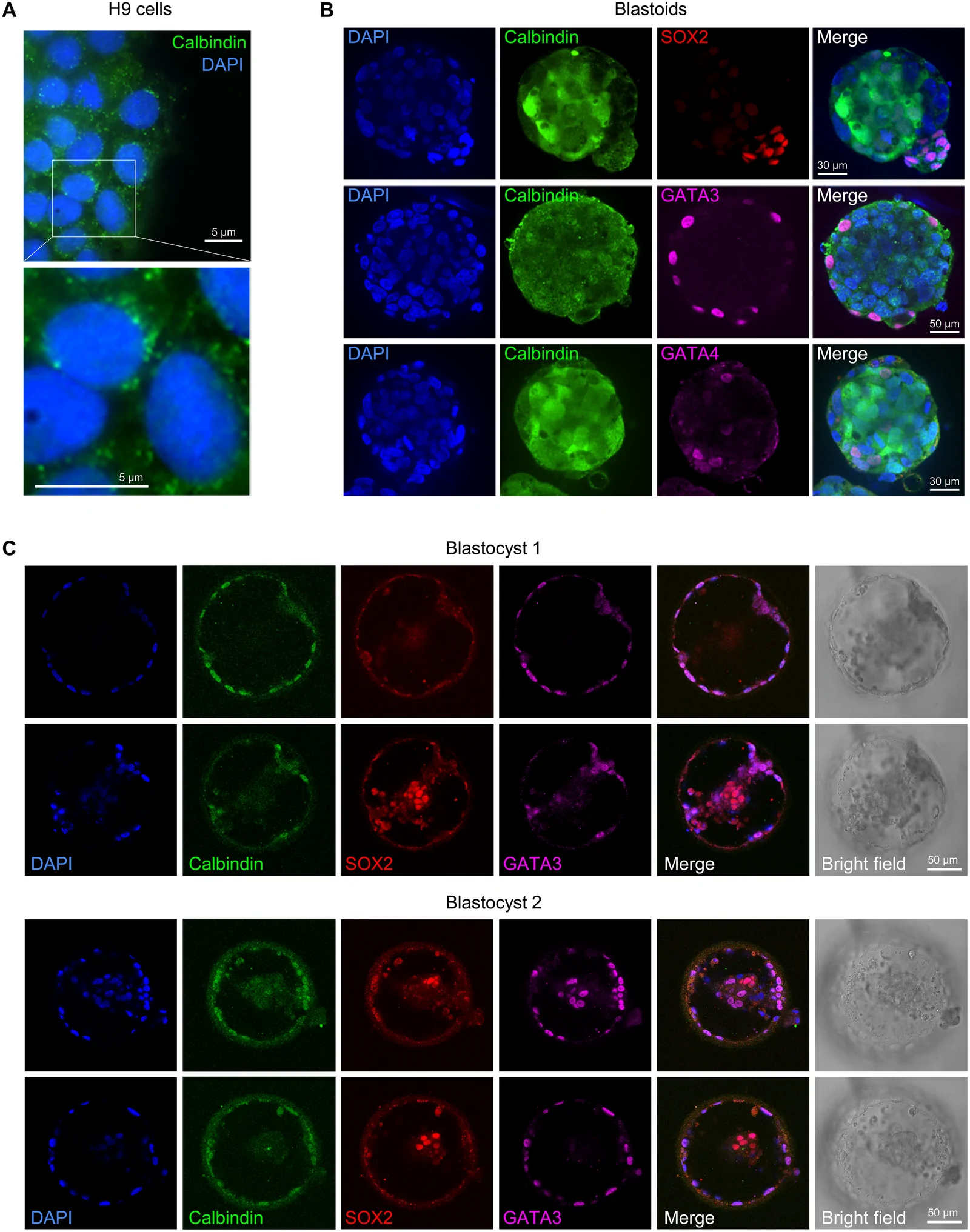
Calbindin expression in human ESCs and preimplantation embryos. (**A**) Immunofluorescence staining for calbindin and nuclear DNA staining with DAPI in primed H9 ESCs. (**B**) Immunofluorescence staining for calbindin and lineage markers SOX2, GATA3, and GATA4 and nuclear DNA staining with DAPI in blastoids formed by H9 ESCs. (**C**) Immunofluorescence staining for calbindin and lineage markers SOX2 and GATA3 and nuclear DNA staining with DAPI in two human blastocysts. Rows represent different images of the same blastocyst at different *z*-heights.

### Defective growth of calbindin-deficient human ESCs

To investigate the relevance of calbindin expression, we examined the effect of its loss in H9 ESCs. We used CRISPR-Cas9 endonuclease–mediated mutagenesis of exon 4, common to both isoforms (*22*). We established three separate clones of H9 cells (clones 4, 25, and 32) with *CALB1* mutations (*CALB1*^em^), as well as three *CALB1* wild-type (*CALB1*^wt^) control clones that underwent the same procedure (clones 8, 23, and 31). *CALB1*^em^ clones carried distinct genomic DNA mutations in exon 4 (fig. S5) and exhibited normal karyotypes (fig. S6). Assessed by Western blotting, *CALB1*^em^, but not control clones, lost calbindin expression, although residual expression could still be detected in clone 4 (Fig. 3A).

**Fig. 3.**
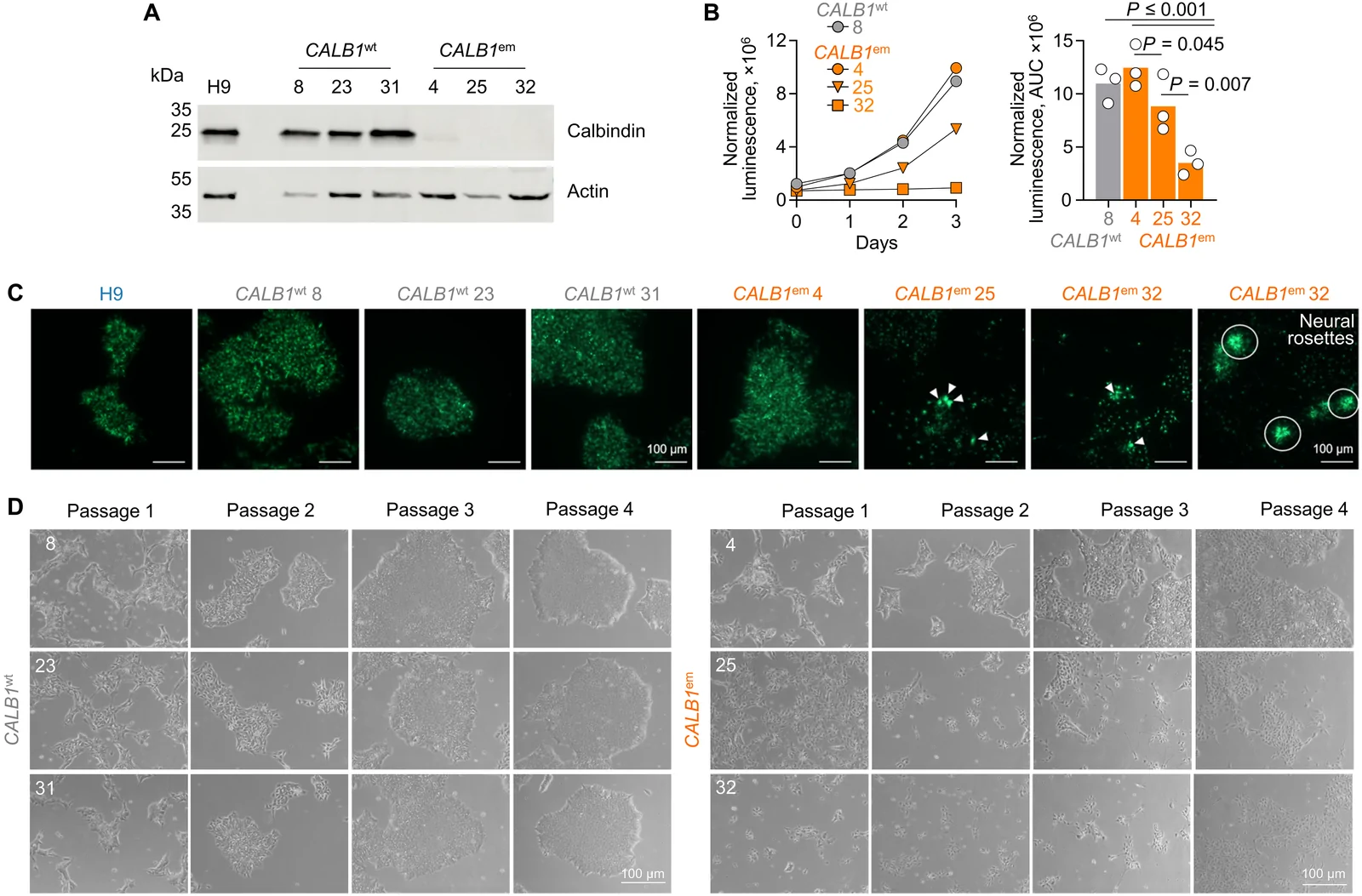
Growth defects of CRISPR-Cas9–targeted *CALB1*^em^ ESC clones. (**A**) Western blot of calbindin protein in lysates from parental H9 cells and in *CALB1*^wt^ and *CALB1*^em^ clones. One of two experiments is shown. (**B**) Normalized CellTiterGlo luminescence accumulation during growth of *CALB1*^wt^ clone 8 and *CALB1*^em^ clones 4, 25, and 32 (left) and quantitation of area under the curve (AUC) luminescence values. Symbols represent individual experiments, and *P* values were calculated using one-way repeated-measures ANOVA tests, with Bonferroni correction for multiple comparisons. (**C**) Staining for senescence-associated β-galactosidase in parental H9 cells and in *CALB1*^wt^ and *CALB1*^em^ clones. Arrows indicate senescent cells. (**D**) Bright-field images depicting the morphology of *CALB1*^wt^ and *CALB1*^em^ clones over successive passages.

Notably, *CALB1*^em^ clones 25 and 32 exhibited a significant growth defect in standard culture conditions, whereas *CALB1*^em^ clone 4 grew comparably with a representative of the *CALB1*^wt^ clones, clone 8 (Fig. 3B). Consistent with these growth rates and also with the established role of calbindin in preventing senescence (*22**–**25*), *CALB1*^em^ clones 25 and 32 showed increased β-galactosidase activity, characteristic of cellular senescence, which was absent from *CALB1*^em^ clone 4 or from *CALB1*^wt^ clones and parental H9 cells (Fig. 3C). In addition, *CALB1*^em^ clone 32, which exhibited the slowest growth in these sets of experiments, also showed evidence for formation of β-galactosidase–positive rosettes (Fig. 3C), indicating spontaneous neural differentiation. Whereas colonies of *CALB1*^wt^ control clones exhibited a compact morphology with tightly packed cells and well-defined edges, characteristic of the undifferentiated state, those formed by *CALB1*^em^ clones, particularly clones 25 and 32, were smaller and consisted mostly of loosely packed cells with elongated shapes (Fig. 3D), again suggestive of spontaneous differentiation. In agreement with looser colonies of more differentiated cells, *CALB1*^em^ clones 25 and 32 lacked the expression of Connexin 43, which is abundantly expressed in parental H9 cells and in *CALB1*^em^ clone 4 (fig. S7).

Cultures of *CALB1*^em^ and *CALB1*^wt^ control clones were established from cryopreserved cells an additional six times, and *CALB1*^em^ clones 25 and 32 exhibited growth defects in all runs, whereas *CALB1*^em^ clone 4 was consistently unaffected. However, the severity of the growth phenotype of *CALB1*^em^ clones 25 and 32 varied by experimental run between a partial defect and nearly complete senescence, likely as a result of culture conditions, with clone 32 exhibiting the most severe defects in some runs and clone 25 in other runs, when compared to one another, while both being defective compared with control clones. Regardless of growth rate, however, *CALB1*^em^ clone 25 and 32 colony morphology was invariably affected in all runs.

For a more comprehensive comparison, we transcriptionally profiled *CALB1*^em^ and *CALB1*^wt^ control clones by RNA-seq. We first inspected the transcription of the *CALB1* locus and associated *HERVH 8q21.3* provirus (Fig. 4A) to assess the degree of *CALB1* deficiency. This analysis revealed an 87 and 90% reduction in *CALB1* transcription in *CALB1*^em^ clones 25 and 32, respectively, when compared with parental H9 cells, whereas *CALB1*^em^ clone 4 had lost only 45% (Fig. 4B). In contrast, *HERVH 8q21.3* transcription was not appreciably affected in any of the clones (Fig. 4B). The residual expression of *CALB1* in all three *CALB1*^em^ clones included an alternative isoform that skipped the canonical exon 4 (Fig. 4A). Reads spanning the splice junction between exons 3 and 4 comprised the vast majority in parental H9 cells and all *CALB1*^wt^ clones but were absent from all *CALB1*^em^ clones, where reads spanning predominantly the alternative splice junction between exons 3 and 5 were found instead (Fig. 4C).

**Fig. 4.**
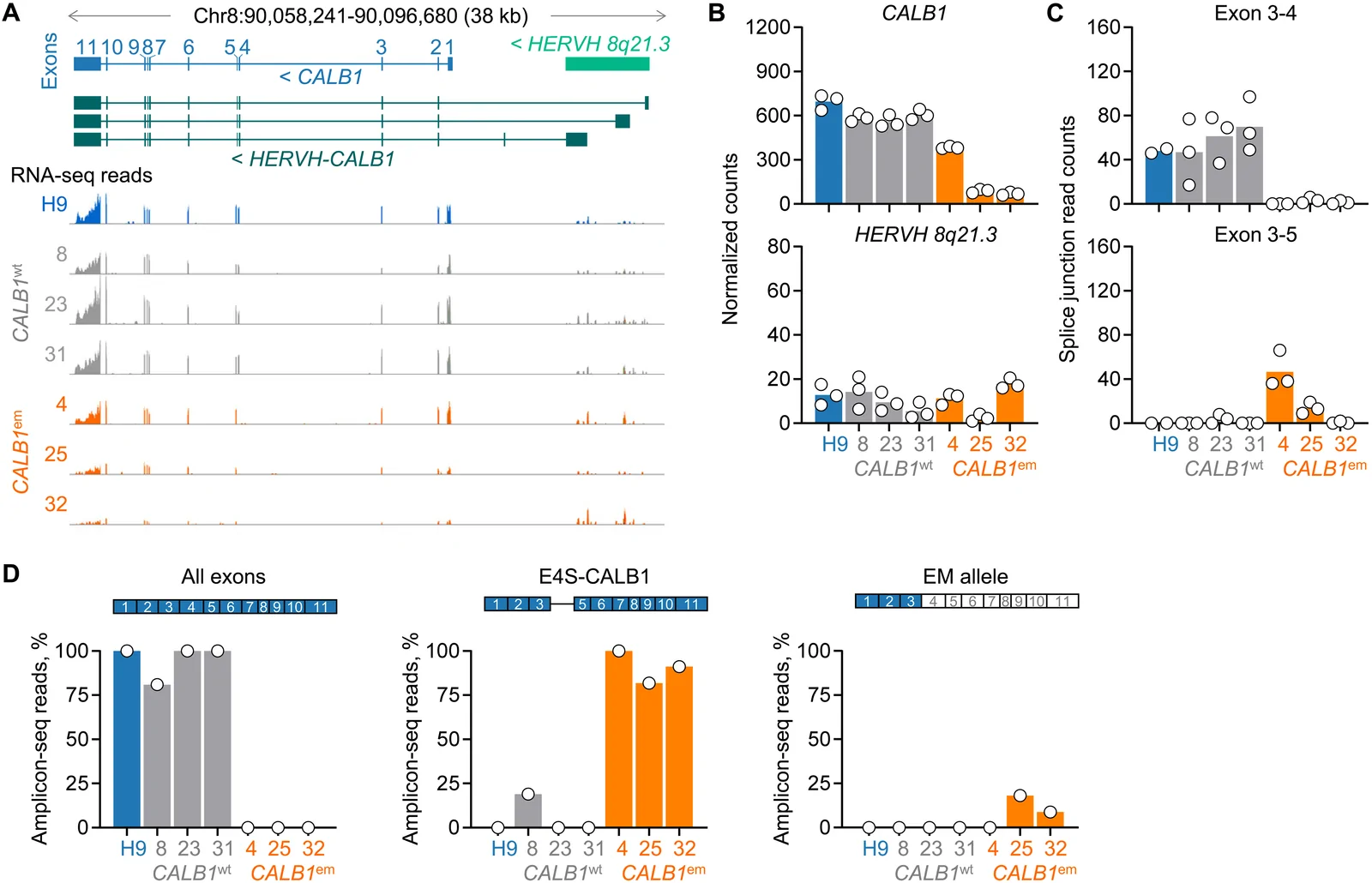
*CALB1* levels and isoform expression in *CALB1*^em^ ESC clones. (**A**) *CALB1* locus and integrated *HERVH* provirus and RNA-seq traces from parental H9 cells and from *CALB1*^wt^ and *CALB1*^em^ clones. (**B**) Quantitation of RNA-seq read counts for the *CALB1* gene (top) and the *HERVH 8q21.3* provirus (bottom) in parental H9 cells and from *CALB1*^wt^ and *CALB1*^em^ clones. Symbols represent technical RNA-seq replicates. (**C**) Quantitation of RNA-seq reads spanning the junctions of *CALB1* exon 3-4 (top) and 3-5 (bottom) in parental H9 cells and from *CALB1*^wt^ and *CALB1*^em^ clones. Symbols represent technical RNA-seq replicates. (**D**) The proportion of reads, assessed by deep sequencing of full-length cDNA amplified from parental H9 cells and from *CALB1*^wt^ and *CALB1*^em^ clones, that correspond to the canonical message included all exons (left), the exon 4–skipping (E4S-CALB1) isoform (middle), or the respective endonuclease-mediated mutants (EM allele) (right).

For additional validation and to probe the protein-coding potential of *CALB1* transcripts in *CALB1*^em^ clones, we amplified full *CALB1* cDNAs, initiated by either *HERVH 8q21.3* or the canonical promoter, and subjected them to deep sequencing. Translation of sequenced amplicons identified several distinct calbindin isoforms (fig. S8A). In addition to the canonical and N-terminally truncated, *HERVH*-driven calbindin (HERVH-CALB1), we also identified the exon 4–skipping isoform (E4S-CALB1), indicated by RNA-seq analysis (fig. S8A). In this isoform, exon 5 and subsequent exons remain in frame with exon 3, resulting in the loss of 28 amino acids between the second EF-hand motif, which does not contain a calcium-binding site, and the third EF-hand motif of the full-length protein (fig. S8A). Consequently, all four calcium-binding sites are preserved in the E4S-CALB1 isoform (fig. S8B). At the protein level, canonical calbindin and HERVH-CALB1 calbindin were previously found to have comparable activity in rescuing the growth defects caused by CRISPR-Cas9 endonuclease–mediated loss of calbindin function in HARA lung cancer cells (*22*), and although the functional activity of the E4S-CALB1 isoform was not tested, structural considerations suggest that it is likely retained, even if reduced. As expected, *CALB1*^em^ clones also expressed isoforms with premature stop codons in the targeted exon 4, encoding substantially C-terminally truncated proteins (fig. S8A) and matching the genotype of the respective clone (fig. S5). In addition to the substantial truncation of protein products, these premature stop codons would render the transcripts subject to nonsense-mediated decay, and we therefore considered them as null isoforms.

As a fraction, the exon 4–retaining isoform was the dominant isoform (∼94%) in parental H9 cells and *CALB1*^wt^ control clones (Fig. 4D). Nevertheless, the E4S-CALB1 isoform was also detected at a lower frequency (∼6%) in these cells (Fig. 4D), indicating that this previously unannotated isoform is a part of the physiological transcriptome of human ESCs. *CALB1*^em^ clones expressed predominantly the E4S-CALB1 isoform, and *CALB1*^em^ clones 25 and 32 also expressed lower levels of mutated exon 4–retaining isoforms (Fig. 4D). Collectively, these analyses indicated that the *CALB1* alleles in *CALB1*^em^ clones were hypomorphic, rather than complete knockout alleles. The residual expression in these clones may further indicate selection pressure to maintain a minimum level of *CALB1* activity in H9 ESCs. This notion is in agreement with the phenotypic differences between *CALB1*^em^ clones, with clone 4 exhibiting a subtler phenotype and retaining 56% of *CALB1* transcription, predominantly of E4S-CALB1 isoforms, and clones 25 and 32 exhibiting a more severe phenotype and retaining <13% of *CALB1* transcription.

To independently verify that the growth patterns observed in *CALB1*^em^ clones were the result of the corresponding reduction in *CALB1* expression, as opposed to inherent variability introduced during the cloning step, we took an orthogonal approach of depleting *CALB1* without prolonged culture or genetic bottlenecks. To this end, we acutely reduced *CALB1* mRNA expression in H9 ESCs using *CALB1*-targeting small interfering RNAs (siRNAs) (fig. S9). While H9 cells transfected with a pool of nontargeting control siRNAs were 91 to 97% positive for calbindin, transfection with individual *CALB1*-targeting siRNAs caused a significant reduction in calbindin expression down to 34 to 50% and 9 to 22% positivity at 48 and 72 hours posttransfection, respectively (fig. S9). Cell numbers at these time points were also significantly reduced by transfection with individual *CALB1*-targeting siRNAs, in proportion with the *CALB1* knockdown efficiency (fig. S9). Accompanying the reduction in recovered cell numbers, siRNA-mediated *CALB1* knockdown also altered the proportion of cells at distinct phases of the cell cycle. In H9 cells transfected with control siRNAs, calbindin^+^ cells, which were an overwhelming majority, were enriched for cells synthesizing DNA, detected by 5-ethynyl-2′-deoxyuridine (EdU) incorporation (S-phase), and depleted for cells in mitosis, characterized by condensed chromosomes and phospho-histone H3 (pH3) positivity (M-phase), when compared with the minority of calbindin^−^ cells (fig. S9), suggestive of *CALB1* regulation during the cell cycle. Notably, in H9 cells transfected with *CALB1*-targeting siRNAs, the balance of M-phase and S-phase cells was shifted further away from that seen in control cells, with significant changes in the proportion of EdU^+^ and pH3^+^ cells within the targeted calbindin^−^ fraction, compared with calbindin^+^ cells in the same cultures (fig. S9). Thus, acute siRNA-mediated depletion of *CALB1* at the population level phenocopied the growth defects observed in *CALB1*^em^ clones and further indicated defective progression through the cell cycle.

### Calbindin deficiency triggers differentiation

Having determined the degree of *CALB1* deficiency in *CALB1*^em^ clones, we then assessed the corresponding changes in gene expression compared with *CALB1*-fully sufficient clones (Fig. 5A). We selected genes that were differentially expressed (≥2-fold, *P* < 0.05) when all *CALB1*^em^ clones were compared with H9 cells and all *CALB1*^wt^ control clones and applied a 5% false discovery rate (*q* < 0.05), which resulted in 94 genes (data file S1). Unsupervised hierarchical clustering based on the 94 genes effectively separated *CALB1*^em^ clones 25 and 32 from the control clones, whereas *CALB1*^em^ clone 4 exhibited an intermediate transcriptional signature (Fig. 5A), in line with the residual expression of *CALB1*. This set of genes shared among the *CALB1*^em^ clones was primarily responsible for their separation from the control clones (Fig. 5B), and pathway analyses highlighted development and differentiation processes as the most affected pathways (Fig. 5C). Accordingly, *CALB1*^em^ clones 25 and 32, and to a lesser extent, clone 4, acquired the expression of the neuronal marker microtubule-associated protein 2 (MAP2), as well as neuronal morphology (Fig. 5D).

**Fig. 5.**
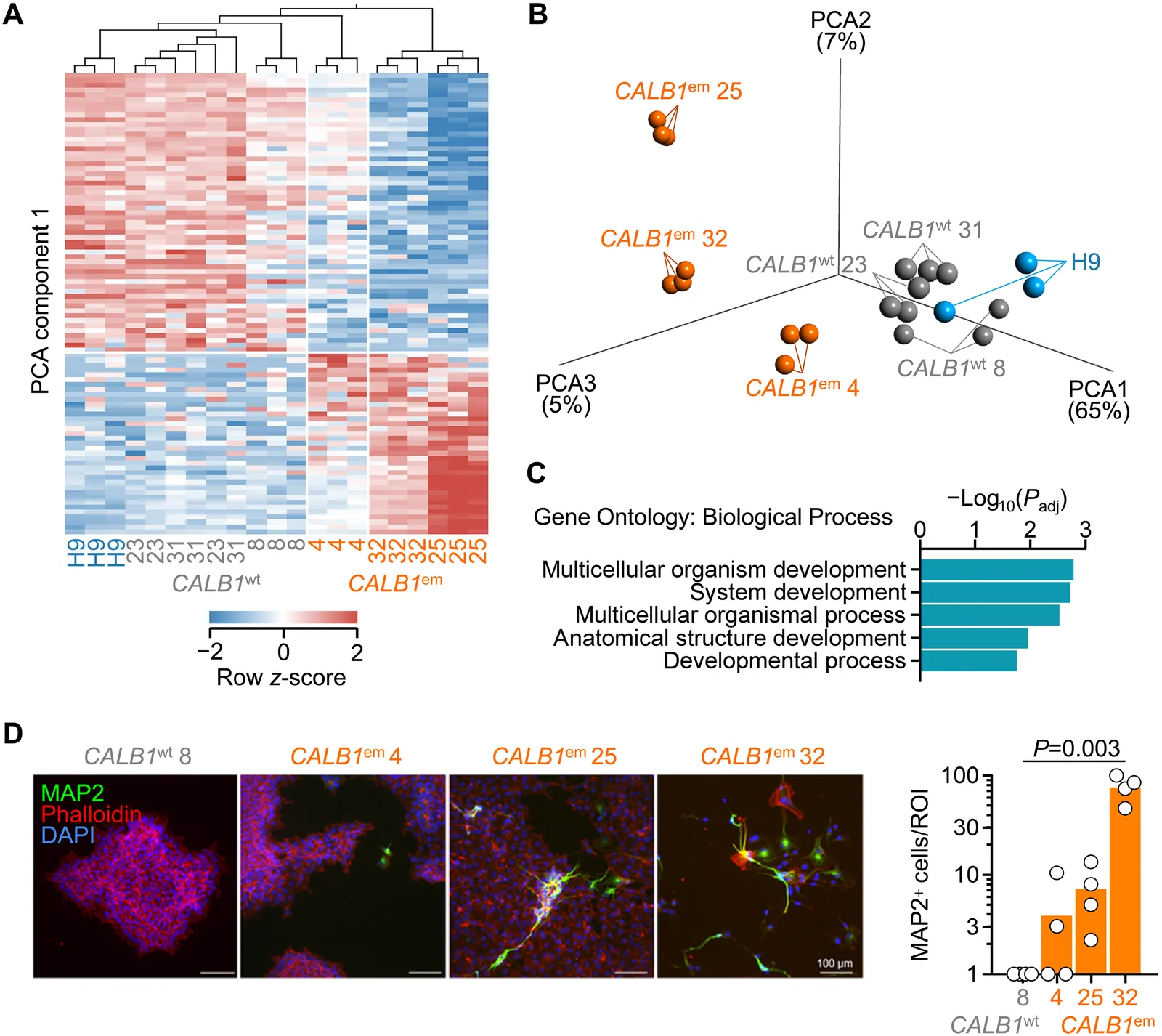
Transcriptional dysregulation in *CALB1*^em^ ESC clones. (**A**) Heatmap of 94 differentially expressed genes (≥2-fold, *P* < 0.05, *q* < 0.05) between parental H9 cells and *CALB1*^wt^ clones (grouped together) and *CALB1*^em^ clones (grouped together), assessed by RNA-seq. Columns represent RNA-seq technical replicates. Samples are ordered by unsupervised hierarchical clustering, and genes are ordered by principal components analysis (PCA) component 1. (**B**) Proportion of variance between parental H9 cells and *CALB1*^wt^ and *CALB1*^em^ clones, accounted for by the first three PCA components on the basis of the 94 differentially expressed genes in (A). (**C**) Functional annotation by gene ontology (GO) (Biological Process) of the differentially expressed genes in *CALB1*^em^ clones. *P* values were calculated with the g:SCS algorithm. (**D**) Immunofluorescence staining for the neuronal marker MAP2 and filamentous actin with phalloidin and nuclear DNA staining with DAPI in *CALB1*^wt^ clone 8 and *CALB1*^em^ clones 4, 25, and 32 (left) and quantitation of MAP2-positive cells. Symbols represent randomly selected regions of interest (ROIs). *P* values were calculated with a Kruskal-Wallis one-way ANOVA on ranks, with Dunn’s correction for multiple comparisons.

The variability in the extent to which *CALB1*^em^ clones 25 and 32 were affected between runs suggested selection pressure to maintain basal *CALB1* expression, which in turn could lead to cellular heterogeneity within each clone. To investigate intraclonal heterogeneity, we expanded the three *CALB1*^em^ clones and *CALB1*^wt^ control clone 8 once again and compared their transcriptional profiles at the single-cell level. The growth of *CALB1*^em^ clones 25 and 32 was comparably affected in this run, whereas the growth of *CALB1*^em^ clone 4 was unaffected, which was reflected by their effective segregation at the single-cell transcriptome level according to genotype (Fig. 6A). UMAP (uniform manifold approximation and projection) dimensionality reduction showed that *CALB1*^wt^ clone 8 cells formed a cluster group (group 1), which minimally overlapped with cluster groups formed by *CALB1*^em^ cells (groups 2 and 3) (Fig. 6A). Group 2 was composed of cells from all three *CALB1*^em^ clones, albeit cells from *CALB1*^em^ clone 4 clustered separately within this group, whereas group 3 was composed exclusively of cells from *CALB1*^em^ clones 25 and 32, which were also interspersed in certain group 2 clusters (Fig. 6A). By pathway analyses of their distinguishing features, groups 1 and 2 expressed genes involved in stem cell maintenance and pluripotency, whereas group 3 expressed genes involved in cellular differentiation (Fig. 6, B and C). Consistent with bulk RNA-seq (Fig. 4B), *CALB1*^em^ clone 4 exhibited considerable *CALB1* expression at the clonal level, whereas *CALB1* expression was substantially reduced in *CALB1*^em^ clones 25 and 32 (Fig. 6D). Notably, residual *CALB1* expression in all *CALB1*^em^ clones was observed only in group 2 cells, which also expressed pluripotency genes including *NANOG*, *POU5F1*, and *PRDM14* (Fig. 6E). In contrast, *CALB1* expression was absent from group 3 cells, particularly in clusters 6, 13, and 14, which instead expressed lineage differentiation genes including *NR2F2*, *PAX6*, *PAX8*, and *VCAM1* (Fig. 6E). Thus, all *CALB1*^em^ clone 4 cells and a fraction of cells from *CALB1*^em^ clones 25 and 32 retained a certain level of stem cell maintenance and pluripotency potential, directly proportionally with the levels of *CALB1* expression, which were lost exclusively in the remaining fraction of *CALB1*^em^ clone 25 and 32 cells.

**Fig. 6.**
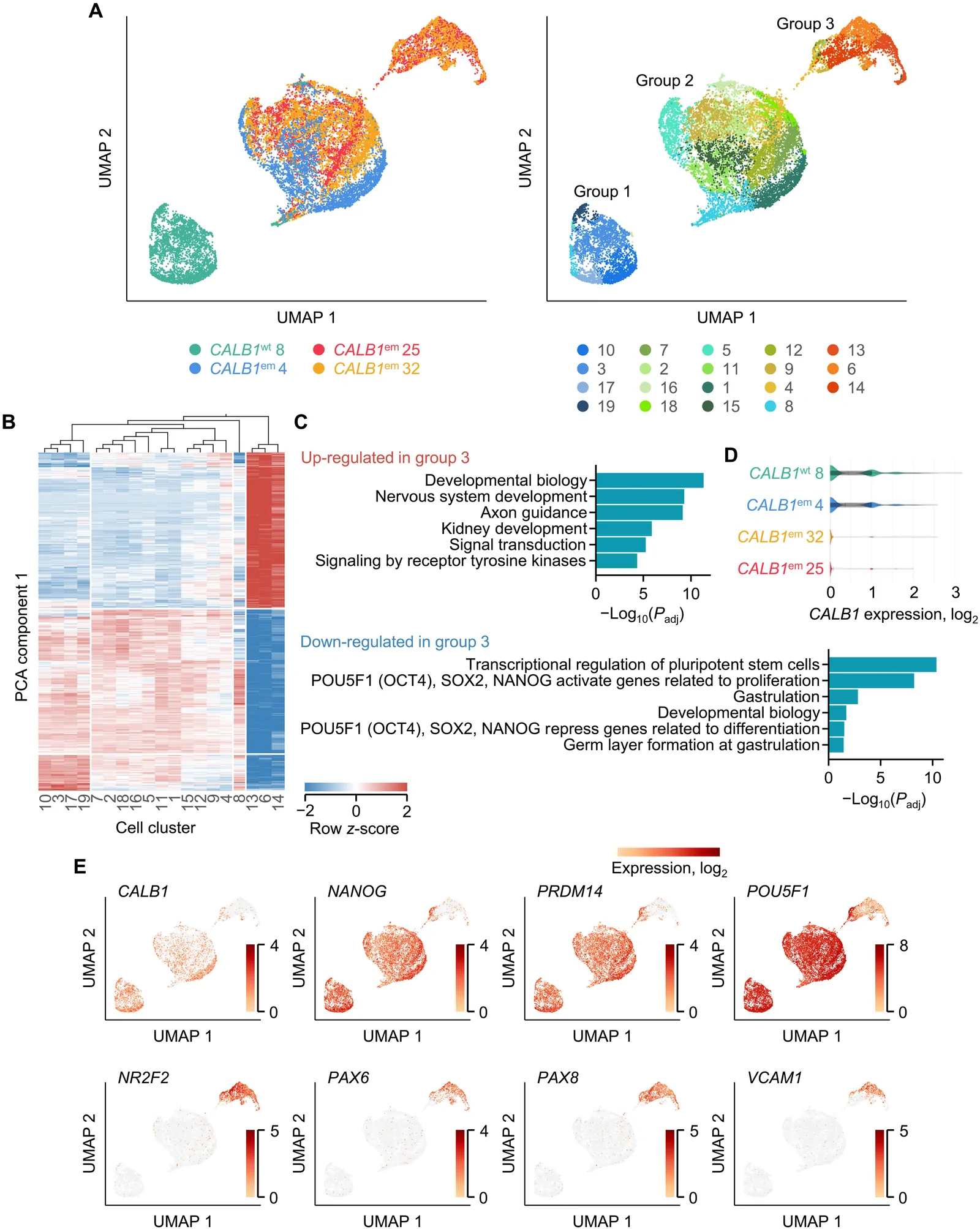
Transcriptional dysregulation in *CALB1*^em^ ESC single cells. (**A**) UMAP clustering of individual cells from *CALB1*^wt^ clone 8 and *CALB1*^em^ clones 4, 25, and 32 labeled by their genotype (left) or by their assigned cluster and cluster group (right) based on the transcriptional profiles from single-cell RNA-seq. (**B**) Heatmap of genes distinguishing the cluster groups in (A). Samples are ordered by unsupervised hierarchical clustering, and genes are ordered by PCA component 1. (**C**) Functional annotation by GO (Reactome) of the up-regulated or top 100 down-regulated genes in group 3 compared with the other two groups. *P* values were calculated with the g:SCS algorithm. (**D**) Violin plots of *CALB1* expression in individual cells from *CALB1*^wt^ clone 8 and *CALB1*^em^ clones 4, 25, and 32. (**E**) Expression of the indicated genes in UMAP cell cluster projections as in (A).

These results indicated that distinct levels of residual *CALB1* expression generated cellular heterogeneity in *CALB1*^em^ clones and further supported the idea that calbindin deficiency compromises ESC growth and instigates spontaneous differentiation. However, as the growth of *CALB1*^em^ clones even with the lowest level of calbindin expression was not fully abrogated and spontaneous differentiation was not complete at the population level, it was still possible that some degree of pluripotency was still retained, particularly by the minority of cells in *CALB1*^em^ clones 25 and 32 that retain residual *CALB1* expression. To examine the contribution of calbindin and, by extension, of the *HERVH 8q21.3* provirus to the maintenance of ESC pluripotency, we subjected them to conditions that induce differentiation into the three primary germ layers (fig. S10). Despite the growth defects of *CALB1*^em^ clones 25 and 32, all *CALB1*^em^ clones exhibited efficient endodermal, mesodermal, and ectodermal differentiation, comparable with that of the *CALB1*^wt^ control clone 8 under these conditions (fig. S10).

As a further test, we examined the ability of *CALB1*^em^ clones to adopt a naïve state of pluripotency. Upon chemical primed-to-naïve cell resetting (*28*), *CALB1*^em^ clones did form naïve cell colonies (Fig. 7A) and maintained the normal karyotype (fig. S11). However, the efficiency of naïve cell colony formation was significantly reduced specifically for *CALB1*^em^ clones 25 and 32 (Fig. 7A). Acquisition of the naïve state markers CD75 and SUSD2 (Sushi domain–containing protein 2) was as efficient in the naïve cell colonies formed by all *CALB1*^em^ clones, including clones 25 and 32, as those formed by the *CALB1*^wt^ control clone 8 (fig. S12A), and was absent from primed cells, suggesting successful conversion to a naïve-like state in all clones. Moreover, when assessed by RNA-seq, *CALB1*^em^ clones expressed comparable low levels of lineage differentiation genes *FOXA2*, *PAX6*, *SOX17*, *TBXT*, *MIXL1*, and *NES* and high levels of pluripotency genes *DPPA4*, *SOX2*, *POU5F1*, *PRDM14*, and *NANOG*, with the control *CALB1*^wt^ control clone 8 at the naïve-like state, despite up-regulation of some differentiation genes and down-regulation of pluripotency genes at the primed state (fig. S12B).

**Fig. 7.**
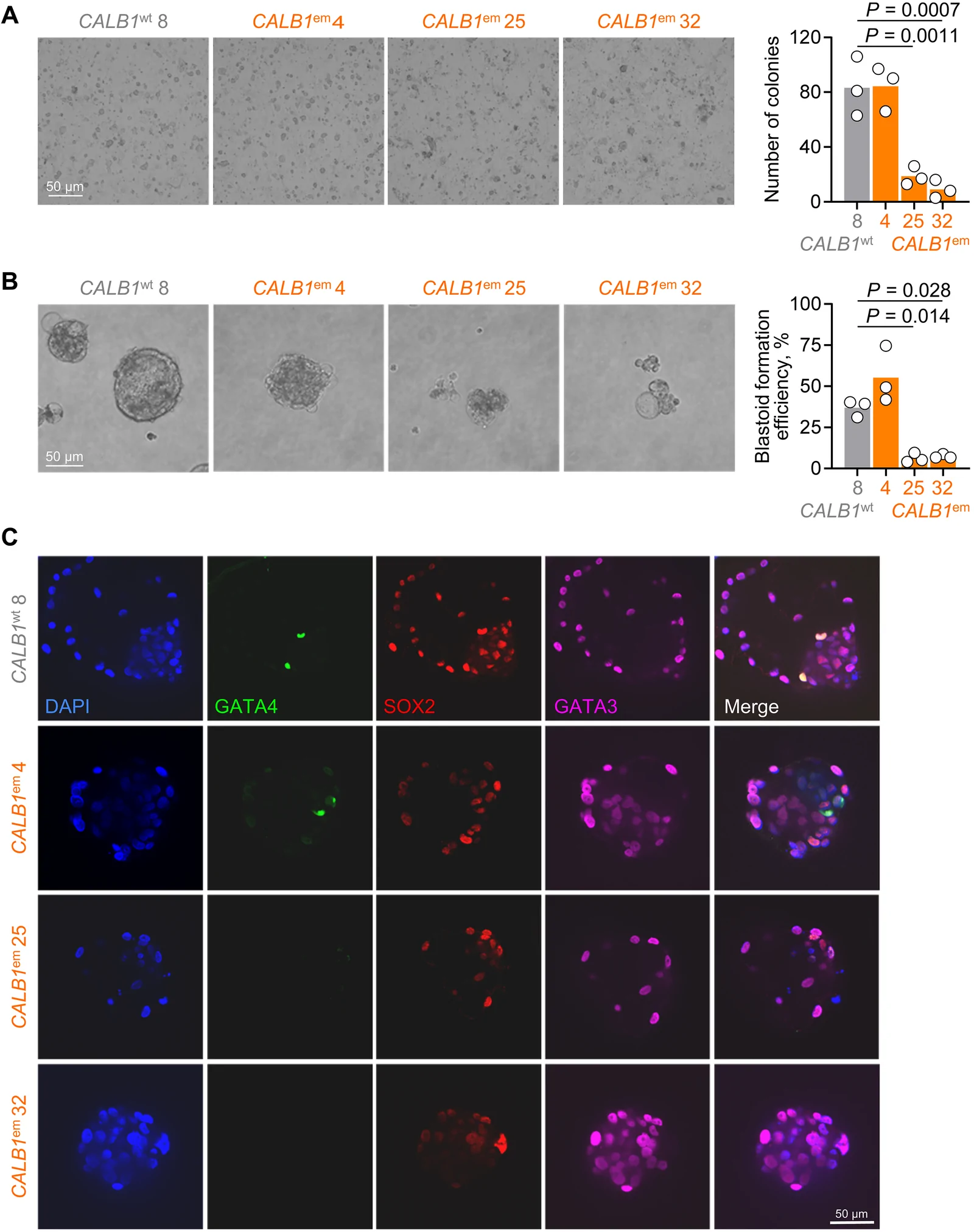
Defective blastoid development of *CALB1*^em^ ESC clones. (**A**) Morphology (left) and quantitation (right) of naïve ESC colonies formed by chemically reset naïve *CALB1*^wt^ clone 8 and *CALB1*^em^ clones 4, 25, and 32. Symbols represent technical replicates. *P* values were calculated with a one-way ANOVA with Holm-Sidak correction for multiple comparisons. (**B**) Representative morphology (left) and quantitation (right) of blastoids formed by *CALB1*^wt^ clone 8 and *CALB1*^em^ clones 4, 25, and 32. Symbols represent independent replicates. *P* values were calculated with one-way repeated-measures ANOVA tests, with Dunnett’s correction for multiple comparisons. (**C**) Immunofluorescence staining for lineage markers SOX2, GATA3, and GATA4 and nuclear DNA staining with DAPI in blastoids formed by *CALB1*^wt^ clone 8 and *CALB1*^em^ clones 4, 25, and 32.

Notably, primed-to-naïve cell resetting was accompanied with very strong induction (>150-fold) of *HERVH 8q21.3* transcription in all clones and with a switch in favor of the *HERVH*-driven *CALB1* transcription (fig. S13, A and B), in agreement with the isoform-specific RT-qPCR assays (Fig. 1B). In turn, this was associated with elevated overall expression of *CALB1*, particularly in the two *CALB1*^em^ clones examined (4 and 32), where it reached ∼72% of normal levels seen in *CALB1*^wt^ control clone 8 (fig. S13, A and B). In addition to increased overall levels, *HERVH*-driven *CALB1* transcription specifically in *CALB1*^em^ clones resulted almost exclusively in the *E4S-CALB1* mRNA isoform, which produces the only possible calbindin isoform in these cells (fig. S13, A and C). This compensatory increase indicated selection for calbindin expression during primed-to-naïve cell conversion and blunted transcriptional differences between the two *CALB1*^em^ clones and the *CALB1*^wt^ control clone 8 (fig. S13D). Nevertheless, when considered together, *CALB1*^em^ clones 4 and 32 were still transcriptionally distinct from the *CALB1*^wt^ control clone 8 in unsupervised hierarchical clustering (fig. S13D), and when considered separately, *CALB1*^em^ clone 32 exhibited further distance from either *CALB1*^em^ clone 4 or *CALB1*^wt^ clone 8, with dysregulation of genes involved in developmental pathways and neuronal development in particular (fig. S13, E and F, and data file S2).

Therefore, the significant reduction in *CALB1* expression severely compromised the growth of primed *CALB1*^em^ clones 25 and 32, as well as the efficiency with which they formed naïve cell colonies upon chemical resetting. However, retention of residual *CALB1* expression in a minority of cells in *CALB1*^em^ clones 25 and 32 appeared sufficient to preserve their pluripotency potential and the ability to acquire a naïve-like cell phenotype.

### *CALB1* is essential for the formation of embryonic and extraembryonic structures

The marked expression of *CALB1* in the EPI, ICM, mesoderm, TE, and hypoblast suggested that it may be involved in the formation of later-stage embryonic and extraembryonic structures derived from these layers. To investigate whether *CALB1* expression was required for distinct stages of human embryo development, we generated blastoids from naïve *CALB1*^em^ clones (*29*, *30*). Compared with that of *CALB1*^wt^ control clone 8, the efficiency of blastoid formation by *CALB1*^em^ clones 25 and 32 was severely impaired, whereas *CALB1*^em^ clone 4 did not exhibit a defect (Fig. 7B), suggesting a critical requirement for calbindin in this process. The few blastoids that did form by *CALB1*^em^ clones 25 and 32 resembled control blastoids and expressed the ICM and TE markers SOX2 and GATA3, respectively, but lacked the expression of the primitive endoderm (PE) marker GATA4 (Fig. 7C), suggesting defective EPI and PE segregation.

To examine the potential involvement of *CALB1* expression in the development of extraembryonic structures, we used a recently developed model of ESC-derived postgastrulation amnioids (PGAs) (*33*). We first modified the protocol, originally optimized for H1 human ESCs (*33*), to improve the efficiency of PGA formation by H9 human ESCs (Fig. 8A and fig. S14). In stark contrast to *CALB1*^wt^ control clone 8 and *CALB1*^em^ clone 4, *CALB1*^em^ clones 25 and 32 exhibited a complete failure to form an amniotic-like structure from PGAs, and instead formed compact structures (Fig. 8A and fig. S14). To further investigate the defect in PGA formation by *CALB1*^em^ clones, we examined the efficiency of amniotic ectoderm and extraembryonic mesoderm differentiation on day 2 of the two-dimensional (2D) cultures of the PGA formation protocol. Colonies of *CALB1*^wt^ control clone 8 and *CALB1*^em^ clone 4 cells exhibited the expected pattern of central expression of the extraembryonic mesoderm marker GATA6, surrounded by cells expressing the amniotic ectoderm marker TFAP2C (transcription factor AP-2γ) (Fig. 8B). In contrast, GATA6 was expressed throughout colonies of *CALB1*^em^ clones 25 and 32, with TFAP2C-expressing cells forming tight clusters interspersed with GATA6-expressing cells (Fig. 8B), suggesting irregular morphological patterning and localization of the two lineages. Accompanying defective embryonic mesoderm and amniotic ectoderm separation, and in contrast to *CALB1*^wt^ clone 8 and *CALB1*^em^ clone 4 colonies, *CALB1*^em^ clone 25 and 32 colonies also expressed OTX2 (orthodenticle homeobox 2) and PAX6 (paired box 6), associated with neuroectoderm differentiation (fig. S15). Moreover, OTX2 and PAX6 expression was observed in clusters of cells that were distinct from the TFAP2C-expressing clusters (fig. S15). Together, these results indicated a shift in the balance of amniotic ectoderm and neuroectoderm differentiation and a complete subsequent block in PGA formation in *CALB1*^em^ clones 25 and 32 with the lowest residual calbindin expression.

**Fig. 8.**
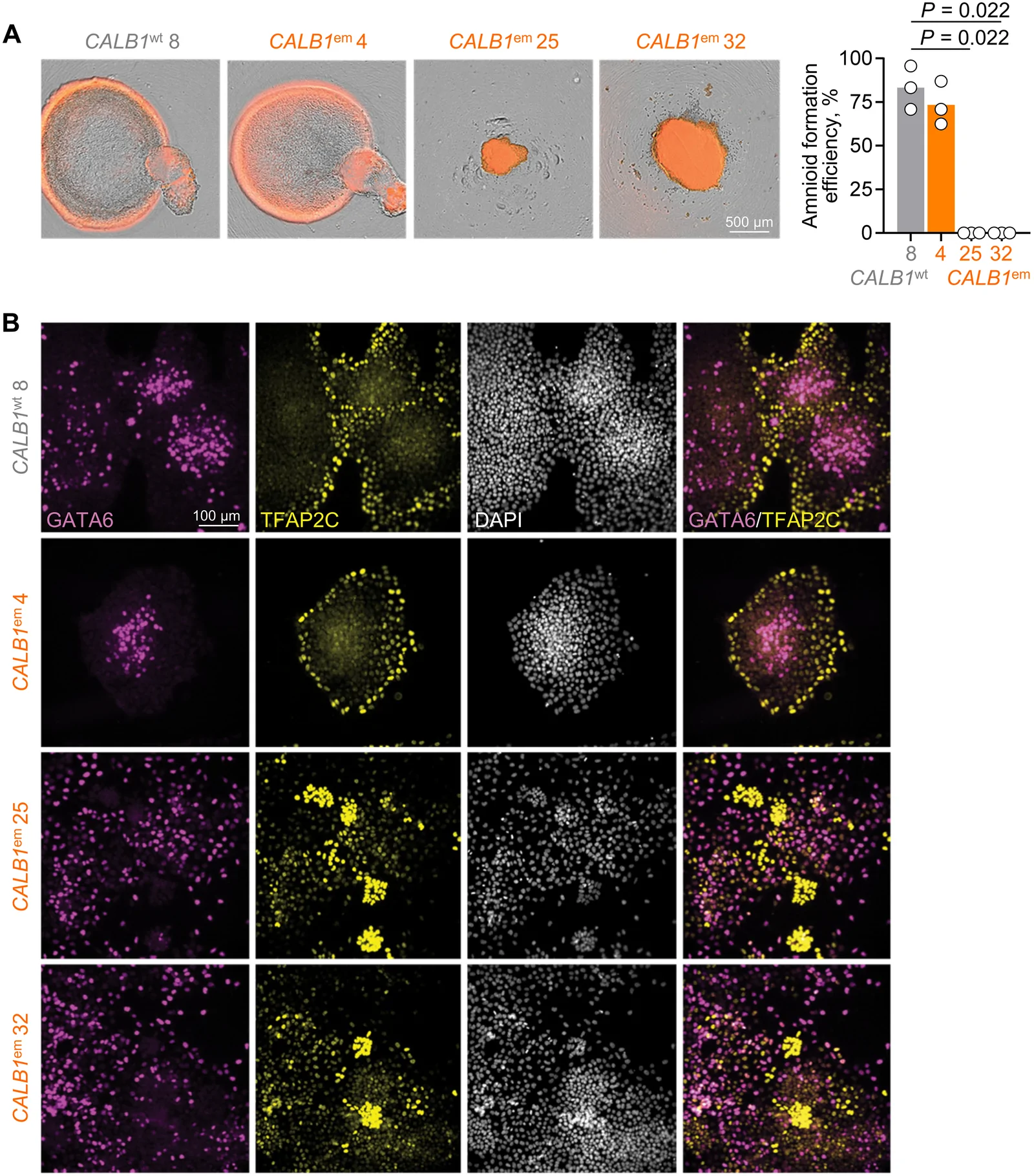
Defective amnioid development of *CALB1*^em^ ESC clones. (**A**) Representative images of NUCLEAR-ID Red–stained PGAs (left) and quantitation of PGAs (right) formed by *CALB1*^wt^ clone 8 and *CALB1*^em^ clones 4, 25, and 32 on day 21. Symbols represent separate plates. *P* values were calculated with one-way repeated-measures ANOVA tests, with Bonferroni correction for multiple comparisons. (**B**) Immunofluorescence staining for the extraembryonic mesoderm marker GATA6 and amniotic ectoderm marker TFAP2C and nuclear DNA staining with DAPI in initial 2D cultures of *CALB1*^wt^ clone 8 and *CALB1*^em^ clones 4, 25, and 32 on day 2 of the PGA formation protocol.

## DISCUSSION

Our increasing understanding of the cis-regulatory potential of HERVs and other transposable elements has highlighted their considerable potential to disrupt and innovate gene function (*34**–**36*). The ectopic expression of *CALB1* under the control of the upstream *HERVH* integration, which is required for optimal cancer cell–intrinsic growth in non–small cell lung cancer (*22*), could be viewed solely as an onco-exaptation event. Our current findings, however, suggest that this effect of the *HERVH 8q21.3* integration underpins and may, therefore, have been selected for optimal development of the human early embryo.

Our studies have indicated a critical role for calbindin in H9 ESCs, and in blastoid and amnioid models thereof, through loss-of-function experiments. However, none of the *CALB1*^em^ H9 ESC clones we established lost *CALB1* function entirely. Instead, they retained residual *CALB1* expression, ranging from 13 to 53%, of a previously unannotated *E4S-CALB1* isoform. While this isoform is also found at low levels in parental H9 ESCs, it becomes the dominant isoform in primed *CALB1*^em^ H9 ESC clones and even more so in naïve *CALB1*^em^ H9 ESC clones, indicating strong selection for residual *CALB1* function. The phenotype of H9 ESCs with complete *CALB1* loss is thus not established here, although it may be too severe to allow the establishment of such clones. In addition to mRNA expression levels, the alternative isoforms may also differ functionally at the protein level. For example, the HERVH-CALB1 isoform lacks the RANBP9 (Ran-binding protein 9)/IMPase (inositol monophosphatase)–interacting domain and exhibits more punctate cytoplasmic localization than the canonical isoform (*22*). Nevertheless, upon overexpression, it reverses the senescence phenotype of calbindin-deficient cells just as effectively as the canonical isoform (*22*). Establishing more subtle differences in functional activity between calbindin isoforms will, therefore, require further, more refined systems.

Calbindin is thought to sustain cell growth and cellular fitness by buffering intracellular Ca^2+^ levels, which would otherwise trigger cellular senescence (*37*). Ca^2+^ levels have been implicated in several pre- and peri-implantation embryo development processes, including mitotic entry and exit in early blastomeres, expression of adhesion molecules mediating blastomere compaction, ICM and TE cell fate decisions, fluid accumulation during blastocoel formation, and trophoblast invasion and blastocyst hatching during implantation (*38**,* *39*). However, alternative functions beyond Ca^2+^ buffering have also been proposed for calbindin, including Ca^2+^ sensing and initiation for signaling cascades, involving several binding partners (*40**–**43*). The precise mechanistic involvement of calbindin in the development processes that fail in its absence will require further context-dependent investigation.

Transposable element onco-exaptation is an important contributor to tumor progression, reflecting their cis-regulatory adaptability, and may additionally reflect tumor exploitation of physiological transposable element functions that also contribute to host fitness (*44**–**46*). Such beneficial and detrimental effects of transposable element integrations create antagonistic pleiotropy, exemplified by *HERVH*-driven *CALB1* expression, which promotes the fitness of preimplantation embryos but also prevents the senescence of lung cancer cells later in life. Owing to the divergence of their LTR sequences (*9*), *HERVH* subfamilies or individual integrations are not always transcriptionally coordinated and can exhibit distinct association with the survival of patients with cancer (*22*, *47**–**50*). Thus, the possible effect of a particular HERV integration on cancer cell or host fitness must be considered as part of the overall effect of all derepressed families and subfamilies.

Expression of the *HERVH* family has been strongly linked with human ESC pluripotency (*13*). In agreement with these findings, our single-cell RNA-seq analysis correlated loss of *CALB1* with differentiation and loss of pluripotency marker expression. However, *CALB1*^em^ clones appear to maintain a pluripotent potential at the population level, likely provided by a minority of individual cells in these otherwise clonal populations that retain the residual expression of *CALB1* and associated pluripotency markers. Prior studies targeting the *HERVH* family in H9 ESCs did not uncover a role for the *HERVH 8q21.3* integration and resulting *CALB1* expression in ESC growth. This is likely due to the divergence of the *HERVH 8q21.3* LTR sequence, reannotated as LTR7u2 (*9*), from the consensus LTR7Y sequences, thereby creating distinct patterns of expression during early embryogenesis. As a result of the LTR sequence divergence, the *HERVH 8q21.3* provirus and, consequently, *CALB1* expression were unaffected by the knockdown strategies that were used to demonstrate the requirement for *HERVH* family expression in human ESC pluripotency (*13*).

Differential transposable element activity, including that of *HERVH* subfamilies, has recently been found by single-cell analyses of human embryos, which identified a new cell type, termed Reject cells, which are characterized by lower *HERVH* expression (*11*). These cells exhibit evidence of DNA-damage response and undergo apoptosis shortly after embryonic gene activation (*11*), thus resembling the phenotype of *CALB1*-deficient cells. Alternatively or additionally, *HERVH*-driven *CALB1* expression may be necessary for early embryo morphogenesis, rather than lineage commitment, a notion supported by the complete failure of *CALB1*^em^ clones to achieve embryonic mesoderm and amniotic ectoderm separation, rather than differentiation, during amnioid formation.

While the invasion of the genomes of our ancestors by the *HERVH* family started over 55 million years ago (*1*), the *HERVH 8q21.3* integration is far more recent, placed between the split from orangutan and gorillas, 8 to 12 million years ago. It is notable that the *HERVH 8q21.3* integration has achieved fixation and become essential for human early embryogenesis in a relatively short evolutionary time. This timing also coincides with additional adaptations of higher primate embryo implantation strategies (*51*). The relatively quick co-option of the *HERVH 8q21.3* provirus in human preimplantation embryo development may not be an isolated example. A parallel study with HERV-K(HML-2) proviruses, a subfamily of HERV-K that includes the most recently acquired, human-specific integrations, has also uncovered an essential role in human early embryogenesis (*52*). The cis-regulatory activity of a HERV-K(HML-2) LTR drives the human-specific expression of *ZNF729*, which, in turn, controls basic cellular functions of the EPI (*52*). Both these examples provide further support for the “addiction” model, proposed to explain the quick co-option of active transposable elements into conserved developmental processes (*53*), whereby the function afforded by a new transposable element integration reduces the reliance and evolutionary constraint on the host-encoded product, thus rendering the new transposable element function essential.

Calbindin may also play important roles in early embryo development in lower mammals and other animals. Developmentally regulated and vitamin D–inducible calbindin expression in chick embryonic yolk sac endodermal cells mediates calcium transport from the yolk sac, the only source of calcium at that stage in chick embryos (*54*). Moreover, *CALB1* is one of two functionally related genes that mediates the reproductive success of mouse oocytes, with its loss inducing apoptosis and DNA damage in aging oocytes (*55*). However, *CALB1* is rapidly down-regulated following the first division of mouse zygotes and is not expressed in subsequent stages of early mouse embryo development. Accordingly, mice deficient in calbindin alone or in combination with other calcium-binding proteins are all viable and exhibit only defects in cerebellar function, resulting from loss of neuronal *CALB1* expression (*56*, *57*).

In contrast to mouse embryogenesis, our findings suggest a critical function for calbindin in human early embryogenesis. This function is further supported by an intolerance of the human *CALB1* gene to loss-of-function variation. Inspection of the Genome Aggregation Database (gnomAD) (*58*) indicates a relative paucity of loss-of-function mutations, with a LOEUF (loss-of-function observed/expected upper bound fraction) score of 0.61. It is, therefore, possible that the *HERVH 8q21.3* integration extended and established an essential function of calbindin in other processes in human preimplantation embryo development, creating the ground for further co-option of this effect in human cancer.

## MATERIALS AND METHODS

### Ethics statement

This study was approved by the UK Human Fertilization and Embryology Authority (HFEA): research license numbers R0162, R0397, R0401 and R0152 and independently reviewed by the Health Research Authority’s Research Ethics Committee IRAS projects 308099, 252286, and 272218. The process of license approval entailed independent peer review along with consideration by the HFEA License and Executive Committees and the Research Ethics Committee. All research on human embryos is compliant with the HFEA Code of Practice and has undergone multiple inspections by the HFEA since the license was granted. Informed consent was obtained from all couples that donated surplus embryos following infertility treatment. Before giving consent, donors were provided with all the necessary information about the research project, an opportunity to receive counseling, and the conditions that apply within the license and the HFEA Code of Practice. Donors were informed that embryos used in the experiments would be stopped before 14 days postfertilization and that subsequent biochemical and genetic studies would be performed. Informed consent was also obtained from donors for all the results of these studies to be published in scientific journals. No financial inducements were offered for donation. Embryos surplus to the patient’s in vitro fertilization treatment were donated cryopreserved and were transferred to the Francis Crick Institute and University of Cambridge where they were thawed and used in the research project.

Human ESC experiments were performed at the Stem Cells and Organoids Platform within the Human Biology STP at the Francis Crick Institute. All experiments followed the Guidelines for Stem Cell Research and Clinical Translation published by the International Society for Stem Cell Research and the UK Code of Practice for the Use of Human Stem Cell Lines. The models used are nonintegrated and composed of extraembryonic tissue only complying with section 2.2.1A of the International Society for Stem Cell Research Guidelines. Although these models are composed of multicellular structures, they are not complete and are not equivalent to the in vivo human embryo. The H9 hES cell line is used under agreement from the Steering Committee for the UK Stem Cell Bank and Use of Human Stem Cells (SCSC11-06). All work in this study is compliant with all relevant UK guidelines and regulations.

### RNA-seq, read alignment, normalization, and quantitation

RNA from primed and naïve ESCs in bulk and from single cells was sequenced as previously described (*22*). Paired-end fastq files were unzipped using pigz and trimmed with Trimmomatic using a minimum length of 35 nucleotides, a sliding window of 3:20, and the illuminaclip variable set to 2:40:10:2. For quality checking, FastQC was run. HISAT2 was used to align reads to the human genome (GRCH38) with the settings -p 8 -q -k 5 –no-unal and output as a SAM file. SAMtools was used to convert SAM to BAM files and sort BAM files by read name (-n). The featureCounts function of the Subread package was used to count primary read alignment to genes as annotated by GENCODE version 29, with settings -p -C -B -T 8 --primary. Reads aligned to repetitive elements were counted using the settings -p -C -B -f -T 8 --primary using the custom Dfam 2.0 library, as previously described (*59*). Alignments were visualized on the Integrative Genomics Viewer 2.19.6 (*60*). Raw count tables for genes and repeat elements were read into R with the read function of the data table package. After concatenation, counts were normalized using the DESeq2 package. Read count tables from bulk RNA-seq were additionally imported into Qlucore Omics Explorer version 3.9 (Qlucore, Lund, Sweden) for further downstream expression analyses and visualization. Downstream analyses and visualization of single-cell RNA-seq data were carried out with 10× Genomics Loupe Browser version 9.0.

### Functional gene annotation by gene ontology

Pathway analyses were performed using g:Profiler (*61*), as previously described (*48*).

### Comparative genomic analyses

Multiple genomic sequences from the indicated species were aligned using the Ensembl Comparative Genomics tool (www.ensembl.org/info/genome/compara/index.html) and cross-checked with the UCSC Genome Browser (https://genome.ucsc.edu), 30 Primates track, as previously described (*62*).

### Reverse transcriptase–based quantitative PCR (RT-qPCR)

RNA was extracted from cells and blastoids using the RNeasy Mini Kit and from human embryos using the RNeasy Micro Kit followed by cDNA transcription using the High-Capacity cDNA Reverse Transcription Kit. For embryos 6 and 15, the RecoverAll Total Nucleic Acid Isolation Kit for formalin-fixed, paraffin-embedded tissues was used to extract the RNA. Quantitative PCR was run using Fast SYBR Green Master on the QuantStudio3 system (Applied Biosystems). Primers (table S1) were used at a final concentration of 0.2 μM and an annealing temperature of 60°C with 20 ng of cDNA placed into a 10-μl reaction for cells and blastoids and 1 μl of total elute from human embryos.

### Human ESCs

The human ESC line WA09 H9 was routinely cultured in mTESR1 Basal Medium completed with mTESR1 Supplement at 37°C in 5% CO_2_. H9 cells were fed daily and grown in Matrigel hESC-Qualified Matrix–coated six-well tissue culture plates and clump passaged using Versene Solution twice per week at a 1/20 dilution.

### Chemical resetting

H9 cells were chemically reset to naïve cells, as previously described (*28*). DR4 mouse embryonic fibroblasts (MEFs), irradiated, were seeded onto six-well plates coated with 0.1% gelatine 1 to 2 days before the conversion at a density of ∼2 × 10^5^ cells per well. The H9 cells were then passaged using TyplE Select Enzyme and seeded at 5 × 10^5^ cells per well in mTESR1 with 10 μM ROCK inhibitor (ROCKi, Y-27632) for 24 hours and a further 24 hours in mTEST1 without ROCKi until medium-sized colonies had formed. Chemical resetting medium was then applied at hypoxia (5% O_2_) for 3 days with the chemical resetting medium being replenished every 24 hours. The cells were then maintained in PXGL at hypoxia, fed every day, and passaged twice a week using StemPro Accutase Cell Dissociation Reagent and 10 μM ROCKi and Matrigel hESC-Qualified Matrix for the first 24 hours. The naïve cell colonies were then assessed for morphology, number, and CD75 and SUSD2 expression via flow cytometry. In addition, the naïve cells were assessed for karyotype at passages 10 and 20.

### Blastoid formation assay

Blastoids were generated using previously described methods (*29*, *30*). Briefly, Aggrewell400 24-well plates were prepared with 500 μl of Anti-Adherence Rinsing Solution, spun at 1500*g* for 5 min, and washed with 2 ml of N2B27 medium. Naïve cells were removed from feeder cells using TyplE Select Enzyme for 2 min and 30 s at 37°C and the tapping technique. Aggregates were broken up by pipetting in wash medium [DMEM (Dulbecco’s modified Eagle’s medium)/F-12 + 0.1% bovine serum albumin (BSA)] before counting. A total of 1 × 10^5^ cells per well were then seeded in 800 μl of aggregation medium (N2B27 + 10 μM ROCKi +0.3% BSA) for 24 hours and fed with 50% 2× PALLY medium at 48 hours and with 50% 1× PALLY medium at 72 hours. Following this, the blastoids were fed with LY medium (N2B27 + 4 μM lysophosphatidic acid + 10 μM ROCKi) at 50% every 24 hours until termination at day 4/5.

### PGA formation assay

PGAs were cultivated as previously described for H1 cells (*33*), with the following modifications: Bone morphogenetic protein 4 (BMP4) levels were increased to 10 ng/ml, and seeding density was increased to 500 cells per well in a 96-well plate at day 0. PGAs were grown for up to 21 days, stained with NUCLEAR-ID Red DNA stain, and imaged using the IncuCyte Zoom (Essen BioScience Ltd.) for the morphological assessment of amniotic sac–like structure formation. For immunofluorescence staining, H9 clones were fixed in 2D after 48 hours of step-wise differentiation with BMP4 and Chiron in Greiner 96-well μClear plates (Greiner). Cells were imaged using the scanR-automated inverted epifluorescence microscope system IX83 (Evident Scientific, formerly known as Olympus). Images were acquired with a 20× plan (UCPLFLN) fluorescence objective (numerical aperture, 0.7) and a scientific complementary metal-oxide semiconductor (Orca Flash 4.0, Hamamatsu) camera. LED (light-emitting diode)–based illumination (SpectraX LED, Lumenco) was used for excitation. Evident Scientific scanR Acquisition Software (Version 3.4.1) was used to operate the microscope. FIJI software was used for processing images.

### Human day 5/6 embryo cultivation

Vitrified human embryos were thawed using Vit Kit-Thaw. Human embryos were cultured at 37°C and in 5% CO_2_ in EmbryoSlide+ culture dishes in an EmbryoScope+ time-lapse incubator (VitroLife) containing equilibrated Global medium supplemented with protein supplement (5 mg/ml) and covered with mineral oil.

### Immunofluorescence

Cell monolayers and blastoids were seeded on μ-Slide 8-well high ibidi dishes and formalin fixed using 4% paraformaldehyde for 15 min at room temperature. Samples were then permeabilized in 0.2% Triton X-100 and 1% BSA in phosphate-buffered saline (PBS) for 1 hour at room temperature before staining with primary and secondary antibodies (table S1). Primary antibody (at 1:200 dilution) incubation was carried out at 4°C overnight followed by three 5-min washes in PBS. Secondary antibody (at 1:1000 dilution) incubation was carried out at room temperature for 2.5 hours followed by three 15-min washes in PBS. Samples were counterstained with NucBlue Fixed Cell ReadyProbes Reagent [4′,6-diamidino-2-phenylindole (DAPI)] for nuclear staining and, when possible, with Phalloidin Labelling Probe Alexa Fluor 568. Human embryos were transferred into four-well tissue culture plates using The Stripper Pipettor under a dissecting microscope, washed three times in 4% paraformaldehyde, and then fixed for 20 min at room temperature. This was followed by three washes in PBS with 0.1% Tween 20. Permeabilization was carried out using 0.5% Triton X-100 in PBS for 20 min on a rotating shaker at room temperature, blocked in 10% donkey serum in PBS with 0.1% Tween 20, and incubated with a primary antibody (1:100 to 1:300) in blocking solution overnight at 4°C. This was followed by three washes in PBS with 0.1% Tween 20 and secondary antibody (1:300) incubation for 1 hour on a rotating shaker at room temperature in blocking solution. After three final washes, the samples were then moved into VECTASHIELD Antifade Mounting Medium with DAPI (1:30) in PBS with 0.1% Tween 20. Cells, blastoids, and embryos were imaged using the Zeiss AxioObersver.Z1, Nikon CSU-W1, and Zeiss LSM 880 with Airyscan, respectively (Carl Zeiss AG and Nikon Corporation). All samples were placed in PBS in ibidi dishes before imaging.

### Flow cytometry

Cell suspensions were stained for 30 min with the indicated primary antibodies (table S1) in 1% (w/v) BSA in PBS (at 1:200 dilution) and were washed with the same buffer before incubation for 30 min with the isotype-matched secondary antibodies (at 1:1000 dilution). Live/dead cell discrimination was achieved with the LIVE/DEAD Fixable Near-IR Dead Cell Stain Kit. Samples were run on a BD Biosciences LSR Fortessa using FACSDiva version 8.0 and analyzed with FlowJo version 10. Gating strategies for individual cell experiments are shown in fig. S16.

### Cas9-mediated editing through nucleofection and lipofection

DR4 MEFs, irradiated and puromycin-resistant, were seeded in six-well tissue culture plates at 1 × 10^6^/well in high-glucose DMEM supplemented with 20% fetal bovine serum. The day before transfection, H9 cells were treated with 10 μM ROCKi for 16 to 20 hours. On the day of transfection, the target cells were passaged using StemPRO Accutase Cell Dissociation Reagent, and 2 × 10^6^ cells were transfected with 4 μg of total DNA of pSpCas9(BB)-2A-Puro (PX459) V2.0 with guide RNAs (table S1) as previously described for calbindin deletion using the P3 Primary Cell 4D-Nucleofector X Kit L. Cells were then seeded in 10 μM ROCKi and exposed to cold shock at 32°C for 48 hours with puromycin (0.5 μg/ml) selection starting after 24 hours for 48 hours. The cells were then left to recover until intact colonies started to form. After the establishment of indel frequency using the Cas-Analyzer by CRISPR RGEN Tools, the approximate number of colonies to be picked was calculated. In this case, two rounds of 20 colonies were manually collected with a 20-μl pipette tip set to 5 μl, transferred into a Matrigel hESC-Qualified Matrix–coated 96-well tissue culture plate, and supplemented with 10 μM ROCKi for 24 hours. The clonal colonies were then validated using amplicon sequencing, Western blotting, and karyotyping. For Cas9-mediated *HERVH 8q21.3* targeting, 2 × 10^6^ naïve H9 cells were reverse transfected with 1.5 μg of total DNA of pSpCas9(BB)-2A-Puro (PX459) V2.0 with guide RNAs (table S1) as previously described for *HERVH 8q21.3* deletion or a control plasmid (pmaxGFP) using the Lipofectamine 3000 Transfection Reagent. Colonies that grew following puromycin (0.5 μg/ml) selection for 48 hours were further expanded for 8 days before RNA isolation and subsequent RT-qPCR for *CALB1* and *HERVH-CALB1* transcription. To prevent the amplification of DR4 MEF housekeeping gene cDNA, a human *HPRT1*-specific pair of primers was used for normalization (table S1).

### siRNA-mediated *CALB1* knockdown

For siRNA knockdown of *CALB1*, H9 cells were single-cell dissociated with gentle cell dissociation reagent for 10 min at 37°C and transfected with 25 nM *CALB1*-targeting and nontargeting control siRNAs (table S1) using the Lipofectamine RNAiMAX Transfection Reagent. The cells were then seeded at a density of 125,000 cells/ml in mTeSR1 supplemented with 10 μM ROCKi. The following day, cells were washed once with Dulbecco’s PBS and fed with mTeSR1.

### EdU incorporation and click-chemistry detection

For EdU incorporation, live cells were washed once with Dulbecco’s PBS and then incubated with EdU (2.5 μg/ml) diluted in mTeSR1 for 2 hours before fixation. EdU detection was performed before secondary antibody incubation using the Click-iT EdU Cell Proliferation Kit for Imaging, according to the manufacturer’s instructions.

### Image analysis following siRNA-mediated *CALB1* knockdown

Single-cell segmentation and feature extraction were performed as previously described (*63*). Segmented objects were filtered by area (>50 px) to eliminate debris, and cells positive for EdU, phospho-Histone H3 (Ser^10^), or calbindin were defined by manual gating of marker expression.

### Genomic DNA extraction, karyotyping, and amplicon sequencing

Genomic DNA was extracted using the DNeasy Blood & Tissue Kit, and karyotyping was conducted at the in-house facility using the illumina Nextera Flex protocol, followed by alignment to the human genome and copy number being performed using the QNASeq package (*64*). Amplicons to be sequenced were amplified using KOD Hot Start Master Mix or PrimeSTAR GXL Premix. Products were then cleaned up using Beckman Coulter Agencourt AMPure XP beads on a magnetic stand. The final product was sequenced at the in-house facility using the illumina MiSeq platform.

### Western blotting

Protein was extracted from cells using radioimmunoprecipitation assay buffer and quantified using the Pierce BCA Protein Assay Kit. Ten micrograms of total protein in Sample Buffer, Laemmli 2× Concentrate was loaded per lane and run on 4 to 20% Mini-PROTEAN TGX Precast Protein Gels, 10-well, at 120 V for 1 hour. Transfer was conducted using Trans-Blot Turbo Mini 0.2-μm Nitrocellulose Transfer Packs using the Trans-Blot Turbo Transfer System (BIO-RAD). Membranes were stained with the indicated primary antibodies (at 1:200 dilution) and horseradish peroxidase–conjugated secondary antibodies (at 1:1000 dilution) (table S1). Protein bands were visualized using Clarity Western ECL Substrate on the Amersham ImageQuant 800 (Cytiva Life Sciences). The anti-calbindin antibody used here for Western blotting in Fig. 3A is a rabbit polyclonal antibody (Atlas Antibodies, cat. no. HPA023099), raised by immunization with amino acid residues 7 to 96 of the full-length canonical calbindin. The HERVH-CALB1 and E4S-CALB1 isoforms are missing amino acid residues 1 to 57 and 78 to 105, respectively, thereby reducing the overlap with the immunogen used for the generation of this antibody. Although the dominant epitopes within the immunogen have not been mapped, the lack of 51 and 19 amino acid residues from the HERVH-CALB1 and E4S-CALB1 isoforms, respectively, of a total of 90 amino acid residues in the immunogen very likely reduces the sensitivity of this reagent for the alternative isoforms compared with the canonical isoform (*22*). Uncropped blots are provided in the Supplementary Materials.

### Cell viability assays

Cell proliferation and growth were measured using CellTiter-Glo 2.0 Cell Viability Assay. Cells were seeded at a density of 2 × 10^3^ in a Matrigel hESC-Qualified Matrix–coated 96-well plate, and viability was assessed on days 0, 1, 2, and 3. Luminescence was measured using the Tecan Spark Microplate Reader. To assess senescent cells, the CellEvent Senescence Green Detection Kit was used according to the manufacturer’s instructions, and cells were subsequently imaged.

### Protein structure prediction and visualization

Structures of the canonical and alternative calbindin isoforms were modeled on the basis of the structure of the canonical calbindin (Protein Data Bank ID: 6FIE) predicted by AlphaFold (https://alphafold.ebi.ac.uk). Structures were visualized in the Research Collaboratory for Structural Bioinformatics Protein Data Bank Mol* 3D Viewer (www.rcsb.org/3d-view).

### Trilineage assessment

Propensity to differentiate into endoderm, ectoderm, and mesoderm lineages was assessed by exposing H9 clones to the STEMdiff Trilineage Differentiation Kit. Cell densities were optimized to be 2 × 10^5^, 5 × 10^4^, and 2 × 10^5^ cells for the ectoderm, mesoderm, and endoderm, respectively, in a Matrigel hESC-Qualified Matrix–coated six-well plate. Positive staining for lineage markers (ectoderm: PAX6 and nestin; mesoderm: brachyury T and CXCR4; endoderm: SOX17 and CXCR4) (table S1) was conducted using the LSRFortessa Cell Analyzer (BD Biosciences).

### Statistical analyses

Statistical comparisons were made using GraphPad Prism 10.3 (GraphPad Software), SigmaPlot 14.0, Qlucore Omics Explorer version 3.9, or R (versions 3.6.1 to 4.0.0). Parametric comparisons of normally distributed values that satisfied the variance criteria were made by unpaired or paired Student’s *t* tests or one-way analysis of variance (ANOVA) tests with Bonferroni or Holm-Sidak correction for multiple comparisons or with one-way repeated-measures ANOVA tests, with Bonferroni or Dunnett’s correction for multiple comparisons (paired data). Data that did not pass the variance test were compared with nonparametric two-tailed Mann-Whitney rank-sum tests (for unpaired comparisons) or a Kruskal-Wallis one-way ANOVA on ranks with Dunn’s multiple-comparisons correction.

## Supplementary Materials

### The PDF file includes:

Figs. S1 to S16 Table S1 Legends for movies S1 to S10 Legends for data files S1 and S2 Uncropped Western blots References
