# Supplementary material for: Endogenous retrovirus control of calbindin is essential for human early embryogenesis
Source: Sci Adv. 2026 Oct 7;12(41):eaee4523. doi: 10.1126/sciadv.aee4523 (PMC13644760; doi:10.1126/sciadv.aee4523)
Supplement: Supplementary file 1 [file sciadv.aee4523_sm.pdf]

Science Advances
AAAS

Supplementary Materials for

Endogenous retrovirus control of calbindin is essential for human early embryogenesis

Judith Pape *et al.*

Corresponding author: George Kassiotis, george.kassiotis@crick.ac.uk

Sci. Adv. **12**, eaee4523 (2026)
DOI: 10.1126/sciadv.aee4523

The PDF file includes:

Figs. S1 to S16
Table S1
Legends for movies S1 to S10
Legends for data files S1 and S2
Uncropped Western blots
References

Other Supplementary Material for this manuscript includes the following:

Movies S1 to S10
Data files S1 and S2

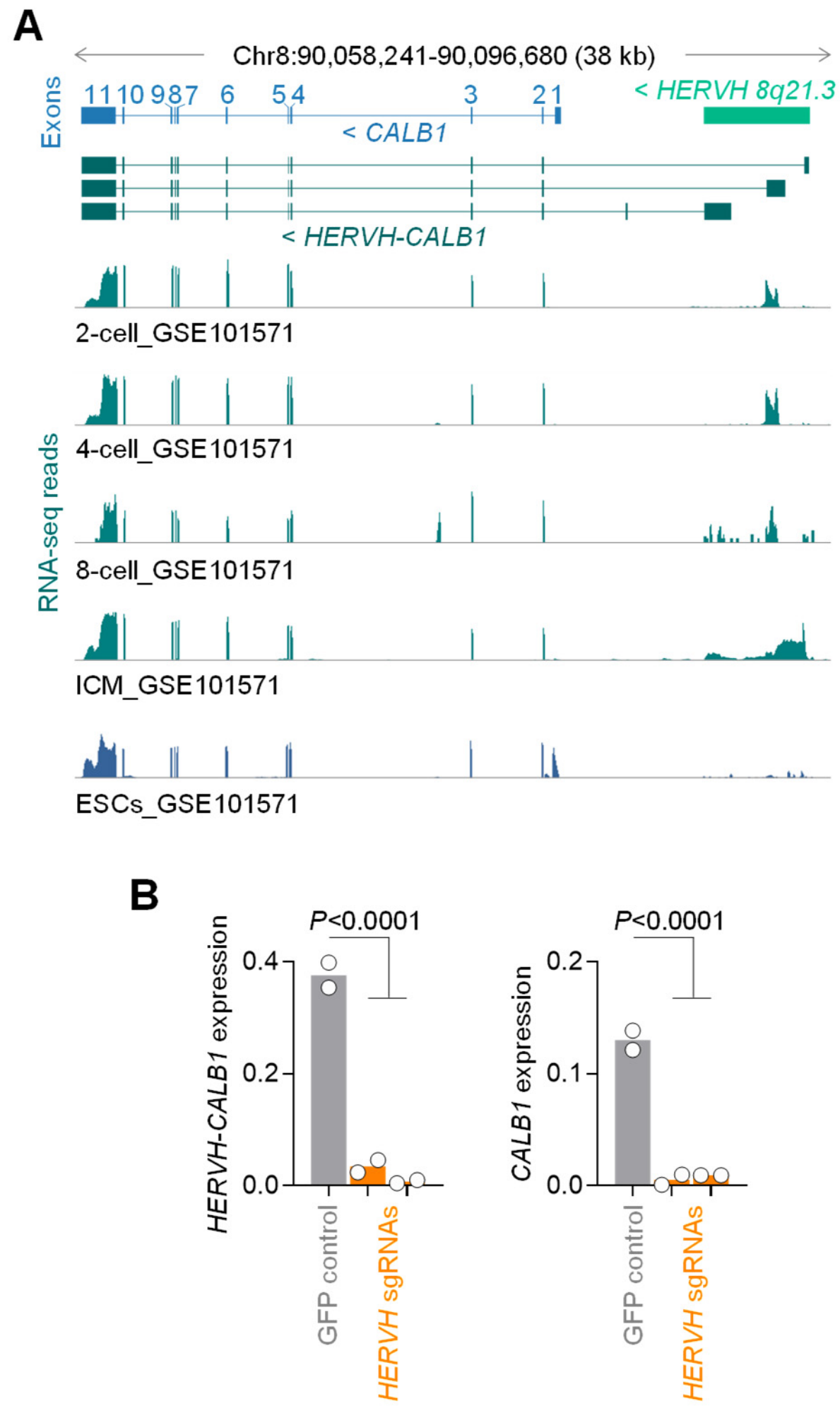

Fig. S1. *HERVH* integration and control of early embryo *CALB1* expression

(**A**) The *CALB1* locus and integrated *HERVH* provirus, with the GENCODE-annotated canonical *CALB1* and alternative *HERVH*-driven transcripts; and RNA-seq traces, determined by analysis single-cell RNA-seq data (GSE101571) in 2–8-cell stage and ICM cells from human preimplantation embryos, and in cultured ESCs. Data were downloaded from the dbEmbryo database (*65*).

(**B**) *HERVH*-driven and canonical *CALB1* isoform expression, determined by isoform-specific RT-qPCR, in naïve H9 cells following transfection with CRISPR/Cas9 endonuclease and sgRNAs targeting the *HERVH 8q21.3* integration (*HERVH* sgRNAs) or with a non-targeting control plasmid (GFP control). Bars show biological replicates at the transfection level and symbols represent technical replicates at the qPCR level. P values were calculated with Student's t-tests comparing the combined *HERVH 8q21.3*-targeting replicates with the control.

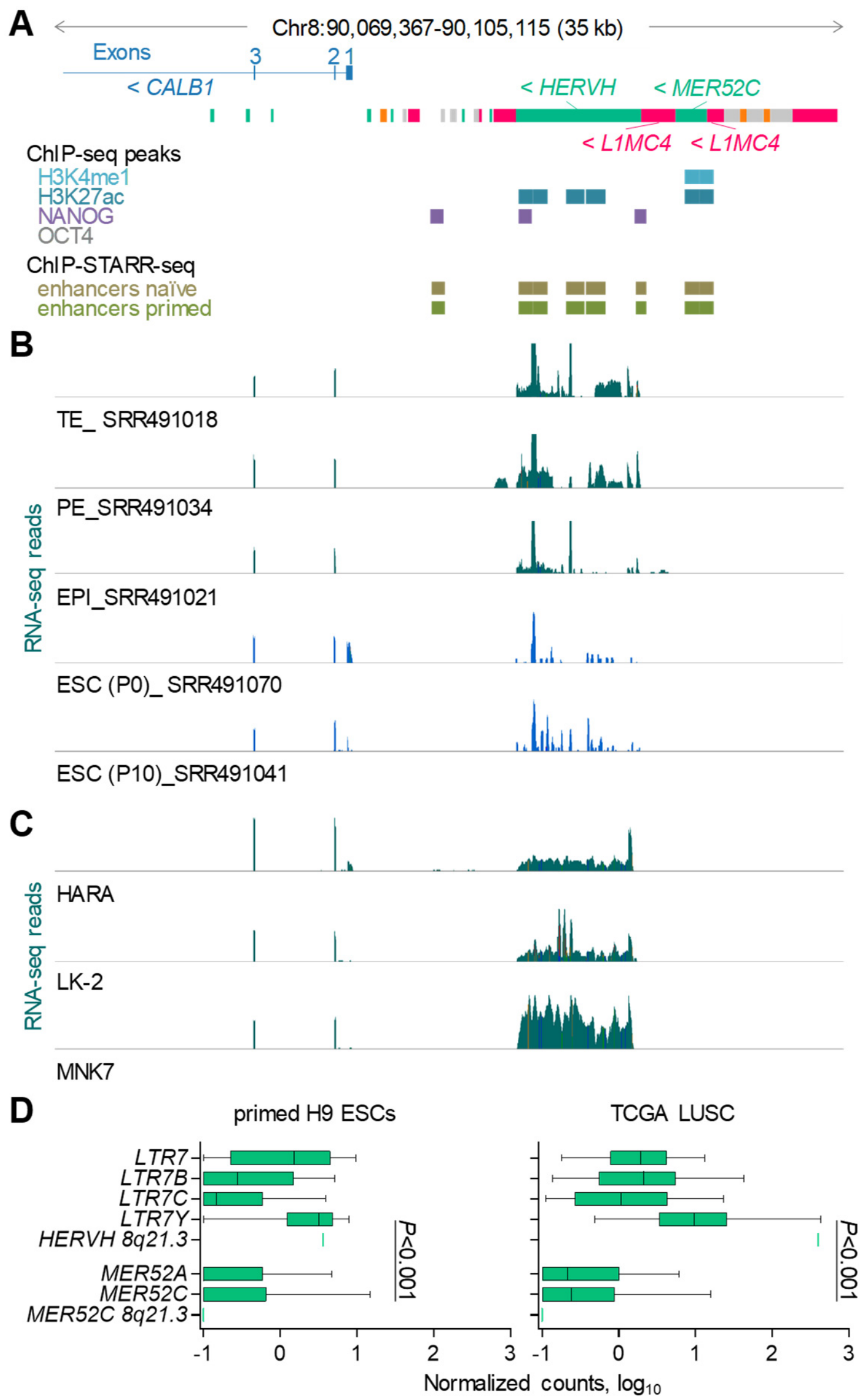

Fig. S2. Enhancer and promoter landscape at the *CALB1* locus

(**A**) Chromatin immunoprecipitation followed by sequencing (ChIP-seq) peaks for histone modifications, NANOG and OCT4 transcription factor binding, and functional enhancers in the promoter region of *CALB1* and upstream *HERVH* and *MER52C* LTR element integration identified by analysis of data from primed and naïve human ESCs (*31*).

(**B**) RNA-seq traces, determined by analysis single-cell RNA-seq data (GSE36552), in TE, PE and EPI cells from human preimplantation embryos, and passages 0 and 10 of ESC outgrowths (P0 and P10, respectively).

(**C**) RNA-seq traces, determined by analysis bulk RNA-seq data in HARA and LK-2 lung squamous carcinoma and MNK7 gastric tubular adenocarcinoma cells from CCLE.

(**D**) Normalized counts of expression of the *HERVH* and *MER52C* element upstream of the *CALB1* gene, and of the *HERVH* and *MER52A/C* subfamilies in primed H9 cells (n=3) or lung squamous

carcinoma (LUSC) samples from TCGA (n=370). Box plots show the upper and lower quartiles, center lines show the median and whiskers represent the 5-95 quartile range. P values were calculated with non-parametric two-tailed Mann–Whitney rank sum tests.

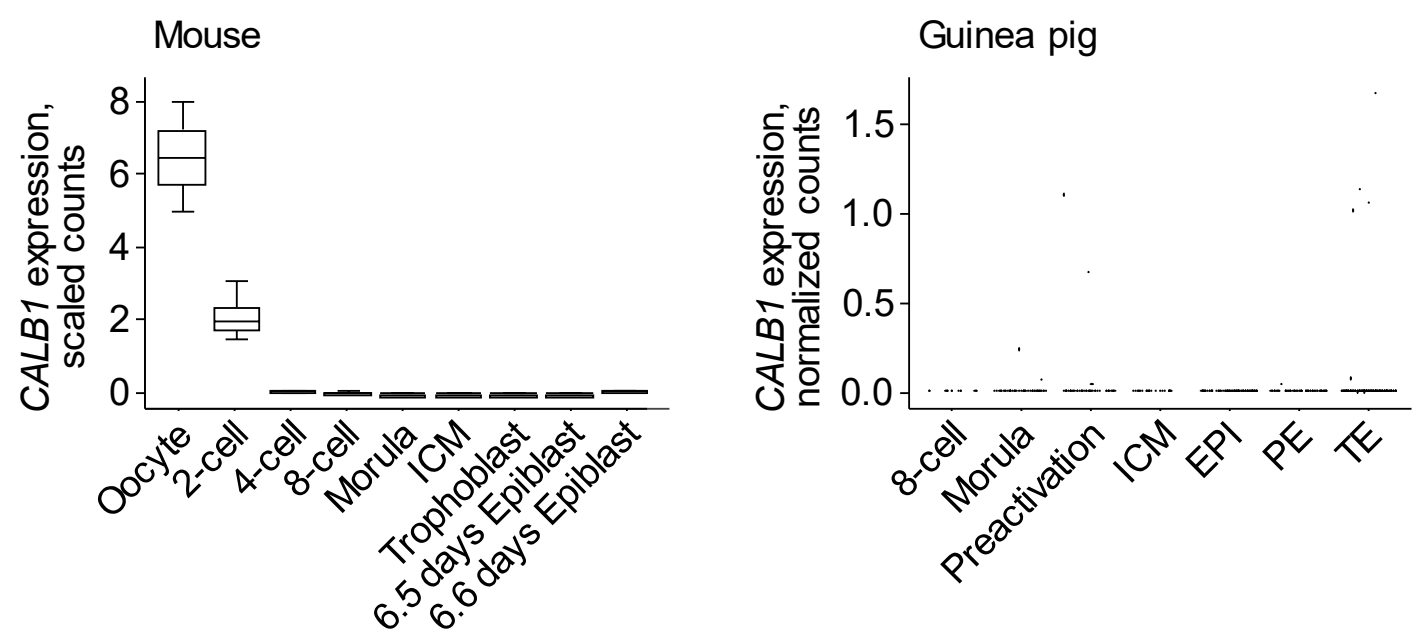

Fig. S3. *CALB1* expression in mouse and Guinea preimplantation embryos

CALB1 expression in the indicated cell types determined by analysis of RNA-seq data from mouse (EmAtlas (*66*)) and Guinea pig preimplantation embryos (*67*).

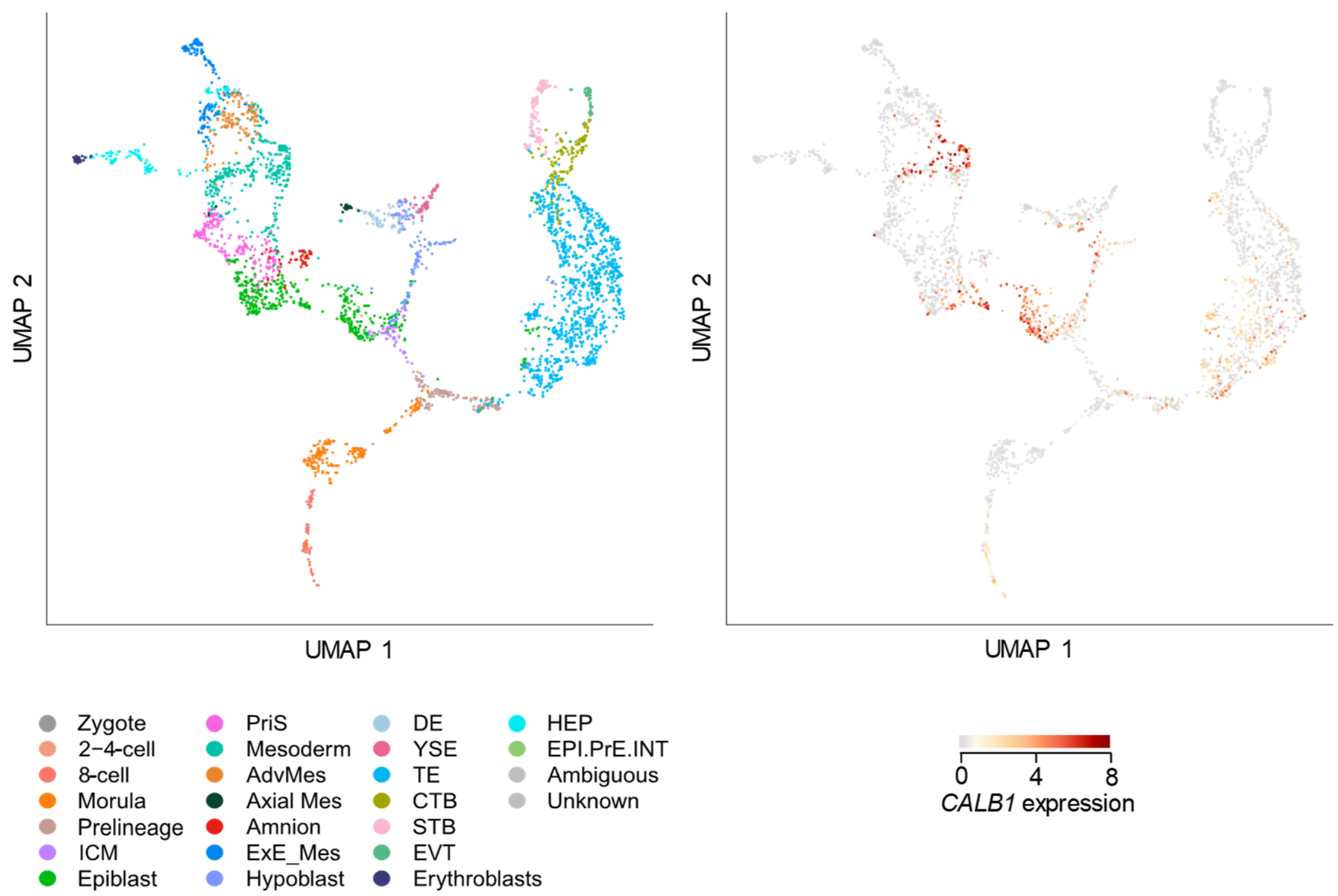

Fig. S4. *CALB1* expression in human preimplantation embryos

UMAP clustering of human preimplantation embryo cell lineages from a reference single-cell RNA-Seq collection (*32*) (*left*) and projection of *CALB1* expression onto the same UMAP (*right*). ICM, inner cell mass; PriS, primitive streak; AdvMed, advanced mesoderm; Axial Mes, axial mesoderm; Exe_Mes, extraembryonic mesoderm; DE, definitive endoderm; YSE, yolk sac endoderm; TE, trophectoderm; CTB, cytotrophoblast; STB, syncytiotrophoblast; EVT, extravillous trophoblast; HEP, haemato-endothelial progenitors; EPI.PrE.INT epiblast prelineage.

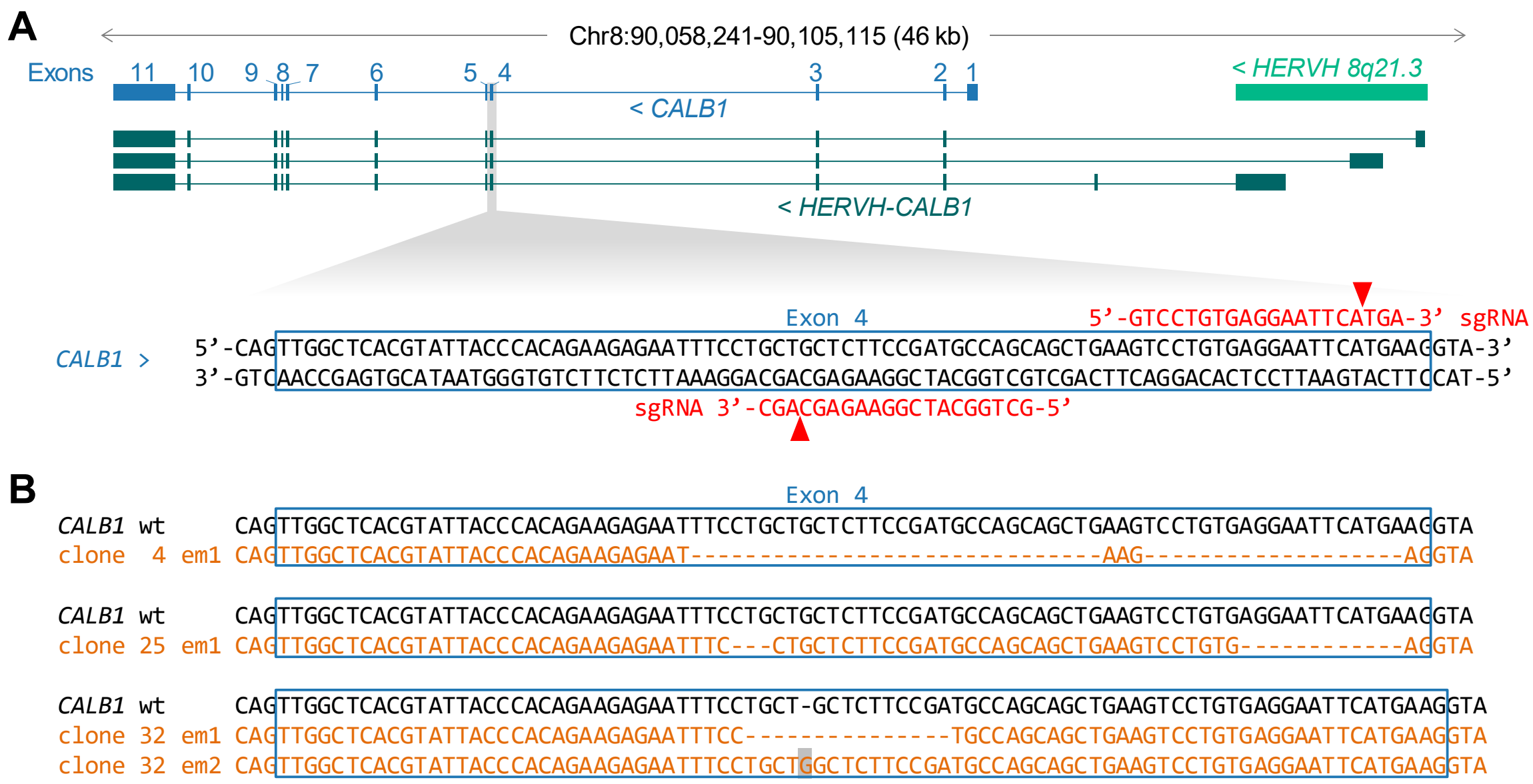

Fig. S5. Genotypes of CRISPR/Cas9-targeted *CALB1*^{em} ESC clones

(**A**) Position of the targeted exon 4 in relation to the *CALB1* locus on the negative strand (*top*) and sequence of exon 4 in the sense orientation, with the sequence and position of the sgRNAs shown in red (*bottom*). Arrows indicate the position of Cas9-mediated cuts.

(**B**) DNA sequence of *CALB1* exon 4 alleles amplified from genomic DNA from the indicated *CALB1*^{em} clones, determined by DNA deep-sequencing and aligned to the *CALB1* wild-type (wt) allele. Two distinct alleles were detected in *CALB1*^{em} clone 32 (em1 and em2, respectively), whereas single alleles were detected in *CALB1*^{em} clones 4 and 25.

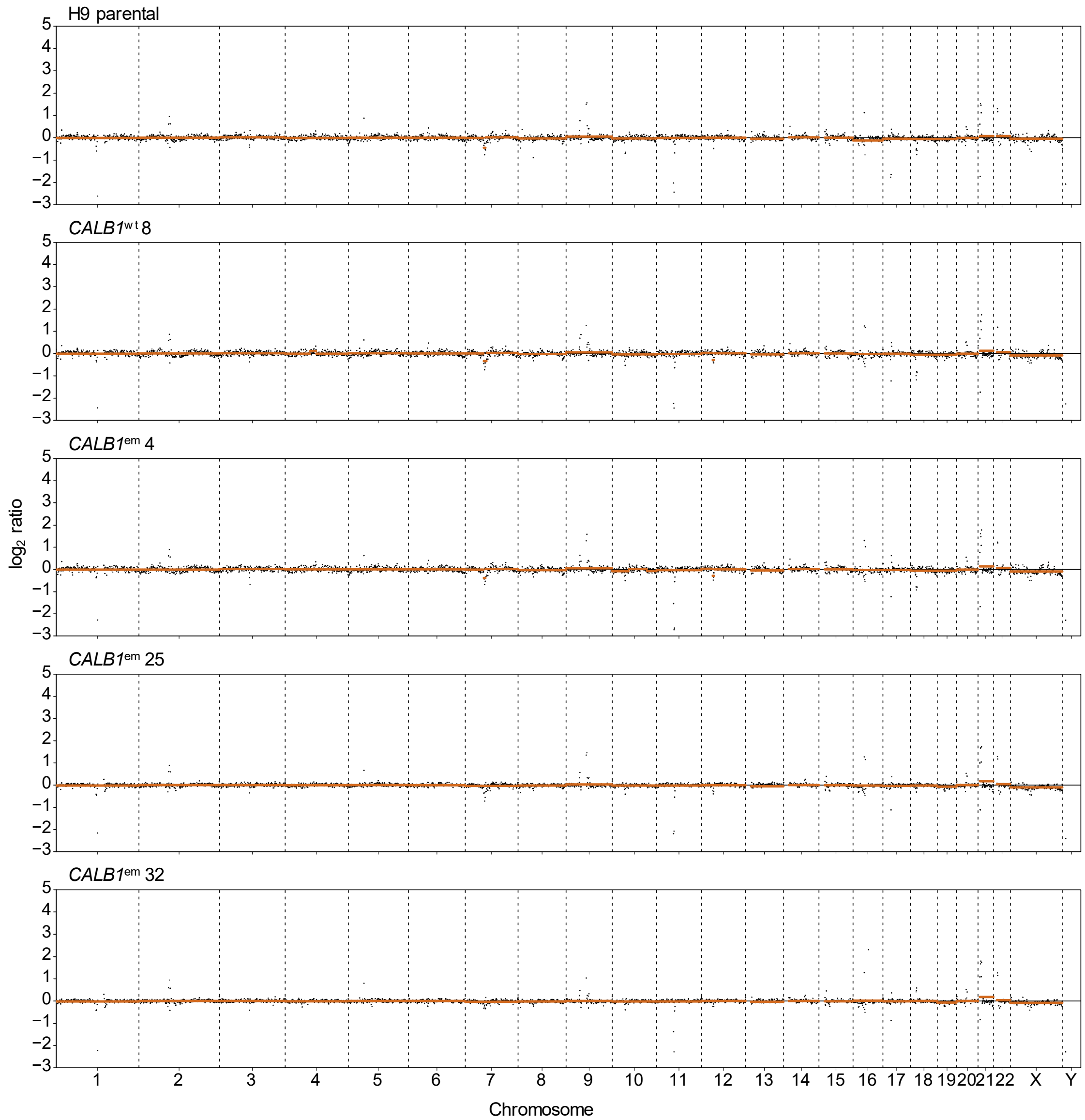

Fig. S6. Karyotypes of CRISPR/Cas9-targeted *CALB1*^{em} ESC clones

Karyotypes of the indicated *CALB1*^{em} clones, determined by genomic DNA sequencing. The karyotypes of parental H9 cells and *CALB1*^{wt} clone 8 are also included for comparison.

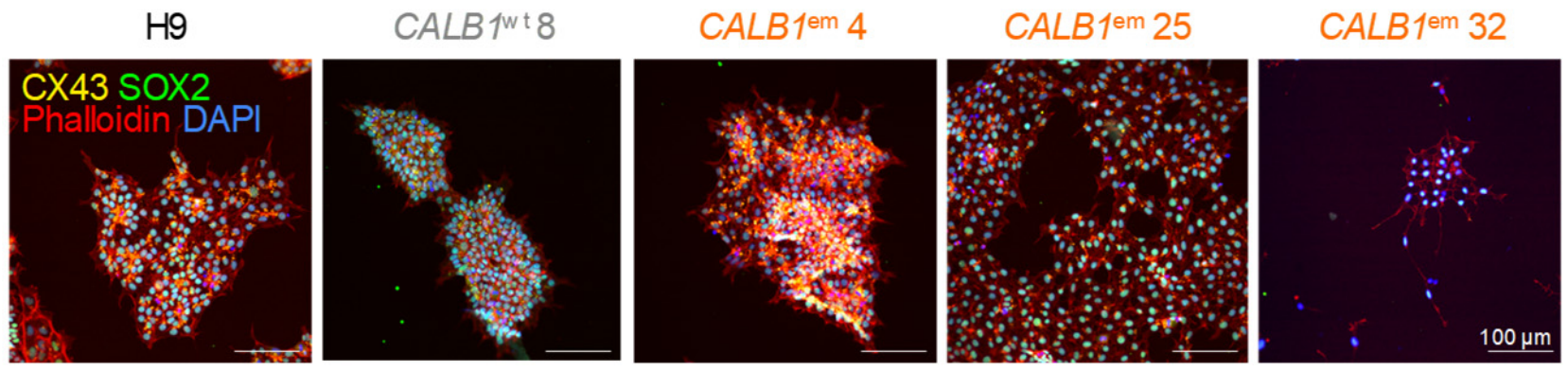

Fig. S7. Loss of connexin 43 in *CALB1*^{em} ESC clones

Immunofluorescence staining for connexin 43 (CX43), lineage marker SOX2, filamentous actin with Phalloidin and nuclear DNA staining with DAPI in *CALB1*^{em} ESC clones. Parental H9 cells and *CALB1*^{wt} clone 8 are included for comparison.

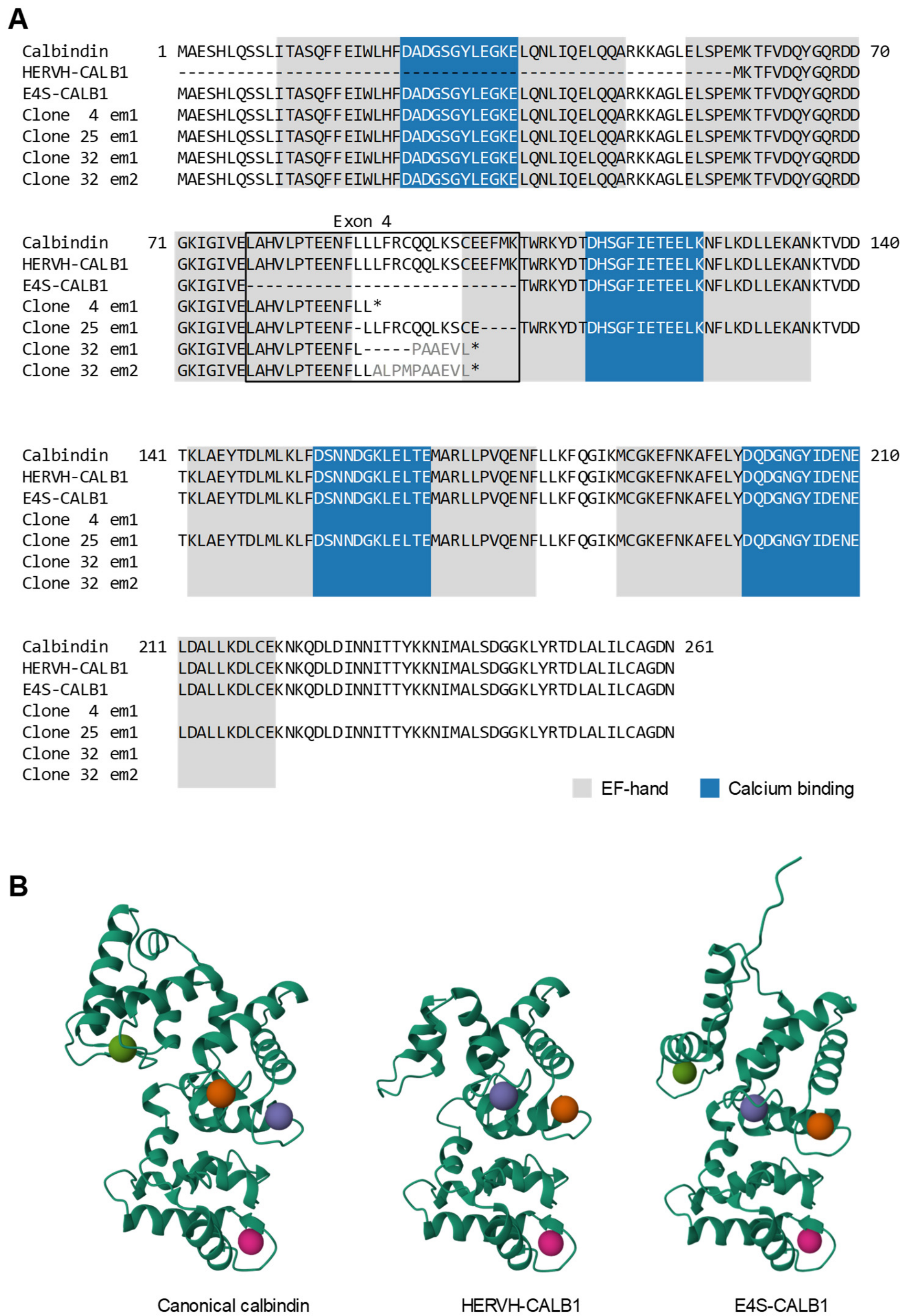

Fig. S8. Calbindin isoforms expressed in *CALB1*[em] ESC clones

(**A**) Alignment of the calbindin amino acid sequence, deduced from the translation of deep-sequenced full-length cDNA amplified from *CALB1*[em] clones. Exon 4, EF-hands and calcium-bonding domains are indicated. The canonical calbindin sequence is also included as the reference.

(**B**) AlphaFold models of the canonical calbindin, *HERVH*-driven calbindin (HERVH-CALB1) and exon 4-skipping (E4S-CALB1) calbindin isoforms. Calcium ions are depicted as spheres.

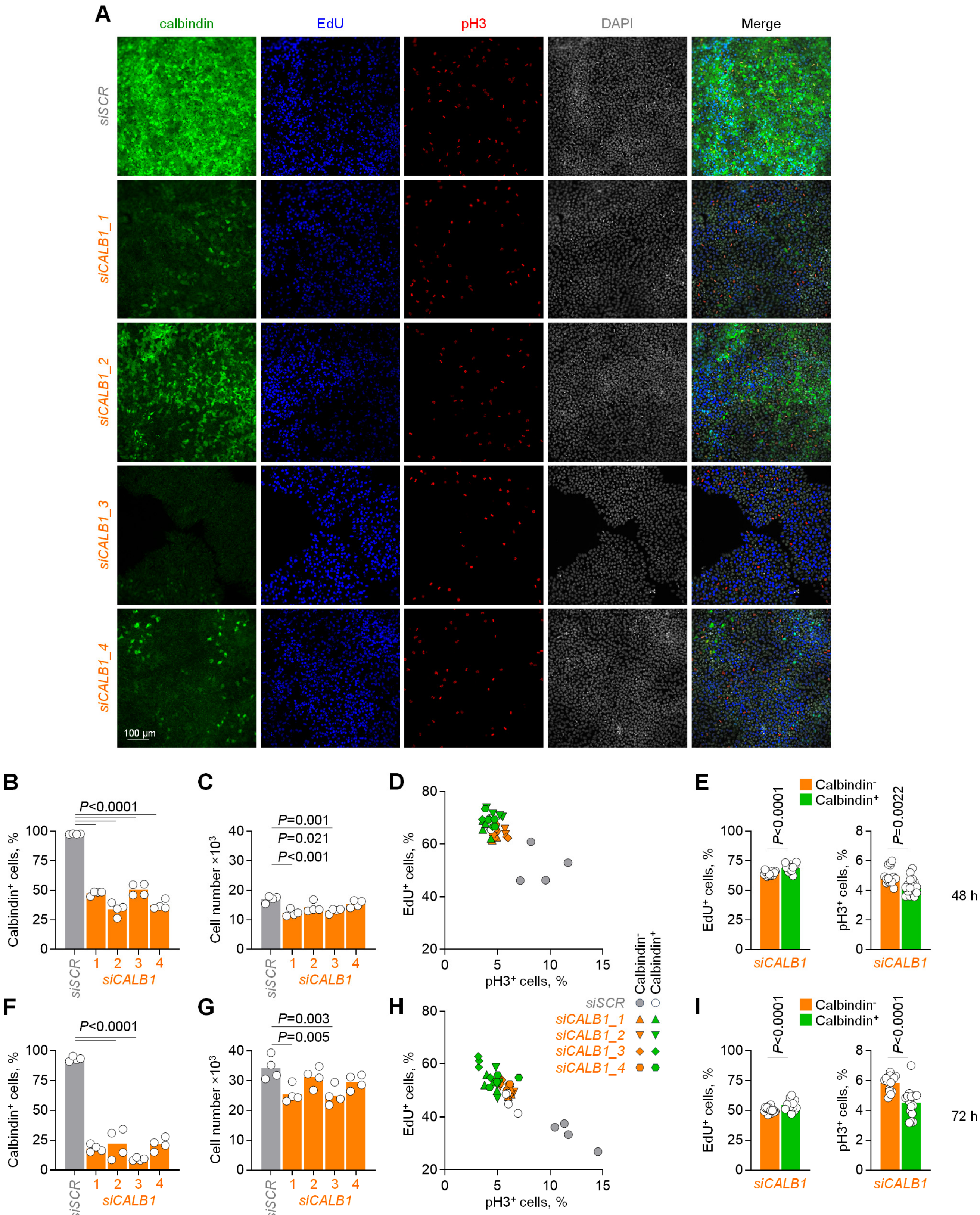

A
calbindin
EdU
pH3
DAPI
Merge
siSCR
siCALB1_1
siCALB1_2
siCALB1_3
siCALB1_4
100 µm
B
P<0.0001
Calbindin+ cells, %
C
P=0.001
P=0.021
P<0.001
Cell number ×10³
D
EdU+ cells, %
pH3+ cells, %
E
Calbindin-
Calbindin+
P<0.0001
P=0.0022
EdU+ cells, %
pH3+ cells, %
48 h
F
P<0.0001
Calbindin+ cells, %
G
P=0.003
P=0.005
Cell number ×10³
H
Calbindin-
Calbindin+
siSCR
siCALB1_1
siCALB1_2
siCALB1_3
siCALB1_4
EdU+ cells, %
pH3+ cells, %
I
Calbindin-
Calbindin+
P<0.0001
P<0.0001
EdU+ cells, %
pH3+ cells, %
72 h
siCALB1

Fig. S9. siRNA-mediated *CALB1* knockdown in H9 ESCs

(**A**) Immunofluorescence staining for calbindin and EdU incorporation, and nuclear DNA staining with DAPI in H9 ESCs cultures 72 h following transfection with 4 individual *CALB1*-targeting siRNAs (*siCALB1_1* to *siCALB1_4*) or a pool of non-targeting controls (*siSCR*).

(**B, F**) Quantitation of the proportion of calbindin-expressing cells 48 h (B) and 72 h (F) post transfection. P values were calculated with one-way ANOVA tests, with Dunnett's correction for multiple comparisons.

(**C, G**) Quantitation of total cell numbers 48 h (C) and 72 h (G) post transfection. P values were calculated with one-way ANOVA tests, with Dunnett's correction for multiple comparisons.

(**D, H**) Proportion of EdU^+ (S-phase) and $pH3^+$ (M-phase) cells within calbindin-expressing and non-expressing cells, 48 h (D) and 72 h (H) post transfection with *CALB1*-targeting siRNAs (*siCALB1_1* to *siCALB1_4*) or with a pool of non-targeting controls (*siSCR*).

(**E, I**) Quantitation of the proportion of EdU^+ (S-phase) or $pH3^+$ (M-phase) cells within calbindin-expressing and non-expressing cells, 48 h (E) and 72 h (I) post transfection with *CALB1*-targeting siRNAs. Data from the 4 separate *CALB1*-targeting siRNAs are combined and P values were calculated with paired Student's t-tests.

In B-I, symbols represent separately-transfected wells from a single experiment.

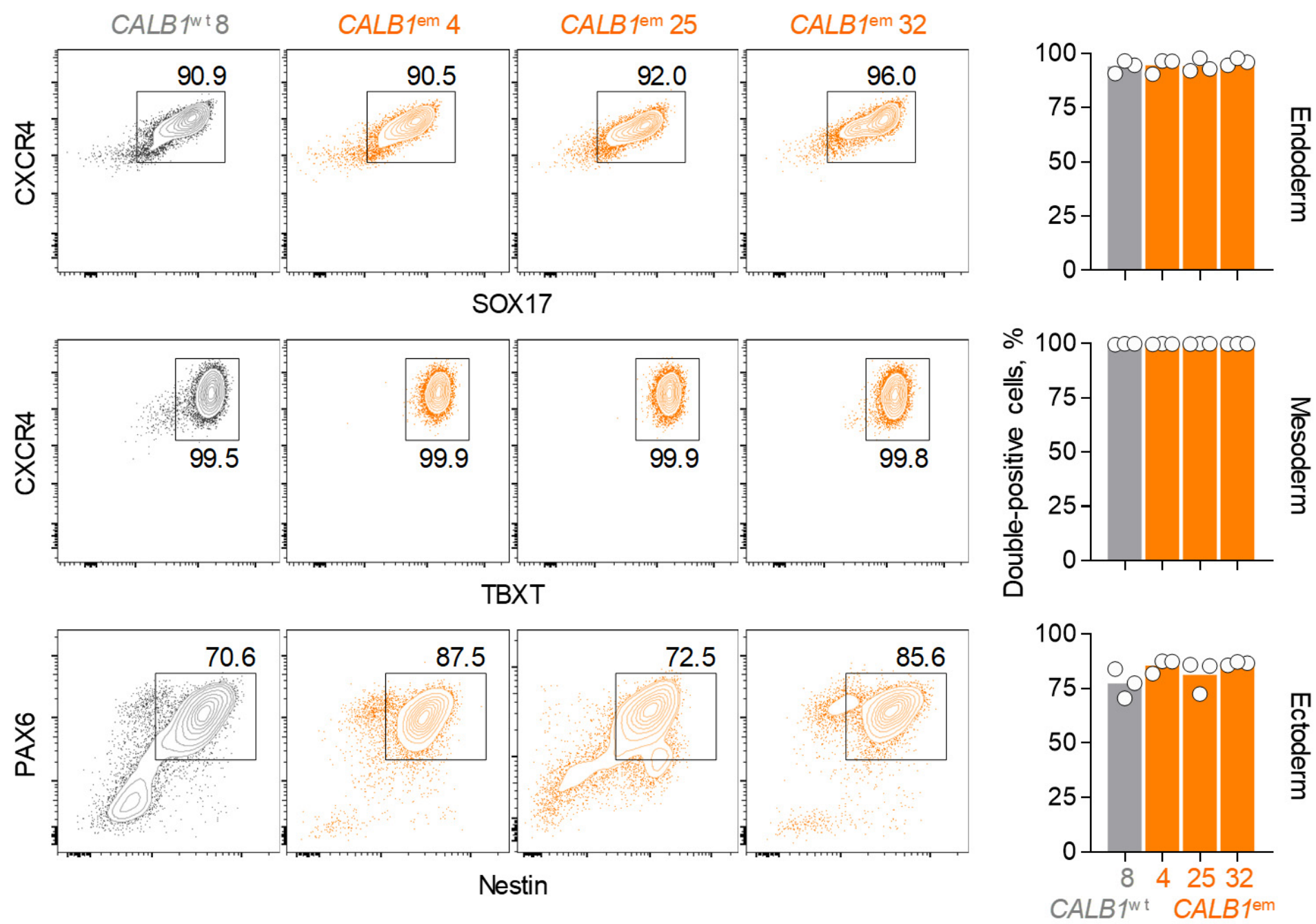

Fig. S10. Pluripotency potential in *CALB1*^{em} ESC clones

Flow cytometric detection (*left*) and quantitation of co-expression of CXCR4 and SOX17 (endoderm lineage), CXCR4 and TBXT (mesoderm lineage) or PAX6 and Nestin (ectoderm lineage) (*right*) following differentiation of *CALB1*^{wt} clone 8 and *CALB1*^{em} clones 4, 25 and 32 into the three primary germ layers. Symbols represent technical replicates from one or two experiments.

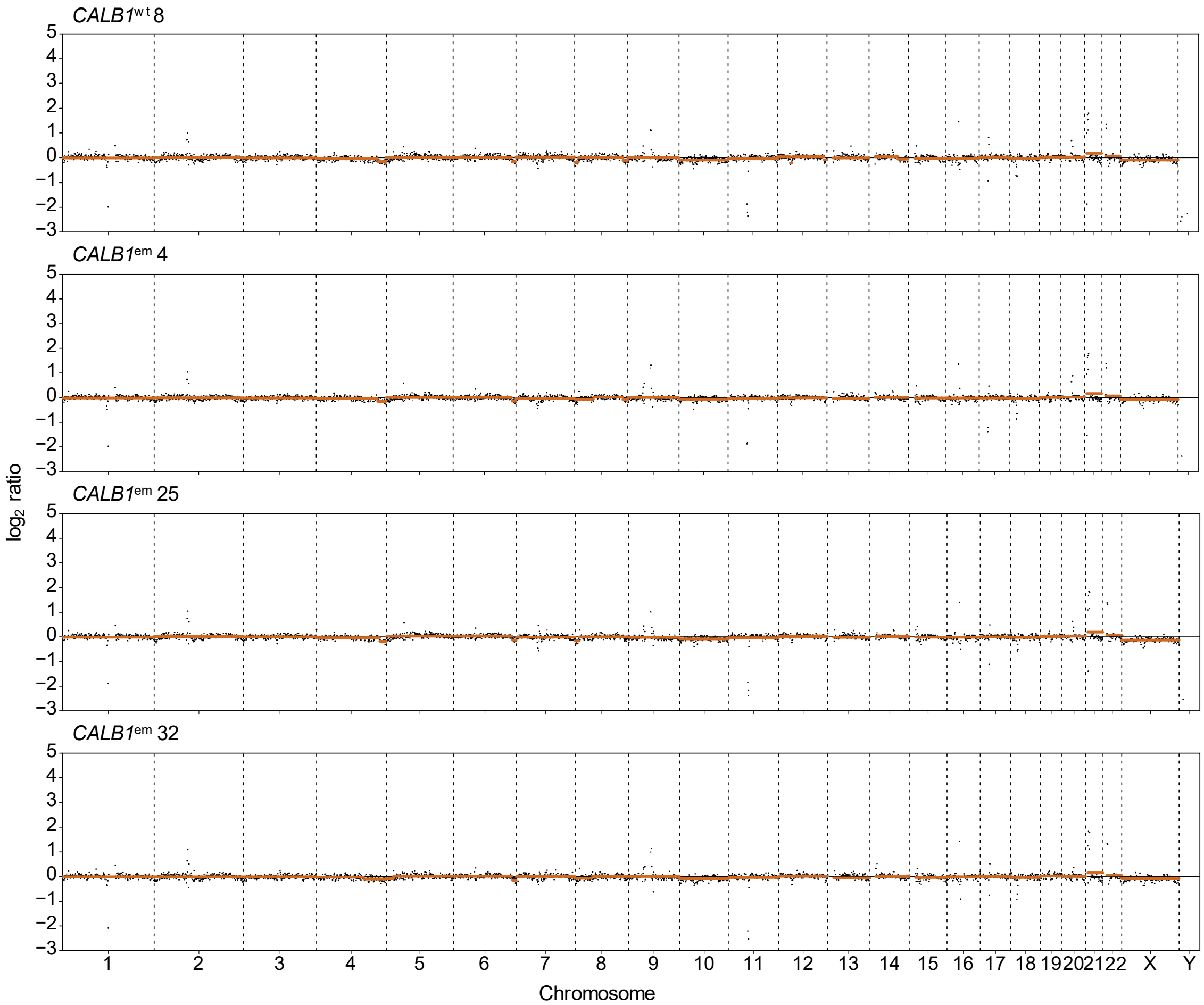

Fig. S11. Karyotypes of naive *CALB1*^em^ ESC clones

Karyotypes of the indicated chemically-reset naïve *CALB1*^{em} clones, determined by genomic DNA sequencing. The karyotype of naïve *CALB1*^{wt} clone 8 is also included for comparison.

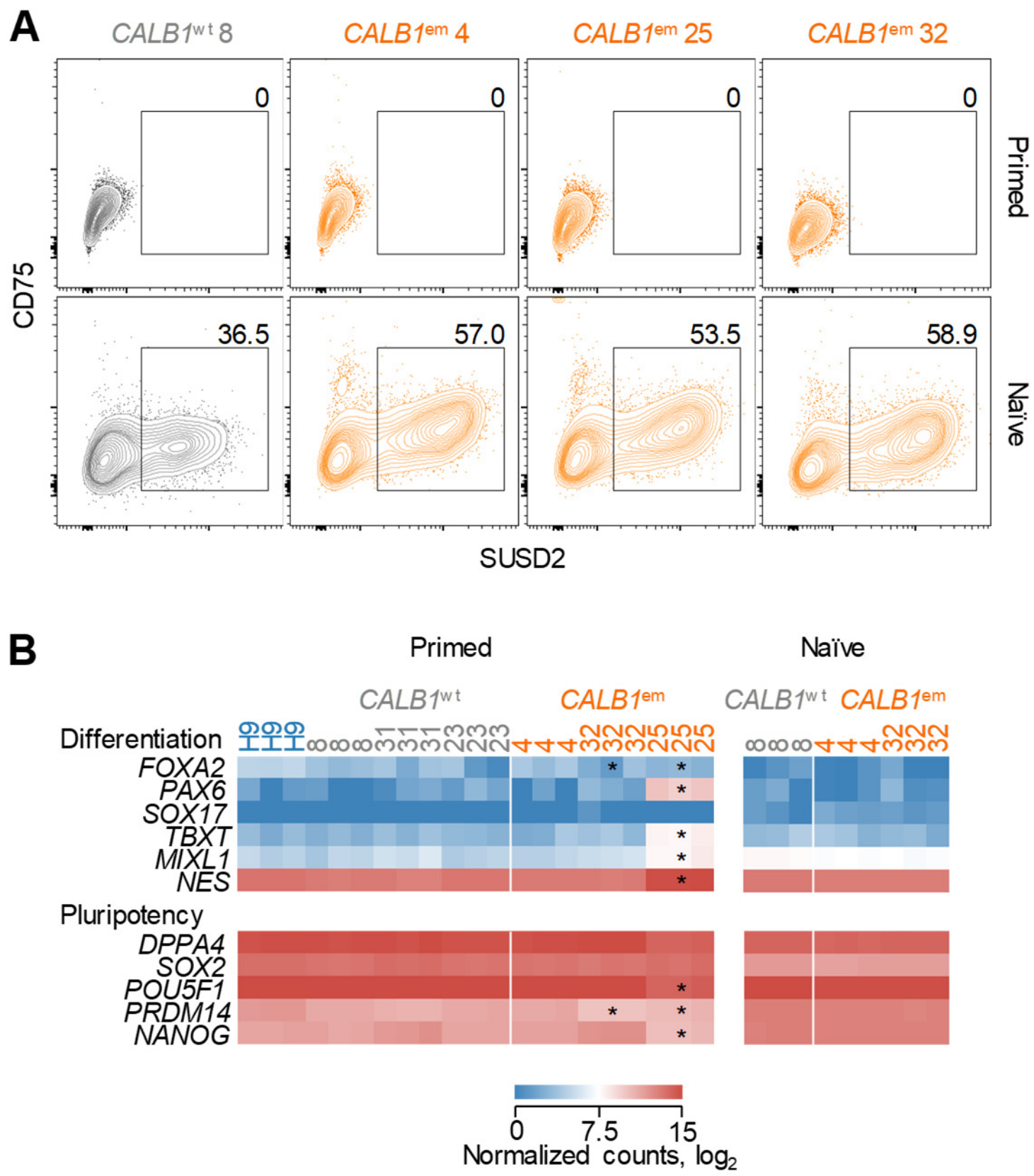

Fig. S12. Naïve state conversion potential in *CALB1*[em] ESC clones

(**A**) Flow cytometric detection of expression of the naïve ESC markers SUSD2 and CD75 in primed and chemically-reset naïve *CALB1*[wt] clone 8 and *CALB1*[em] clones 4, 25 and 32.

(**B**) Heatmap of expression of lineage differentiation genes *FOXA2*, *PAX6*, *SOX17*, *TBXT*, *MIXL1* and *NES*, and of pluripotency genes *DPPA4*, *SOX2*, *POU5F1*, *PRDM14* and *NANOG*, in the indicated primed or naïve parental H9 cells and *CALB1*[em] or *CALB1*[wt] control clones. Naïve *CALB1*[em] clone 25 failed RNA-seq quality control and is not included in this analysis. Asterisks denote significantly differential expression (≥2-fold, $p<0.05$) compared with parental H9 cells and *CALB1*[wt] control clones.

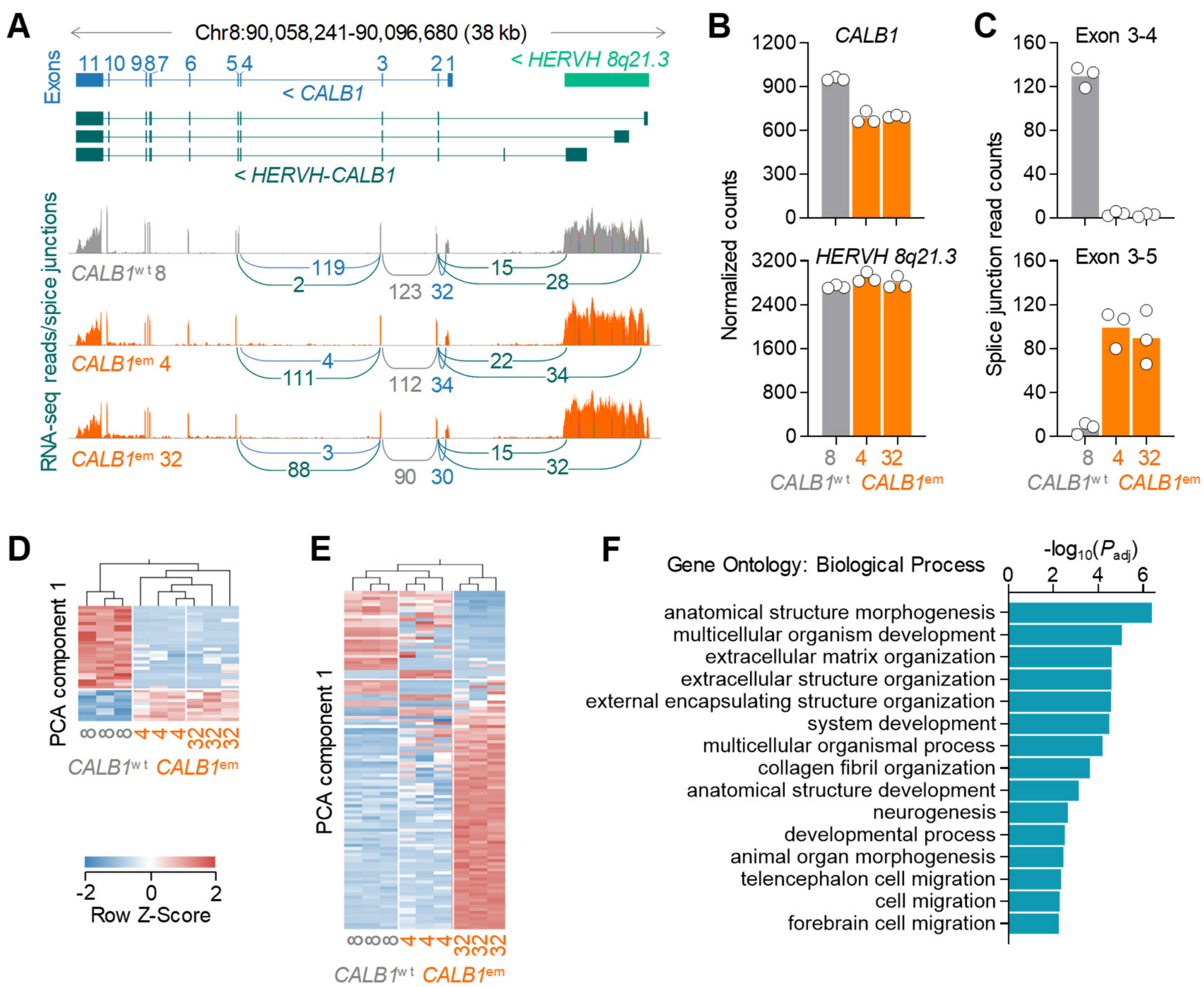

Fig. S13. Transcriptional profiling in naïve *CALB1*^{em} ESC clones

(**A**) The *CALB1* locus and integrated *HERVH* provirus; and RNA-seq traces from chemically-reset naïve *CALB1*^{wt} clone 8 and *CALB1*^{em} clones 4 and 32. Naïve *CALB1*^{em} clone 25 failed RNA-seq quality control and is not included in these analyses in (A-F).

(**B**) Quantitation of RNA-seq read counts for the *CALB1* gene (*top*) and the *HERVH 8q21.3* provirus (*bottom*) in the same clones as in (A). Symbols represent technical RNA-seq replicates.

(**C**) Quantitation of RNA-seq reads spanning the junctions of *CALB1* exon 3-4 (*top*) and 3-5 (*bottom*) in the same clones as in (A). Symbols represent technical RNA-seq replicates.

(**D**) Heatmap of 36 differentially-expressed genes (≥2-fold, $p<0.05$, $q<0.05$) between naïve *CALB1*^{wt} control clone 8 and naïve *CALB1*^{em} clones 4 and 32 (grouped together), assessed by RNA-seq. Columns represent RNA-seq technical replicates.

(**E**) Heatmap of 111 differentially-expressed genes (≥2-fold, $p<0.05$, $q<0.05$) between naïve *CALB1*^{wt} control clone 8 and either *CALB1*^{em} clone 4 or 32 (tested separately), assessed by RNA-seq. Columns represent RNA-seq technical replicates.

(**F**) Functional annotation by gene ontology (GO) of the differentially-expressed genes in *CALB1*^{em} clones 4 and 32 in E. P values were calculated with the g:SCS algorithm.

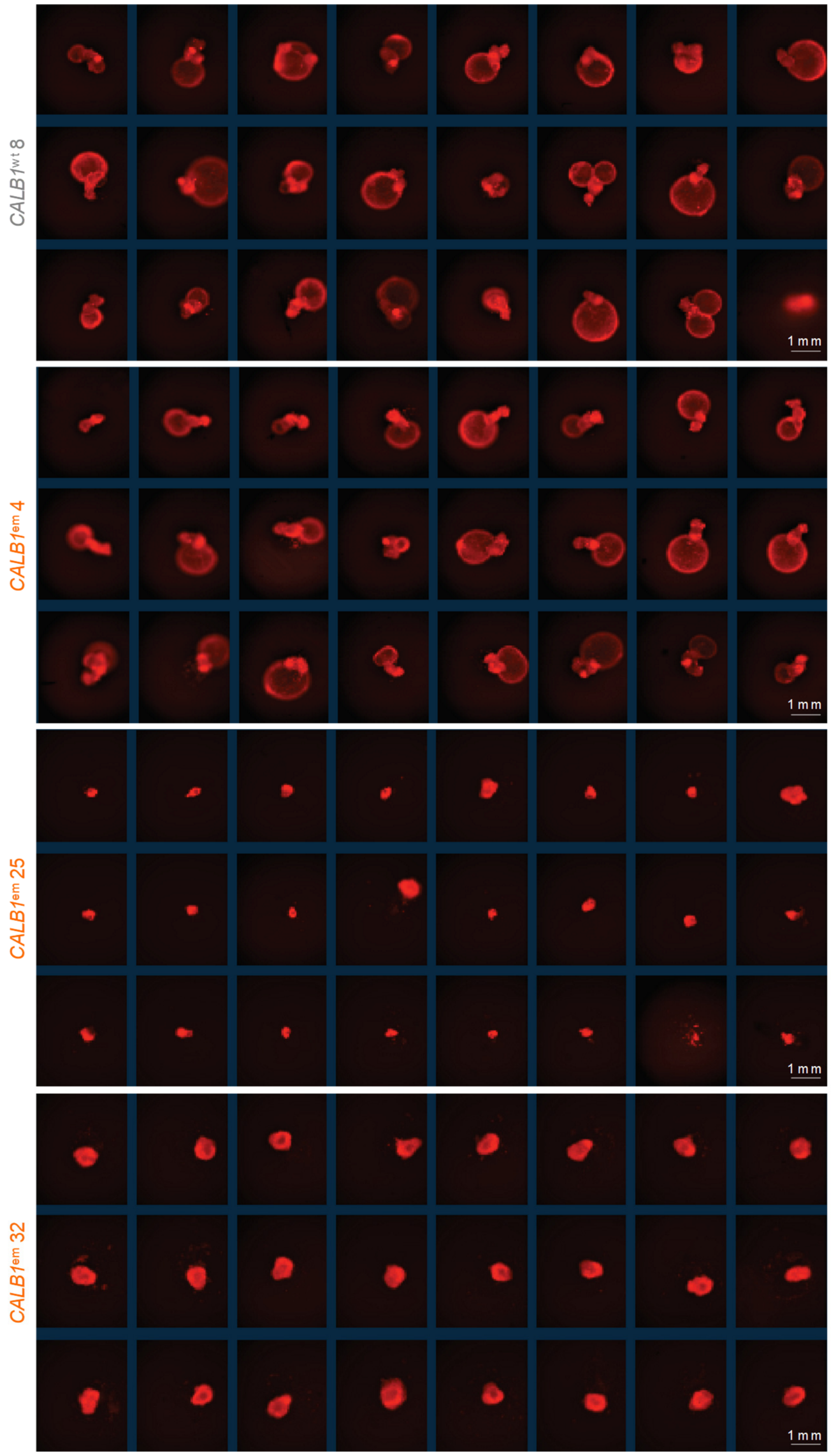

Fig. S14. PGA formation by ***CALB1*****^{em} ESC clones**

Representative images of NUCLEAR-ID Red-stained PGAs formed by the indicated *CALB1*^{em} clones on day 14. *CALB1*^{wt} clone 8 is also included for comparison.

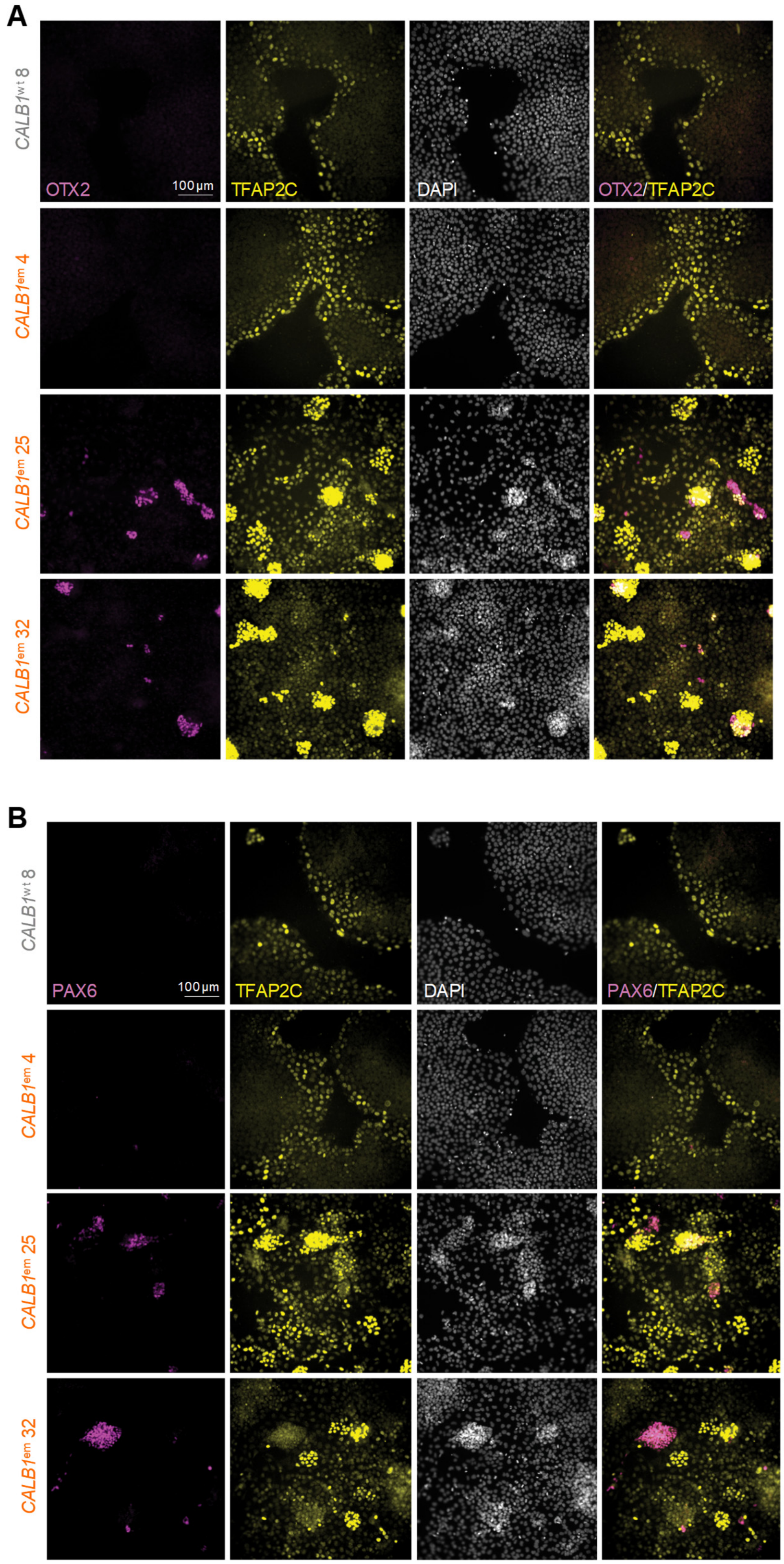
A
CALB1wt 8
CALB1em 4
CALB1em 25
CALB1em 32
OTX2
100 µm
TFAP2C
DAPI
OTX2/TFAP2C
B
CALB1wt 8
CALB1em 4
CALB1em 25
CALB1em 32
PAX6
100 µm
TFAP2C
DAPI
PAX6/TFAP2C

Fig. S15. PGA formation by *CALB1*^em^ ESC clones

(**A, B**) Immunofluorescence staining for neuroectoderm markers OTX2 (A) and PAX6 (B), together with co-staining for amniotic ectoderm marker TFAP2C and nuclear DNA staining with DAPI in initial 2D cultures of the indicated *CALB1*^{em} clones on day 2 of the PGA formation protocol. *CALB1*^{wt} clone 8 is also included for comparison.

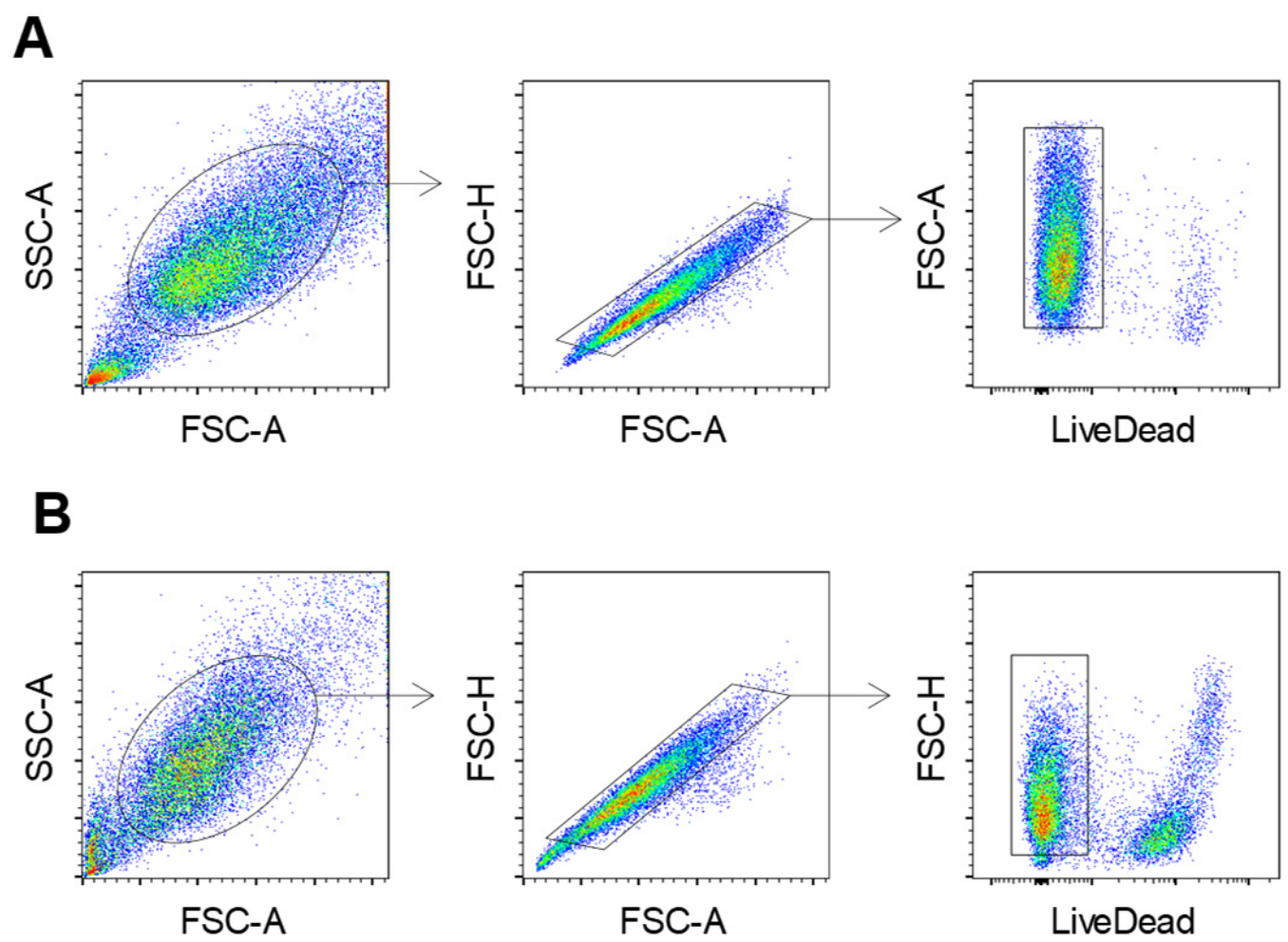

Fig. S16. Flow cytometry gating strategies

(**A**) Representative flow cytometry plots of live cell gating for the phenotyping of *CALB1*^{wt} and *CALB1*^{em} clones following differentiation into the three primary germ layers.

(**B**) Representative flow cytometry plots of live cell gating for the phenotyping of primed and chemically-reset naïve *CALB1*^{wt} and *CALB1*^{em} clones.

Table S1. Data and materials used in this study

Data and materials	Source	Identifier
Antibodies		
Rabbit anti-CALB1	Atlas	Cat# HPA023099; RRID: AB_1845859
Mouse anti-Calbindin-D28k	Proteintech	Cat# 66394-1-IG; RRID: AB_2881769
Rabbit anti-Connexin 43	Abcam	Cat# ab11370; RRID: AB_297976
Mouse anti-MAP2	Abcam	Cat# ab11267; RRID: AB_297885
Rat anti-SOX2	Invitrogen	Cat# 14-9811-82; RRID: AB_11219471
Goat anti-GATA-3	R and D Systems	Cat# AF2605; RRID: AB_2108571
Rabbit anti-GATA-3	Cell Signaling Technology	Cat# 5852; RRID: AB_10835690
Rat anti-GATA -4	Invitrogen	Cat# 14-9980-82; RRID: AB_763541
Rabbit anti-GATA-6	Cell Signaling Technology	Cat# 5851; RRID: AB_10705521
Mouse anti-TFAPC2	Abcam	Cat# ab110635; RRID: AB_ 10858471
Rabbit anti-OTX2	Abcam	Cat# ab183951; RRID: AB_3076432
Alexa Fluor® 488 anti-mouse IgG H&L	Abcam	Cat# ab150113; RRID: AB_2576208
Alexa Fluor® 594 anti-rabbit IgG H&L	Abcam	Cat# ab150080; RRID: AB_2650602
Alexa Fluor® 488 anti-rabbit IgG H&L	Abcam	Cat# ab150077; RRID: AB_2630356
Alexa Fluor® 594 anti-mouse IgG H&L	Abcam	Cat# ab150116; RRID: AB_2650601
Alexa Fluor® 594 anti-goat IgG H&L	Abcam	Cat# ab150132; RRID: AB_2810222
Alexa Fluor® 594 anti-rat IgG H&L	Abcam	Cat# ab150160; RRID: AB_2756445
Alexa Fluor® 647 anti-goat IgG H&L	Abcam	Cat# ab150131; RRID: AB_2732857
Anti-Pan-Actin	Cell Signaling Technology	Cat# 8456S; RRID: AB_10998774
Anti-rabbit IgG, HRP-linked	Cell Signaling Technology	Cat# 7074S; RRID: AB_2099233
Anti-Human CD184 (CXCR4)	STEMCELL Technologies	Cat# 60089; RRID: AB_2936358
Anti-SOX17	R and D Systems	Cat# AF1924; RRID: AB_355060
Anti-Brachyury	Abcam	Cat# ab209665; RRID: AB_2750925

Anti-PAX6	Invitrogen	Cat# 42-6600; RRID: AB_2533534
Anti-Nestin	STEMCELL Technology	Cat# 60091; RRID: AB_2905494
Anti-CD75	BioLegend	Cat# 326901; RRID: AB_2194435
Anti-SUSD2	R and D Systems	Cat# MAB90561; RRID AB_3659305
PE anti-mouse IgG H&L	Invitrogen	Cat# M3004-1; RRID: AB_2536617
Alexa Fluor™ 633 anti-rabbit IgG H&L	Invitrogen	Cat# A21070; RRID: AB_2535731
Alexa Fluor™ 633 anti-goat IgG H&L	Invitrogen	Cat# A-21082; RRID: AB_2535739
FITC anti-mouse IgG1	BD Bioscience	Cat# 553443; RRID: AB_394862
Brilliant Violet 421TM anti-mouse IgGM	BioLegend	Cat# 406517; RRID: AB_10899576
Phospho-Histone H3 (Ser10)	Cell Signalling Technology	Cat# CST9706; RRID: AB_331748
Anti-Rabbit Alexa Fluor 488	Life Technologies	Cat# A21206; RRID: AB_2535792
Anti-Mouse Alexa Fluor 568	Life Technologies	Cat# A10037; RRID: AB_11180865
Chemicals, peptides and recombinant proteins		
Matrigel® hESC-Qualified Matrix	Corning	Cat# CLS354277
mTESR™1 Basal Medium and Supplement	STEMCELL Technologies	Cat# 85850
Versene Solution (1×)	GIBCO	Cat# 15040066
Advanced DMEM/F-12	GIBCO	Cat# 12634010
StemPro™ Accutase™ Cell Dissociation Reagent	GIBCO	Cat# A1110501
Accumax™ Solution	Sigma-Aldrich	Cat# A7089
ROCKi Y-27632 dihydrochloride	Bio-Techne	Cat# 1254
CryoStor® CS10	STEMCELL Technologies	Cat# 07930
Bovine Gelatin	Sigma-Aldrich	Cat# G1393
DMEM, high glucose, pyruvate	GIBCO	Cat# 41966029
Fetal Bovine Serum	GIBCO	Cat# A52567
DMEM/F-12	Sigma-Aldrich	Cat# D8437
Neurobasal™ Medium	GIBCO	Cat# 21103049
GlutaMAX™ Supplement	GIBCO	Cat# 35050061

N-2 Supplement (100×)	GIBCO	Cat# 17502048
B-27™ Supplement (50×)	GIBCO	Cat# 17504044
MEM Non-Essential Amino Acids Solution (100×)	GIBCO	Cat# 11140050
HEPES 1M	GIBCO	Cat# 15630080
Sodium Pyruvate (100 mM)	GIBCO	Cat# 11360070
Mirdametinib (PD0325901)	Selleck Chemicals	Cat# S1036
VPA (Valproic acid)	Selleck Chemicals	Cat# S3944
Human LIF Recombinant Protein	GIBCO	Cat# 300-05
XAV939	Cell Guidance Systems	Cat# SM38
Go6983	Bio-Techne	Cat# 2285
Anti-Adherence Rinsing Solution	STEMCELL Technologies	Cat# 07010
TrypLE™ Select Enzyme (1×), no phenol red	GIBO	Cat# 12563011
A 83-01	MedChemExpress	Cat# HY-10432
1-Oleoyl lysophosphatidic acid sodium salt	Bio-Techne	Cat# 3854
NucBlue™ Fixed Cell ReadyProbes™ Reagent (DAPI)	Invitrogen	Cat# R37606
AlexaFluor™ 568 Phalloidin Labeling Probes	Invitrogen	Cat# A12380
Paraformaldehyde, 4% in PBS	Thermo Scientific	Cat# J61899.AK
Bovine Serum Albumin	Bio-Techne	Cat# 5217
DPBS, no calcium, no magnesium	GIBCO	Cat# 14190094
Triton™ X-100	Sigma-Aldrich	Cat# X100
TWEEN® 20	Sigma-Aldrich	Cat# P9416
Normal Donkey Serum	Abcam	Cat# ab7475
VECTASHIELD® Antifade Mounting Medium with DAPI	Vector Labs	Cat# H-1200
RIPA Buffer	Sigma-Aldrich	Cat# R0278
Sample Buffer, Laemmli 2×Concentrate	Sigma-Aldrich	Cat# S3401
PageRuler™ Plus Prestained Protein Ladder, 10 to 250 kDa	Thermo Scientific	Cat# 26619

Clarity™ Western ECL Substrate	BIO-RAD	Cat# 1705060
eBioscience™ Fixation/Permeabilization Diluent	Invitrogen	Cat# 00-5223-56
eBioscience™ Permeabilization Buffer (10×)	Invitrogen	Cat# 00-8333-56
eBioscience™ Fixation/Permeabilization Concentrate	Invitrogen	Cat# 00-5123-43
Sodium azide	Sigma-Aldrich	Cat# S2002
Puromycin dihydrochloride	Sigma-Aldrich	Cat# P9620
Recombinant human BMP4	Life Technologies	Cat# PHC9534
CHIR99021 (CHIRON)	Sigma-Aldrich	Cat# SML1046
Nutristem hPSC XF	SARTORIUS	Cat# 01-0005
Gentle Cell Dissociation Reagent	STEMCELL Technologies	Cat# 100-0485
TeSR™- E6	STEMCELL Technologies	Cat# 5946
GLOBAL®	LifeGobal	Cat# LGGG-20
Protein Supplement	LifeGlobal	Cat# LGPS-605
Mineral oil	Origio	Cat# ART-4008-5P
5-ethynyl-2'-deoxyuridine (EdU)	Thermo Scientific	Cat# A10044
Critical Commercial Assays		
RNeasy Mini Kit	QIAGEN	Cat# 74106
RNeasy Micro Kit	QIAGEN	Cat# 74004
RecoverAll™ Total Nucleic Acid Isolation Kit for FFPE	Invitrogen	Cat# AM1975
High-Capacity cDNA Reverse Transcription Kit	Applied Biosystems	Cat# 4368814
Fast SYBR™ Green Master Mix	Applied Biosystems	Cat# 4385617
DNeasy Blood & Tissue Kit	QIAGEN	Cat# 69504
KOD Hot Start Master Mix	Sigma-Aldrich	Cat# 71842
PrimeSTAR® GXL Premix	Takara	Cat# R051A
Beckman Coulter™ Agencourt AMPure XP	Fisher Scientific	Cat# 10136224
CellEvent™ Senescence Green Detection Kit	Invitrogen	Cat# C10850

CellTiter-Glo® 2.0 Cell Viability Assay	Promega	Cat# G9241
Pierce^{TM} BCA Protein Assay Kit	Thermo Scientific	Cat# 23227
STEMdiff^{TM} Trilineage Differentiation Kit	STEMCELL Technologies	Cat# 05230
P3 Primary Cell 4D-Nucleofector® X Kit L	Lonza	Cat# V4XP3012
Lipofectamine™ 3000 Transfection Reagent	Thermo Scientific	Cat# L3000001
Lipofectamine™ RNAiMAX Transfection Reagent	Thermo Scientific	Cat# 13778150
Vit Kit-Thaw	Fujifilm Biosciences	Cat# 90137-SO
LIVE/DEAD™ Fixable Near-IR Dead Cell Stain Kit	Thermo Scientific	Cat# L34976
NUCLEAR-IDR® Red DNA stain	Enzo Biochem	Cat# ENZ-52406
Click-iT™ EdU Cell Proliferation Kit for Imaging, Alexa Fluor™ 647 dye	Thermo Scientific	Cat# C10340
Deposited data		
RNA-Seq from primed *CALB1*^{wt} and *CALB1*^{em} H9 ESC clones	This study	https://www.ebi.ac.uk/arrayexpress/E-MTAB-15900
RNA-Seq from naïve *CALB1*^{wt} and *CALB1*^{em} H9 ESC clones	This study	https://www.ebi.ac.uk/arrayexpress/E-MTAB-16688
Single-cell RNA-Seq from primed *CALB1*^{wt} and *CALB1*^{em} H9 ESC clones	This study	https://www.ebi.ac.uk/arrayexpress/E-MTAB-16802
Single-cell RNA-Seq of human preimplantation embryos and ESCs	(*26*)	https://www.ncbi.nlm.nih.gov/geo/query/acc.cgi?acc=GSE36552
RNA-Seq of human preimplantation embryos and ESCs	(*27*)	https://www.ncbi.nlm.nih.gov/geo/query/acc.cgi?acc=GSE101571
Single-cell RNA-Seq of human and non-human primate preimplantation embryos	(*32*)	http://petropoulos-lanner-labs.clintec.ki.se
ChIP-seq of primed and naïve human ESCs	(*31*)	http://www.ncbi.nlm.nih.gov/geo/query/acc.cgi?acc=GSE99631
Single-cell RNA-Seq of mouse pig preimplantation embryos	(*66*)	http://bioinfor.imu.edu.cn/ematlas
Single-cell RNA-Seq of Guinea pig preimplantation embryos	(*67*)	http://petropoulos-lanner-labs.clintec.ki.se
Lung squamous cell carcinoma RNA-seq	Cancer Genome Atlas Program (TCGA)	http://cancergenome.nih.gov
RNA-seq of human cancer cell lines	Cancer Cell Line Encyclopedia (CCLE)	https://portals.broadinstitute.org/ccle/data
Experimental models: cell lines		
H9 hESCs	WiCell	Cat# WA09
DR4 mouse embryonic fibroblast feeder cells, irradiated	In house biological resources unit	N/A

DR4 mouse embryonic fibroblast feeder cells, irradiated, puroR	Afshan McCarthy	N/A
Oligonucleotides		
Full length *CALB1* F CCACCTGCAGTCATCCCTC	(*22*)	N/A
Full length *CALB1* R ATCAAGTTCTGCAGCTCCTTTCC	(*22*)	N/A
HERVH-CALB1 F AGCCCAAGAAACATCTCACCAA	(*22*)	N/A
HERVH-CALB1 R CAGCCTTCTTTCGCGCCTG	(*22*)	N/A
HPRT1 F TGACACTGGCAAAACAATGCA	(*22*)	N/A
HPRT1 R GGTCCTTTTCACCAGCAAGCT	(*22*)	N/A
Human *HPRT1*-specific F CATTATGCTGAGGATTTGGAAAGG	This study	N/A
Human *HPRT1*-specific R CTTGAGCACACAGAGGGCTACA	This study	N/A
sgRNA human *CALB1* no.16 GCTGGCATCGGAAGAGCAGC	(*22*)	N/A
sgRNA human *CALB1* no. 19 GTCCTGTGAGGAATTCATGA	(*22*)	N/A
sgRNA human *HERVH 8q21.3* no. 1 GAATTTTCAAGAATTGTTAAT	(*22*)	N/A
sgRNA human *HERVH 8q21.3* no. 2 GCCATTGTAGTCGATGTGTAG	(*22*)	N/A
sgRNA human *HERVH 8q21.3* no. 3 GAGTCAATCAGATGCCTACTT	(*22*)	N/A
sgRNA human *HERVH 8q21.3* no. 4 GTTAAATCTAAATGTATCTTA	(*22*)	N/A
sgRNA human *HERVH 8q21.3* no. 5 GCAAATGTTCTAATTTATCC	(*22*)	N/A
siCALB1_1 siRNA GCACAUUUGUCACUGAAUA	ON-TARGETplus, Horizon Discovery	Cat# J2-011989-01-0002
siCALB1_2 siRNA CUGAGGAGCUUAAGAACUU	ON-TARGETplus, Horizon Discovery	Cat# J2-011989-02-0002
siCALB1_3 siRNA CCAUAGAACUGUCCCACAA	ON-TARGETplus, Horizon Discovery	Cat# J2-011989-03-0002
siCALB1_4 siRNA GGACGGCAAUGGAUACAUA	ON-TARGETplus, Horizon Discovery	Cat# J2-011989-04-0002
siSCR, non-targeting control pool UGGUUUACAUGUCGACUAA UGGUUUACAUGUUGUGUGA UGGUUUACAUGUUUUCUGA UGGUUUACAUGUUUUCCUA	ON-TARGETplus, Horizon Discovery	Cat# D-001810-10-05
Recombinant DNA		

pSpCas9(BB)-2A-Puro (PX459) V2.0	(*68*)	Addgene #62988; RRID: Addgene_62988
pmaxGFP™	Lonza	Cat# V4XP3012
Software and algorithms		
GraphPad Prism 10.6.0	Graphpad Software	https://www.graphpad.com
SigmaPlot 14.0	Grafiti LLC	https://grafiti.com/
scanR Acquisition Software 3.4.1	Evident Scientific	https://evidentscientific.com
Fiji ImageJ	Open source	https://fiji.sc
FlowJo 10	Flow Jo Software	https://www.flowjo.com
Qlucore Omics Explorer	Qlucore Software	https://qlucore.com
g:Profiler	(*61*)	https://biit.cs.ut.ee/gprofiler/gost
Integrative Genomics Viewer 2.19.6	(*60*)	https://igv.org
Ensembl Comparative Genomics tool	https://www.ensembl.org/info/genome/compara/index.html	NA
UCSC Genome Browser	https://genome.ucsc.edu	NA
AlphaFold	AlphaFold Server	https://alphafold.ebi.ac.uk/
GNU Parallel	(*69*)	https://www.gnu.org/software/parallel
pigz	GitHub	https://github.com/madler/pigz
Trimmomatic v.0.36	(*70*)	http://www.usadellab.org/cms/?page=trimmomatic
FastQC	https://www.bioinformatics.babraham.ac.uk/projects/fastqc	NA
bedtools package	(*71*)	http://bedtools.readthedocs.io
Hisat2 v.2.1.0	(*72*)	http://daehwankimlab.github.io/hisat2/
SAMtools	(*73*)	https://github.com/samtools/samtools
Subread package v.1.5.0	(*74*)	http://subread.sourceforge.net/
R v.4.2.0	R Studio Software	http://www.r-project.org
R package "DESeq2"	(*75*)	https://bioconductor.org/packages/release/bioc/html/DESeq2.html

R package "data.table"	https://rdatatable.gitlab.io /data.table	NA
R package "S4Vectors"	https://www.bioconductor.org /packages/S4Vectors	NA
R package "dplyr"	https://dplyr.tidyverse.org/	NA
Other		
μ-Slide 8 Well high, uncoated	ibidi	Cat# 80801
AggreWell™400, 24-well	STEMCELL Technologies	Cat# 34415
The Stripper™ Pipettor	Cooper Surgical	Cat# MXL3-STR
4–20% Mini-PROTEAN® TGX™ Precast Protein Gels, 10-well	BIO-RAD	Cat# 4561094
Trans-Blot Turbo Mini 0.2 μm Nitrocellulose Transfer Packs	BIO-RAD	Cat# 1704158
Cell Repellent-Treated U-Shaped-Bottom CELLSTAR® 96-well Microplate	Greiner Bio-One	Cat# 650970
EmbryoSlide+ culture dishes	Vitrolife	Cat# 16540

Movies S1 to S10.

Movies of human blastocyst developing from primary embryos. Movie files correspond to the embryos analyses in Fig. 1C as follows:

Movie	Embryo number
Movie S1	1
Movie S2	2
Movie S3	3
Movie S4	4
Movie S5	6
Movie S6	9
Movie S7	10
Movie S8	11
Movie S9	12
Movie S10	15

Data file S1.

List of genes differentially expressed in primed *CALB1*^{em} clones compared with primed parental H9 cells and *CALB1*^{wt} clones.

Data file S2.

List of genes differentially expressed in either naïve *CALB1*^{em} clone 4 or 32 compared with naïve *CALB1*^{wt} clone 8.

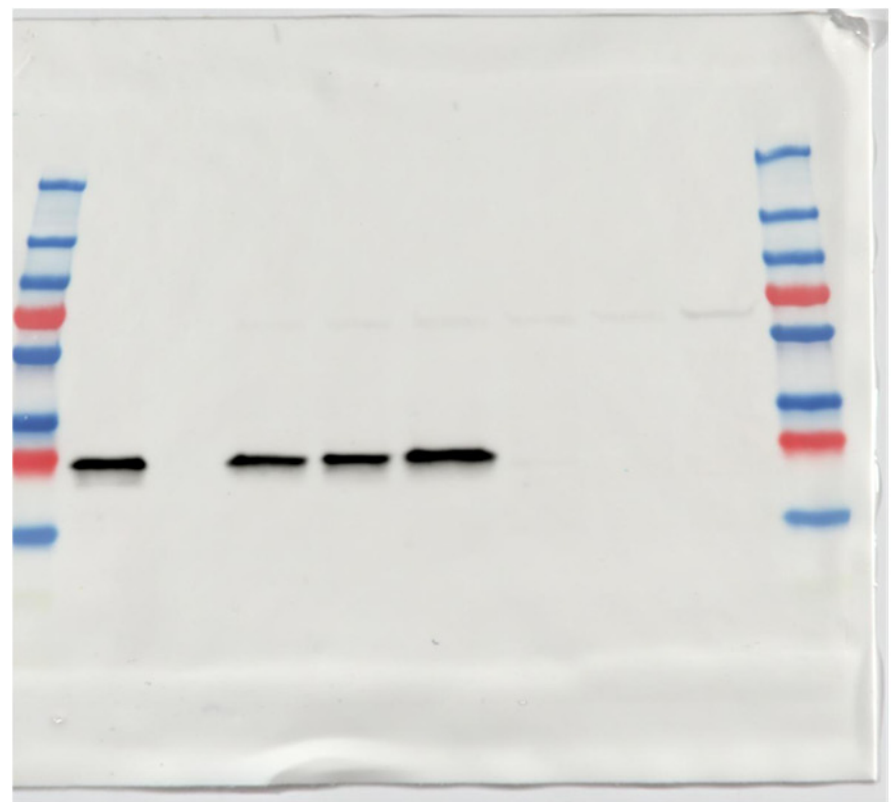

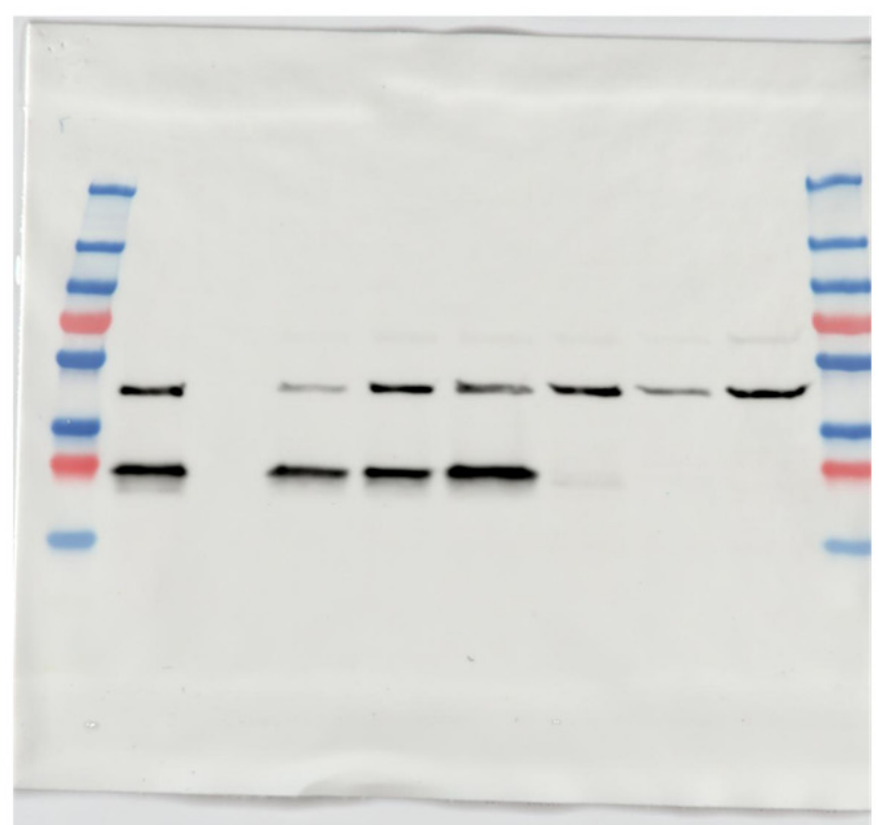

Uncropped Western blots

Unmodified and uncropped versions of Western blot gels for calbindin (top) and actin (bottom), relating to Fig. 3A.

REFERENCES

1. N. Bannert, R. Kurth, The evolutionary dynamics of human endogenous retroviral families. *Annu. Rev. Genomics Hum. Genet.* **7**, 149–173 (2006).

2. G. Kassiotis, J. P. Stoye, Evolution of antiviral host defenses against a backdrop of endogenous retroelements. *Science* **389**, 588–593 (2025).

3. C. Feschotte, C. Gilbert, Endogenous viruses: Insights into viral evolution and impact on host biology. *Nat. Rev. Genet.* **13**, 283–296 (2012).

4. K. H. Burns, J. D. Boeke, Human transposon tectonics. *Cell* **149**, 740–752 (2012).

5. G. Kassiotis, J. P. Stoye, Immune responses to endogenous retroelements: Taking the bad with the good. *Nat. Rev. Immunol.* **16**, 207–219 (2016).

6. R. Fueyo, J. Judd, C. Feschotte, J. Wysocka, Roles of transposable elements in the regulation of mammalian transcription. *Nat. Rev. Mol. Cell Biol.* **23**, 481–497 (2022).

7. Z. Izsvák, J. Ma, M. Singh, L. D. Hurst, Co-option of an endogenous retrovirus (LTR7-HERVH) in early human embryogenesis: Becoming useful and going unnoticed. *Mob DNA* **16**, 27 (2025).

8. J. Göke, X. Lu, Y. S. Chan, H. H. Ng, L. H. Ly, F. Sachs, I. Szczerbinska, Dynamic transcription of distinct classes of endogenous retroviral elements marks specific populations of early human embryonic cells. *Cell Stem Cell* **16**, 135–141 (2015).

9. T. A. Carter, M. Singh, G. Dumbović, J. D. Chobirko, J. L. Rinn, C. Feschotte, Mosaic cis-regulatory evolution drives transcriptional partitioning of HERVH endogenous retrovirus in the human embryo. *eLife* **11**, e76257 (2022).

10. Z. Ai, X. Xiang, Y. Xiang, I. Szczerbinska, Y. Qian, X. Xu, C. Ma, Y. Su, B. Gao, H. Shen, M. N. Bin Ramli, D. Chen, Y. Liu, J.-j. Hao, H. H. Ng, D. Zhang, Y.-S. Chan, W. Liu, H. Liang, Kruppel-like factor 5 rewires NANOG regulatory network to activate human naive pluripotency specific LTR7Ys and promote naive pluripotency. *Cell Rep.* **40**, 111240 (2022).

11. M. Singh, A. M. Kondrashkina, T. J. Widmann, J. L. Cortes, V. Bansal, J. Wang, C. Römer, M. Garcia-Canadas, J. L. Garcia-Perez, L. D. Hurst, Z. Izsvák, A new human embryonic cell type associated with activity of young transposable elements allows definition of the inner cell mass. *PLOS Biol.* **21**, e3002162 (2023).

12. C. Lavialle, G. Cornelis, A. Dupressoir, C. Esnault, O. Heidmann, C. Vernochet, T. Heidmann, Paleovirology of 'syncytins', retroviral env genes exapted for a role in placentation. *Philos. Trans. R. Soc. Lond. B Biol. Sci.* **368**, 20120507 (2013).

13. J. Wang, G. Xie, M. Singh, A. T. Ghanbarian, T. Rasko, A. Szvetnik, H. Cai, D. Besser, A. Prigione, N. V. Fuchs, G. G. Schumann, W. Chen, M. C. Lorincz, Z. Ivics, L. D. Hurst, Z. Izsvak, Primate-specific endogenous retrovirus-driven transcription defines naive-like stem cells. *Nature* **516**, 405–409 (2014).

14. X. Lu, F. Sachs, L. Ramsay, P. Jacques, J. Göke, G. Bourque, H. H. Ng, The retrovirus HERVH is a long noncoding RNA required for human embryonic stem cell identity. *Nat. Struct. Mol. Biol.* **21**, 423–425 (2014).

15. Y. Zhang, T. Li, S. Preissl, M. L. Amaral, J. D. Grinstein, E. N. Farah, E. Destici, Y. Qiu, R. Hu, A. Y. Lee, S. Chee, K. Ma, Z. Ye, Q. Zhu, H. Huang, R. Fang, L. Yu, J. C. Izpisua Belmonte, J. Wu, S. M. Evans, N. C. Chi, B. Ren, Transcriptionally active HERV-H retrotransposons demarcate topologically associating domains in human pluripotent stem cells. *Nat. Genet.* **51**, 1380–1388 (2019).

16. C. E. Sexton, R. L. Tillett, M. V. Han, The essential but enigmatic regulatory role of HERVH in pluripotency. *Trends Genet.* **38**, 12–21 (2022).

17. A. Babaian, D. L. Mager, Endogenous retroviral promoter exaptation in human cancer. *Mobile DNA* **7**, 24 (2016).

18. G. Kassiotis, J. P. Stoye, Making a virtue of necessity: The pleiotropic role of human endogenous retroviruses in cancer. *Philos. Trans. R. Soc. Lond. B Biol. Sci.* **372**, 20160277 (2017).

19. T. Wiesner, W. Lee, A. C. Obenauf, L. Ran, R. Murali, Q. F. Zhang, E. W. Wong, W. Hu, S. N. Scott, R. H. Shah, I. Landa, J. Button, N. Lailler, A. Sboner, D. Gao, D. A. Murphy, Z. Cao, S. Shukla, T. J. Hollmann, L. Wang, L. Borsu, T. Merghoub, G. K. Schwartz, M. A. Postow, C. E. Ariyan, J. A. Fagin, D. Zheng, M. Ladanyi, K. J. Busam, M. F. Berger, Y. Chen, P. Chi, Alternative transcription initiation leads to expression of a novel ALK isoform in cancer. *Nature* **526**, 453–457 (2015).

20. B. Lamprecht, K. Walter, S. Kreher, R. Kumar, M. Hummel, D. Lenze, K. Köchert, M. A. Bouhlel, J. Richter, E. Soler, R. Stadhouders, K. Jöhrens, K. D. Wurster, D. F. Callen, M. F. Harte, M. Giefing, R. Barlow, H. Stein, I. Anagnostopoulos, M. Janz, P. N. Cockerill, R. Siebert, B. Dörken, C. Bonifer, S. Mathas, Derepression of an endogenous long terminal repeat activates the CSF1R proto-oncogene in human lymphoma. *Nat. Med.* **16**, 571–579 (2010).

21. A. Babaian, M. T. Romanish, L. Gagnier, L. Y. Kuo, M. M. Karimi, C. Steidl, D. L. Mager, Onco-exaptation of an endogenous retroviral LTR drives IRF5 expression in Hodgkin lymphoma. *Oncogene* **35**, 2542–2546 (2016).

22. J. Attig, J. Pape, L. Doglio, A. Kazachenka, E. Ottina, G. R. Young, K. S. Enfield, I. V. Aramburu, K. W. Ng, N. Faulkner, W. Bolland, V. Papayannopoulos, C. Swanton, G. Kassiotis, Human endogenous retrovirus onco-exaptation counters cancer cell senescence through calbindin. *J. Clin. Invest.* **133**, e164397 (2023).

23. E. M. Jung, K. C. Choi, E. B. Jeung, Expression of calbindin-D28k is inversely correlated with proapototic gene expression in hydrogen peroxide-induced cell death in endometrial cancer cells. *Int. J. Oncol.* **38**, 1059–1066 (2011).

24. E. M. Jung, B. S. An, K. C. Choi, E. B. Jeung, Apoptosis- and endoplasmic reticulum stress-related genes were regulated by estrogen and progesterone in the uteri of calbindin-D(9k) and -D(28k) knockout mice. *J. Cell. Biochem.* **113**, 194–203 (2012).

25. L. Q. Cao, Y. N. Wang, M. Liang, M. Z. Pan, CALB1 enhances the interaction between p53 and MDM2, and inhibits the senescence of ovarian cancer cells. *Mol. Med. Rep.* **19**, 5097–5104 (2019).

26. L. Yan, M. Yang, H. Guo, L. Yang, J. Wu, R. Li, P. Liu, Y. Lian, X. Zheng, J. Yan, J. Huang, M. Li, X. Wu, L. Wen, K. Lao, R. Li, J. Qiao, F. Tang, Single-cell RNA-Seq profiling of human preimplantation embryos and embryonic stem cells. *Nat. Struct. Mol. Biol.* **20**, 1131–1139 (2013).

27. J. Wu, J. Xu, B. Liu, G. Yao, P. Wang, Z. Lin, B. Huang, X. Wang, T. Li, S. Shi, N. Zhang, F. Duan, J. Ming, X. Zhang, W. Niu, W. Song, H. Jin, Y. Guo, S. Dai, L. Hu, L. Fang, Q. Wang, Y. Li, W. Li, J. Na, W. Xie, Y. Sun, Chromatin analysis in human early development reveals epigenetic transition during ZGA. *Nature* **557**, 256–260 (2018).

28. G. Guo, F. von Meyenn, M. Rostovskaya, J. Clarke, S. Dietmann, D. Baker, A. Sahakyan, S. Myers, P. Bertone, W. Reik, K. Plath, A. Smith, Epigenetic resetting of human pluripotency. *Development* **144**, 2748–2763 (2017).

29. H. Kagawa, A. Javali, H. H. Khoei, T. M. Sommer, G. Sestini, M. Novatchkova, Y. Scholte Op Reimer, G. Castel, A. Bruneau, N. Maenhoudt, J. Lammers, S. Loubersac, T. Freour, H. Vankelecom, L. David, N. Rivron, Human blastoids model blastocyst development and implantation. *Nature* **601**, 600–605 (2022).

30. H. Heidari Khoei, A. Javali, H. Kagawa, T. M. Sommer, G. Sestini, L. David, J. Slovakova, M. Novatchkova, Y. Scholte Op Reimer, N. Rivron, Generating human blastoids modeling blastocyst-stage embryos and implantation. *Nat. Protoc.* **18**, 1584–1620 (2023).

31. T. S. Barakat, F. Halbritter, M. Zhang, A. F. Rendeiro, E. Perenthaler, C. Bock, I. Chambers, Functional dissection of the enhancer repertoire in human embryonic stem cells. *Cell Stem Cell* **23**, 276–288.e8 (2018).

32. C. Zhao, A. Plaza Reyes, J. P. Schell, J. Weltner, N. M. Ortega, Y. Zheng, Å. K. Björklund, L. Baqué-Vidal, J. Sokka, R. Trokovic, B. Cox, J. Rossant, J. Fu, S. Petropoulos, F. Lanner, A comprehensive human embryo reference tool using single-cell RNA-sequencing data. *Nat. Methods* **22**, 193–206 (2025).

33. B. Gharibi, O. C. K. Inge, I. Rodriguez-Hernandez, P. C. Driscoll, C. Dubois, M. Jiang, M. Howell, J. M. Skehel, J. I. Macrae, S. D. M. Santos, Post-gastrulation amnioids as a stem

cell-derived model of human extra-embryonic development. *Cell* **188**, 3757–3774.e20 (2025).

34. E. B. Chuong, N. C. Elde, C. Feschotte, Regulatory activities of transposable elements: From conflicts to benefits. *Nat. Rev. Genet.* **18**, 71–86 (2017).

35. A. J. Modzelewski, J. Gan Chong, T. Wang, L. He, Mammalian genome innovation through transposon domestication. *Nat. Cell Biol.* **24**, 1332–1340 (2022).

36. N. Dopkins, M. M. O'Mara, E. Lawrence, T. Fei, S. Sandoval-Motta, D. F. Nixon, M. L. Bendall, A field guide to endogenous retrovirus regulatory networks. *Mol. Cell* **82**, 3763–3768 (2022).

37. N. Martin, K. Zhu, J. Czarnecka-Herok, M. Vernier, D. Bernard, Regulation and role of calcium in cellular senescence. *Cell Calcium* **110**, 102701 (2023).

38. S. E. Webb, A. L. Miller, Calcium signalling during embryonic development. *Nat. Rev. Mol. Cell Biol.* **4**, 539–551 (2003).

39. D. R. Armant, Intracellular Ca^{2+} signaling and preimplantation development. *Adv. Exp. Med. Biol.* **843**, 151–171 (2015).

40. T. Berggård, S. Miron, P. Onnerfjord, E. Thulin, K. S. Akerfeldt, J. J. Enghild, M. Akke, S. Linse, Calbindin D28k exhibits properties characteristic of a Ca^{2+} sensor. *J. Biol. Chem.* **277**, 16662–16672 (2002).

41. T. Berggard, O. Szczepankiewicz, E. Thulin, S. Linse, Myo-inositol monophosphatase is an activated target of calbindin D28k. *J. Biol. Chem.* **277**, 41954–41959 (2002).

42. H. Schmidt, Three functional facets of calbindin D-28k. *Front. Mol. Neurosci.* **5**, 25 (2012).

43. B. Schwaller, The continuing disappearance of "pure" Ca^{2+} buffers. *Cell. Mol. Life Sci.* **66**, 275–300 (2009).

44. G. Kassiotis, Endogenous retroviruses and the development of cancer. *J. Immunol.* **192**, 1343–1349 (2014).

45. N. Dopkins, D. F. Nixon, Activation of human endogenous retroviruses and its physiological consequences. *Nat. Rev. Mol. Cell Biol.* **25**, 212–222 (2024).

46. C. F. Lynch-Sutherland, A. Chatterjee, P. A. Stockwell, M. R. Eccles, E. C. Macaulay, Reawakening the developmental origins of cancer through transposable elements. *Front. Oncol.* **10**, 468 (2020).

47. C. Yu, X. Lei, F. Chen, S. Mao, L. Lv, H. Liu, X. Hu, R. Wang, L. Shen, N. Zhang, Y. Meng, Y. Shen, J. Chen, P. Li, S. Huang, C. Lin, Z. Zhang, K. Yuan, ARID1A loss derepresses a group of human endogenous retrovirus-H loci to modulate BRD4-dependent transcription. *Nat. Commun.* **13**, 3501 (2022).

48. A. Kazachenka, J. H. Loong, J. Attig, G. R. Young, P. Ganguli, G. Devonshire, N. Grehan, F. D. Ciccarelli, R. C. Fitzgerald, G. Kassiotis, The transcriptional landscape of endogenous retroelements delineates esophageal adenocarcinoma subtypes. *NAR Cancer* **5**, zcad040 (2023).

49. J. Viot, R. Loyon, N. Adib, P. Laurent-Puig, A. de Reyniès, F. André, F. Monnien, T. André, M. Svrcek, A. Turpin, Z. Selmani, L. Arnould, L. Guyard, N. Gilbert, A. Boureux, O. Adotevi, A. Vienot, S. Abdeljaoued, D. Vernerey, C. Borg, D. Gautheret, Deciphering human endogenous retrovirus expression in colorectal cancers: Exploratory analysis regarding prognostic value in liver metastases. *EBioMedicine* **116**, 105727 (2025).

50. J. Loong, R. Thompson, C. Hall, L. Doglio, J. Pape, T. Plowman, G. Kassiotis, Retroelement co-option disrupts the cancer transcriptional programme. *Genome Med.* **17**, 48 (2025).

51. D. Siriwardena, T. E. Boroviak, Evolutionary divergence of embryo implantation in primates. *Philos. Trans. R. Soc. Lond. B Biol. Sci.* **377**, 20210256 (2022).

52. R. Fueyo, S. Wang, O. J. Crocker, T. Swigut, H. Nakauchi, J. Wysocka, A human-specific regulatory mechanism revealed in a pre-implantation model. *Nature* **647**, 238–247 (2025).

53. N. C. Chang, J. N. Wells, A. Y. Wang, P. Schofield, Y. C. Huang, V. H. Truong, M. Simoes-Costa, C. Feschotte, Gag proteins encoded by endogenous retroviruses are required for zebrafish development. *Proc. Natl. Acad. Sci. U.S.A.* **122**, e2411446122 (2025).

54. R. S. Tuan, E. Suyama, Developmental expression and vitamin D regulation of calbindin-D28K in chick embryonic yolk sac endoderm. *J. Nutr.* **126**, 1308s–1316s (1996).

55. Y. Han, Z. Du, H. Wu, R. Zhao, J. Liu, S. Gao, S. Zeng, CALB1 and RPL23 are essential for maintaining oocyte quality and function during aging. *Aging Cell* **24**, e14466 (2025).

56. M. S. Airaksinen, J. Eilers, O. Garaschuk, H. Thoenen, A. Konnerth, M. Meyer, Ataxia and altered dendritic calcium signaling in mice carrying a targeted null mutation of the calbindin D28k gene. *Proc. Natl. Acad. Sci. U.S.A.* **94**, 1488–1493 (1997).

57. B. Schwaller, M. Meyer, S. Schiffmann, 'New' functions for 'old' proteins: The role of the calcium-binding proteins calbindin D-28k, calretinin and parvalbumin, in cerebellar physiology. Studies with knockout mice. *Cerebellum* **1**, 241–258 (2002).

58. S. Chen, L. C. Francioli, J. K. Goodrich, R. L. Collins, M. Kanai, Q. Wang, J. Alföldi, N. A. Watts, C. Vittal, L. D. Gauthier, T. Poterba, M. W. Wilson, Y. Tarasova, W. Phu, R. Grant, M. T. Yohannes, Z. Koenig, Y. Farjoun, E. Banks, S. Donnelly, S. Gabriel, N. Gupta, S. Ferriera, C. Tolonen, S. Novod, L. Bergelson, D. Roazen, V. Ruano-Rubio, M. Covarrubias, C. Llanwarne, N. Petrillo, G. Wade, T. Jeandet, R. Munshi, K. Tibbetts, M. Abreu, C. A. Aguilar Salinas, T. Ahmad, C. M. Albert, D. Ardissino, I. M. Armean, E. G. Atkinson, G. Atzmon, J. Barnard, S. M. Baxter, L. Beaugerie, E. J. Benjamin, D. Benjamin, M. Boehnke, L. L. Bonnycastle, E. P. Bottinger, D. W. Bowden, M. J. Bown, H. Brand, S. Brant, T. Brookings, S. Bryant, S. E. Calvo, H. Campos, J. C. Chambers, J. C. Chan, K. R. Chao, S. Chapman, D. I. Chasman, R. Chisholm, J. Cho, R. Chowdhury, M. K. Chung, W. K. Chung, K. Cibulskis, B. Cohen, K. M. Connolly, A. Correa, B. B. Cummings, D. Dabelea, J. Danesh, D. Darbar, P. Darnowsky, J. Denny, R. Duggirala, J. Dupuis, P. T. Ellinor, R. Elosua, J. Emery, E. England, J. Erdmann, T. Esko, E. Evangelista, D. Fatkin, J. Florez, A. Franke, J. Fu, M. Färkkilä, K. Garimella, J. Gentry, G. Getz, D. C. Glahn, B. Glaser, S. J. Glatt, D. Goldstein, C. Gonzalez, L. Groop, S. Gudmundsson, A. Haessly, C. Haiman, I. Hall, C. L. Hanis, M. Harms, M. Hiltunen, M. M. Holi, C. M. Hultman, C. Jalas, M. Kallela, D. Kaplan, J. Kaprio, S.

Kathiresan, E. E. Kenny, B.-J. Kim, Y. J. Kim, D. King, G. Kirov, J. Kooner, S. Koskinen, H. M. Krumholz, S. Kugathasan, S. H. Kwak, M. Laakso, N. Lake, T. Langsford, K. M. Laricchia, T. Lehtimäki, M. Lek, E. Lipscomb, R. J. F. Loos, W. Lu, S. A. Lubitz, T. T. Luna, R. C. W. Ma, G. M. Marcus, J. Marrugat, K. M. Mattila, S. McCarroll, M. I. McCarthy, J. L. McCauley, D. McGovern, R. McPherson, J. B. Meigs, O. Melander, A. Metspalu, D. Meyers, E. V. Minikel, B. D. Mitchell, V. K. Mootha, A. Naheed, S. Nazarian, P. M. Nilsson, M. C. O'Donovan, Y. Okada, D. Ongur, L. Orozco, M. J. Owen, C. Palmer, N. D. Palmer, A. Palotie, K. S. Park, C. Pato, A. E. Pulver, D. Rader, N. Rahman, A. Reiner, A. M. Remes, D. Rhodes, S. Rich, J. D. Rioux, S. Ripatti, D. M. Roden, J. I. Rotter, N. Sahakian, D. Saleheen, V. Salomaa, A. Saltzman, N. J. Samani, K. E. Samocha, A. Sanchis-Juan, J. Scharf, M. Schleicher, H. Schunkert, S. Schönherr, E. G. Seaby, S. H. Shah, M. Shand, T. Sharpe, M. B. Shoemaker, T. Shyong, E. K. Silverman, M. Singer-Berk, P. Sklar, J. T. Smith, J. G. Smith, H. Soininen, H. Sokol, R. G. Son, J. Soto, T. Spector, C. Stevens, N. O. Stitziel, P. F. Sullivan, J. Suvisaari, E. S. Tai, K. D. Taylor, Y. Y. Teo, M. Tsuang, T. Tuomi, D. Turner, T. Tusie-Luna, E. Vartiainen, M. Vawter, L. Wang, A. Wang, J. S. Ware, H. Watkins, R. K. Weersma, B. Weisburd, M. Wessman, N. Whiffin, J. G. Wilson, R. J. Xavier, A. O'Donnell-Luria, M. Solomonson, C. Seed, A. R. Martin, M. E. Talkowski, H. L. Rehm, M. J. Daly, G. Tiao, B. M. Neale, D. G. MacArthur, K. J. Karczewski, Genome Aggregation Database Consortium, A genomic mutational constraint map using variation in 76,156 human genomes. *Nature* **625**, 92–100 (2024).

59. J. Attig, G. R. Young, J. P. Stoye, G. Kassiotis, Physiological and pathological transcriptional activation of endogenous retroelements assessed by RNA-sequencing of B lymphocytes. *Front. Microbiol.* **8**, 2489 (2017).

60. J. T. Robinson, H. Thorvaldsdóttir, W. Winckler, M. Guttman, E. S. Lander, G. Getz, J. P. Mesirov, Integrative genomics viewer. *Nat. Biotechnol.* **29**, 24–26 (2011).

61. U. Raudvere, L. Kolberg, I. Kuzmin, T. Arak, P. Adler, H. Peterson, J. Vilo, g:Profiler: A web server for functional enrichment analysis and conversions of gene lists (2019 update). *Nucleic Acids Res.* **47**, W191–W198 (2019).

62. T. Plowman, T. Hofland, C. Hall, R. Thompson, J. Pape, K. W. Ng, L. Doglio, G. Kassiotis, Primate retroelement exonization and sexually dimorphic IL13RA1 transcription tune type 2 immune responses. *Sci. Immunol.* **10**, eadr1105 (2025).

63. O. C. K. Inge, E. Copin, J. Cornwall-Scoones, B. Gharibi, I. Rodriguez-Hernandez, P. Soro-Barrio, M. Strom, P. Chakravarty, J. Briscoe, S. D. M. Santos, Combinatorial BMP4 and activin direct the choice between alternate routes to endoderm in a stem cell model of human gastrulation. *Dev. Cell* **60**, 3304–3320.e9 (2025).

64. I. Scheinin, D. Sie, H. Bengtsson, M. A. van de Wiel, A. B. Olshen, H. F. van Thuijl, H. F. van Essen, P. P. Eijk, F. Rustenburg, G. A. Meijer, J. C. Reijneveld, P. Wesseling, D. Pinkel, D. G. Albertson, B. Ylstra, DNA copy number analysis of fresh and formalin-fixed specimens by shallow whole-genome sequencing with identification and exclusion of problematic regions in the genome assembly. *Genome Res.* **24**, 2022–2032 (2014).

65. X. Huang, X. Tang, X. Bai, H. Li, H. Tao, J. Wang, Y. Li, Y. Sun, Y. Zheng, X. Xu, L. Wang, Y. Ding, M. Lu, P. Zhou, X. Bo, H. Li, H. Chen, dbEmbryo multi-omics database for analyses of synergistic regulation in early mammalian embryo development. *Genome Res.* **32**, 1612–1625 (2022).

66. L. Zheng, P. Liang, C. Long, H. Li, H. Li, Y. Liang, X. He, Q. Xi, Y. Xing, Y. Zuo, EmAtlas: A comprehensive atlas for exploring spatiotemporal activation in mammalian embryogenesis. *Nucleic Acids Res.* **51**, D924–d932 (2023).

67. J. R. Canizo, C. Zhao, S. Petropoulos, The guinea pig serves as an alternative model to study human preimplantation development. *Nat. Cell Biol.* **27**, 696–710 (2025).

68. F. A. Ran, P. D. Hsu, J. Wright, V. Agarwala, D. A. Scott, F. Zhang, Genome engineering using the CRISPR-Cas9 system. *Nat. Protoc.* **8**, 2281–2308 (2013).

69. O. Tange, GNU Parallel: The command-line power tool. *USENIX Mag.* **36**, 42–47 (2011).

70. A. M. Bolger, M. Lohse, B. Usadel, Trimmomatic: A flexible trimmer for Illumina sequence data. *Bioinformatics* **30**, 2114–2120 (2014).

71. A. R. Quinlan, I. M. Hall, BEDTools: A flexible suite of utilities for comparing genomic features. *Bioinformatics* **26**, 841–842 (2010).

72. D. Kim, J. M. Paggi, C. Park, C. Bennett, S. L. Salzberg, Graph-based genome alignment and genotyping with HISAT2 and HISAT-genotype. *Nat. Biotechnol.* **37**, 907–915 (2019).

73. P. Danecek, J. K. Bonfield, J. Liddle, J. Marshall, V. Ohan, M. O. Pollard, A. Whitwham, T. Keane, S. A. McCarthy, R. M. Davies, H. Li, Twelve years of SAMtools and BCFtools. *GigaScience* **10**, giab008 (2021).

74. Y. Liao, G. K. Smyth, W. Shi, featureCounts: An efficient general purpose program for assigning sequence reads to genomic features. *Bioinformatics* **30**, 923–930 (2014).

75. M. I. Love, W. Huber, S. Anders, Moderated estimation of fold change and dispersion for RNA-seq data with DESeq2. *Genome Biol.* **15**, 550 (2014).
